# Metabolome and Transcriptome Association Analysis Reveals Mechanism of Synthesis of Nutrient Composition in Quinoa (*Chenopodium quinoa* Willd.) Seeds

**DOI:** 10.3390/foods13091325

**Published:** 2024-04-26

**Authors:** Jindan Yang, Yiyun Wang, Jiayi Sun, Yuzhe Li, Renbin Zhu, Yongjie Yin, Chuangyun Wang, Xuebin Yin, Lixia Qin

**Affiliations:** 1 College of Agronomy, Shanxi Agricultural University, Taiyuan 030031, China; 15891127588@163.com (J.Y.); 19131320527@163.com (Y.W.); 13583535835@163.com (J.S.); 18147555421@163.com (Y.L.); 18782650829@163.com (Y.Y.); wcytyd@sxau.edu.cn (C.W.); 2 School of Earth and Space Sciences, University of Science and Technology of China, Hefei 230036, China; zhurb@ustc.edu.cn; 3 Suzhou Selenium Valley Technology Co., Ltd., Suzhou 215100, China; xbyin@ahstu.edu.cn; 4 Anhui Province Key Laboratory of Functional Agriculture and Functional Food, Anhui Science and Technology University, Chuzhou 239000, China

**Keywords:** quinoa, nutritional content, metabolomics, transcriptomics, candidate genes

## Abstract

Quinoa (*Chenopodium quinoa* Willd.) seeds are rich in nutrition, superior to other grains, and have a high market value. However, the biosynthesis mechanisms of protein, starch, and lipid in quinoa grain are still unclear. The objective of this study was to ascertain the nutritional constituents of white, yellow, red, and black quinoa seeds and to employ a multi-omics approach to analyze the synthesis mechanisms of these nutrients. The findings are intended to furnish a theoretical foundation and technical support for the biological breeding of quinoa in China. In this study, the nutritional analysis of white, yellow, red, and black quinoa seeds from the same area showed that the nutritional contents of the quinoa seeds were significantly different, and the protein content increased with the deepening of color. The protein content of black quinoa was the highest (16.1 g/100 g) and the lipid content was the lowest (2.7 g/100 g), among which, linoleic acid was the main fatty acid. A combined transcriptome and metabolome analysis exhibited that differentially expressed genes were enriched in “linoleic acid metabolism”, “unsaturated fatty acid biosynthesis”, and “amino acid biosynthesis”. We mainly identified seven genes involved in starch synthesis (LOC110716805, LOC110722789, LOC110738785, LOC110720405, LOC110730081, LOC110692055, and LOC110732328); five genes involved in lipid synthesis (LOC110701563, LOC110699636, LOC110709273, LOC110715590, and LOC110728838); and nine genes involved in protein synthesis (LOC110710842, LOC110720003, LOC110687170, LOC110716004, LOC110702086, LOC110724454 LOC110724577, LOC110704171, and LOC110686607). The data presented in this study based on nutrient, transcriptome, and metabolome analyses contribute to an enhanced understanding of the genetic regulation of seed quality traits in quinoa, and provide candidate genes for further genetic improvements to improve the nutritional value of quinoa seeds.

## 1. Introduction

Quinoa (*Chenopodium quinoa* Willd.) is a summer annual dicotyledonous herbaceous crop of the Amaranthaceae family [1], and is the only plant recognized by the Food and Agriculture Organization (FAO) as being capable of meeting the basic nutritional needs of the human body independently. It is, thus, recommended as the most suitable “total nutritional food” for humans [2]. Quinoa seeds are superior to other grains due to their higher contents of protein, fat, vitamins, minerals, and lysine [3]. They are also rich in plant-active substances such as phenols, flavonoids, saponins, choline, and phytosterols. Regular consumption can help to prevent various metabolic diseases and maintain health [4]. Proteins and lipids are primarily stored in proteomes and liposomes, respectively, both of which are present in embryonic cells. The endosperm, which serves as the storage tissue for carbohydrates, primarily exists in the form of starch [5]. Quinoa maintains a balance among oil, protein, and carbohydrates [6] with a protein content of 14–15%, generally higher than barley, corn, and other grains [7]. Amylopectin is present in mature seeds, and the endosperm contains a very high starch content. The synthesis of starch is associated with several proteins, some of which remain in the starch structure post-synthesis, such as granular-bound starch synthase (GBSS), soluble starch synthase (SSS), and starch branching and debranching enzymes [8]. Quinoa seeds are rich in eight essential amino acids for humans, one of which is an infant essential amino acid, which is easily absorbed by the body [9]. This amino acid composition is particularly rich in lysine, histidine, and methionine [10]. Its high nutritional value contributes to food security in the 21st century, leading the FAO to list it as a “Smart Food of the Future” and promote it globally. In 2013, it was designated as the “International Year of Quinoa” [11,12]. In addition, quinoa is nutritionally rich, a potent source of bioactive components, and holds significant nutritional health products and market value. 

A correlation analysis of quinoa based on transcriptomics and metabolomics revealed that the Heat Stress transcription Factor (HSF) participates in responding to high-temperature stress [13]. Multi-omics studies indicate that lysine biosynthesis is related to amino acid metabolism and starch and sucrose metabolism, and candidate genes involved in lysine synthesis have been identified [14]. Additionally, the genetic differences between flavonoids and procyanidins in seed quality have been found, and key candidate genes of triterpenoid saponins biosynthesis and plant hormone stress-related genes involved in abiotic stresses during seed germination have also been identified [15,16]. Currently, there are few studies on multiomics analyses of the nutrient synthesis mechanism of quinoa seeds. In partiuclar, the regulatory mechanism of nutrient synthesis in quinoa seeds has not been reported. The yield and quality of quinoa seeds are generally influenced by genetic, environmental, and biological factors, particularly the differences among quinoa varieties with different colors. This study aimed to quantify the nutrient profiles of quinoa seeds across four varied colors. Additionally, the underlying mechanisms responsible for the differential synthesis of nutrients in these four types of quinoa seeds were investigated using transcriptomic and metabolomic approaches. The key candidate genes involved in the synthesis pathway of starch, fat, and protein in quinoa seeds were identified, providing a theoretical basis and technical support for the biological breeding of quinoa.

## 2. Materials and Methods

### 2.1. Plant Materials

Quinoa cultivars, encompassing White (W), Yellow (Y), Red (R), and Black (B) varieties, were cultivated in Nanshe Village, Jingle County, within the administrative bounds of Xinzhou City, Shanxi Province (geographical coordinates: 112.04° E, 38.28° N). This agricultural site stands at an elevation of 1649 m and is characterized by a temperate continental climate, providing an annual rainfall range from 400 to 600 mm that is conducive to the optimal growth of quinoa. Quinoa plants that flowered on the same day were marked. Seeds from three biological replicates were collected at four different developmental stages (early, middle, late, and imminent maturity stages, represented as 20, 30, 40, and 50 days post anthesis (dpa) for four different color seeds, respectively) and then immediately placed on ice, and the sepals were removed with tweezers. The seeds were then transferred into plastic tubes and submerged in liquid nitrogen until the volume reached 2–3 mL, then they were subsequently stored at −80 °C. The seeds were sampled at approximately the same time of day (4–6 h post-light period) to minimize potential differences due to daily fluctuations in gene expression and nutrient levels. The seed samples were ground and then frozen in liquid nitrogen, with the resulting powder stored at −80 °C for future use. 

### 2.2. Determination of Nutritional Components of Quinoa Seeds with Four Different Colors

The protocol for the determination of protein content was conducted as follows: A sample of 0.5 g of quinoa seeds was processed utilizing the Kjeldahl method [17] for nitrogen analysis. Subsequently, the processed sample was subjected to a digestion procedure within a high-temperature furnace operating at 420 °C for a duration of 80 min. Upon the completion of the digestion phase, the digestion tube was carefully extracted from the furnace and allowed to cool to ambient temperature in a room environment. Ultimately, the protein content of the cooled sample was quantified using an automated Kjeldahl (NHRI-EQ-P5004-2, Foss Analytical AB) nitrogen analyzer, with the resulting data meticulously recorded for analysis.

To quantify the lipid content, the following protocol was employed: a 2.5 g aliquot of lyophilized quinoa seeds was subjected to Soxhlet extraction [18]. The seed powder was encapsulated in filter paper and inserted into a Soxhlet apparatus. Subsequently, a 7 h continuous reflux extraction was conducted using petroleum ether with a boiling point of 80℃. Upon the completion of the extraction phase, the petroleum ether was reclaimed and the collection vessel was transferred to an electronic hot air oven set at 105℃ for desiccation to a constant mass. Following cooling to ambient temperature, the vessel was re-weighed to determine the lipid content. This method is widely utilized due to its precision in lipid quantification. 

The starch content of the quinoa seeds (50 mg) was determined using the total starch content kit (Megazyme, Wicklow, Ireland). One milliliter of 80% ethanol (*v*/*v*) was added and each sample was incubated using a BioShake (BioShake XP, Europe, Germany) at 80 °C and 1000 rpm for 20 min. After centrifugation at 13,000× *g* for 10 min, the supernatant was carefully discarded in order to remove soluble sugars. Centrifugation at 13,000× *g* for 10 min and the removal of the supernatant were repeated before the pellet was resuspended in 40 μL of 50% (*v*/*v*) ethanol and 400 μL of 50 mM Mops buffer (pH 7.0). Ten microliters of thermostable α-amylase were added and incubated at 100 °C and 1000 rpm for 10 min. Then, 600 μL of sodium acetate buffer (pH 4.5) and 10 μL of amyloglucosidase were added and vortexed for 5 s. This was followed with a 50 °C incubation using a BioShake at 1000 rpm for a minimum of 60 min. After centrifugation at 13,000× *g* for 10 min, 20 μL aliquots in duplicate with appropriate dilutions were transferred to a 96-well flatted bottom plate with 180 μL of GOPOD. The absorbance was read using a SPECTRO star Nano Microplate Reader (BMG LABTECH, Australia) at 510 nm after 20 min incubation at 50 °C. The 1 mg/mL of glucose standard from the kit was used to generate a standard curve. 

To quantify the amino acid composition of the quinoa seeds, an automated amino acid analyzer was employed [19]. The analytical procedure commenced with the weighing, desiccation, and pulverization of a representative sample of quinoa seeds. Subsequently, 10 mL of a 6 mol/L hydrochloric acid solution was added to the sample, which was then subjected to hydrolysis in an oven at 110 °C for a duration of 22 h. Following hydrolysis, the sample was cooled and the resultant hydrolysate was filtered to remove any particulate matter, after which, the filtrate was adjusted to a final volume of 50 mL. Next, a 1 mL aliquot of the filtered solution was evaporated to dryness using a nitrogen blowing apparatus. The dried residue was subsequently reconstituted in a sodium citrate buffer solution with a pH of 2.2 and the solution was then passed through a 0.22 µm porosity filter membrane to ensure clarity, in preparation for analysis. The prepared sample solution was finally introduced into the automatic amino acid analyzer (NHRI-EQ-C6096NHRI-EQ-C6015, SYKAM) for quantitative analysis. The analyzer utilized a comparison of the peak areas from the sample with those of known amino acid standards to calculate the concentration of each amino acid present in the quinoa seed sample. 

### 2.3. Extensive Targeted Metabonomic Detection and Analysis

The samples were vacuum freeze-dried in a freeze-dryer (Scientz-100F) and ground with a grinder (MM 400, Retsch) at 30 Hz for 1.5 min until a powder was obtained. In total, 100 mg of the powder was dissolved in 1.2 mL of 70% methanol extract, vortexed every 30 min for 30 sec, six times in total, and then placed in a 4 °C refrigerator overnight. The sample was centrifuged at 12,000 rpm for 10 min, filtered with a microporous membrane (0.22 μm pore size), and stored in an injection bottle for UPLC-MS/MS analysis. The chromatographic column was Agilent SB-C18 (1.8 μm; 2.1 mm × 100 mm). The mobile phase A was ultrapure water (with 0.1% formic acid added) and the mobile phase B was acetonitrile (with 0.1% formic acid added). The elution gradient, flow rate, column temperature, and injection volume were set as described. LIT and triple quadrupole (QQQ) were scanned on a triple quadrupole linear ion trap mass spectrometer (Q TRAP) and AB4500 Q TRAP UPLC/MS/MS system. The system has an ESI Turbo ion spray interface, and positive and negative ion modes are controlled by the analyst 1.6.3 software (AB Sciex). The operating parameters of the ESI source are as described. Instrument quality calibration was performed using 10 μmol/L of polypropylene glycol solution in QQQ mode and 10 μmol/L of polypropylene glycol solution in LIT mode. MRM mode was used to obtain metabolite spectrum analysis data of different samples, and peak area integration and correction were carried out [20]. 

### 2.4. Transcriptome Data Analysis

High-quality total RNA was extracted and sequenced as previously described [21,22]. Three biological replicates were performed for each sample type, yielding 48 transcriptome libraries. The original RNA sequencing reads were mapped to the quinoa genome using HISAT2 [23]. String Tie was used to calculate the gene expression levels. DEseq2 was used to analyze differentially expressed genes among the sample groups [24], with a threshold value of log2Fold Change >= 1 and FDR < 0.05. The functional annotation and pathway analysis of these differentially expressed genes were based on the Gene Ontology (GO) and KEGG Ortholog (KO) databases. A statistical analysis of RNA-seq was conducted using a combination of two fold changes between the means of the biological replicates and a false discovery rate below 0.05 (*p* < 0.05).

### 2.5. qRT-PCR Analysis 

The reliability of the transcriptome sequencing results was confirmed by qRT-PCR. The total RNA was extracted from 48 samples (W1, W2, W3, W4, Y1, Y2, Y3, Y4, R1, R2, R3, R4, B1, B2, B3, and B4) as previously described [21], and reverse transcription was performed using the first strand cDNA of the RNA. Each sample, consisting of 0.5 g of fresh seedlings, was ground in liquid nitrogen, and the total RNA was extracted following the kit’s instructions. For each sample, 1 μg of the total RNA was reverse transcribed into cDNA as per the kit’s instructions. qRT-PCR was conducted using the SuperReal PreMix Plus (SYBR Green) kit (FP205, TIANGEN, Beijing, China) and a real-time PCR detection system (480II, Roche, Switzerland). CqActin1 and CqActin2 were selected as internal reference genes [25], and the gene-specific primers, designed using Beacon Designer 7.9, are listed in Table S1. 

Cutadapt (v2.7) software were used to filter the sequence data with the connector at the 3′ end and reads with a mean quality score of > Q20 to yield a high-quality sequence for further functional enrichment analyses, including a Principal Component Analysis (PCA), heat map, Pearson correlation, gene ontology (GO), and KEGG. The statistical analysis of RNA-seq was conducted using a combination of two fold changes between the means of the biological replicates and a false discovery rate below 0.05 (*p* < 0.05).

## 3. Results

### 3.1. Analysis of Nutritional Components of Quinoa Seeds with Different Colors

**Figure 1:**
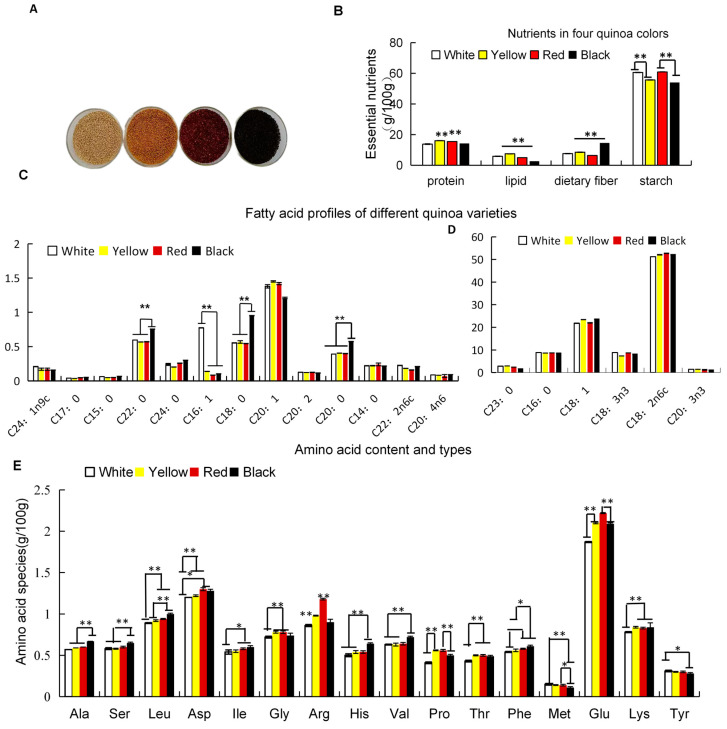
(**A**) Seed photos of quinoa with four colors at the mature stage; (**B**) nutritional content of seeds at the mature stage; (**C**,**D**) composition and content of amino acids in mature seeds; and (**E**) fatty acid profile of mature seeds. Independent *t*-tests revealed that the expression levels of different color quinoa seeds exhibited significant differences (*, *p* < 0.05) or highly significant differences (**, *p* < 0.01).

To investigate the nutritional components of the quinoa seeds during development, we cultivated four quinoa genotypes in the same environment and sampled quinoa seeds at the mature stage (Figure 1A). The results of the determination and analysis revealed that carbohydrates were the primary nutrient in all quinoa samples (Figure 1B), followed by protein and fat. Among them, the black variety had the highest protein content of 16.1 g/100 g and the lowest fat and starch content (2.7 g/100 g and 54.1 g/100 g, respectively). The protein content increased with the color intensity, with the white variety having the lowest protein content of 13.8 g/100 g. The yellow variety had the highest fat content of 7.5 g/100 g, which was 2.7 times that of the black variety, while the white variety was 1.6% lower than the yellow variety. The red variety had the highest starch content of 60.9 g/100 g, which was 6.8% higher than that of the black varieties, while the starch content of the white varieties was 4.9% higher than that of the yellow varieties. 

To delve into the variations in protein and lipid qualities across the different colored quinoa genotypes, we conducted a comprehensive analysis of the amino acid profiles and fatty acid compositions within the total protein fractions of the quinoa seeds exhibiting four distinct hues. (Figure 1C,D), the teosanoenoic acid (C24:1n9c) content in the white quinoa was 0.208% higher compared to the other three varieties of quinoa. Additionally, the behenic acid (C22:0) content in the black quinoa was 0.756% higher than that of the other three varieties. Moreover, the black quinoa exhibited significantly higher levels of stearic acid (C18:0) and arachidonic acid (C20:0), with increases of 0.952% and 0.574%, respectively, when compared to the other three varieties. Furthermore, the yellow quinoa displayed a higher concentration of arachidonic acid (C20:1) than the other three varieties at 1.45%. Lastly, the white quinoa had a greater amount of palmitoleic acid (C16:1) compared to the yellow, red, and black quinoa at 0.774%. Linoleic acid (C18: 2), oleic acid (C18: 1), and palmitic acid (C16: 0) accounted for more than 90% of all the detected fatty acids. Linoleic acid was the primary fatty acid, accounting for 50–60% of the fatty acid content.

The black quinoa exhibited the highest levels of alanine, serine, leucine, and isoleucine content, which increased proportionally with the intensity of its color. Conversely, the red quinoa displayed the highest concentrations of aspartate (1.3 g/100 g), arginine (1.18 g/100 g), and glutamic acid (2.22 g/100 g). Among all colors, the black quinoa demonstrated higher quantities of histidine, valine, and phenylalanine. The white quinoa contained elevated amounts of methionine (0.15 g/100 g) and tyrosine (0.31 g/100 g) compared to other varieties, including the yellow quinoa (0.01 g/100 g) (Figure 1E).

In general, the nutritional analysis data of the developing seeds revealed genotypic differences in the quinoa seeds, identified different proportions of main seed storage compounds, and found that the black quinoa had the highest protein content and the lowest fat content. Therefore, the four genotypes of seeds were further characterized by metabolome and transcriptome analyses, aiming to identify the differential metabolites and differential genes that determined the nutrients of the seeds.

### 3.2. Analysis of Differential Metabolites in Quinoa Seeds with Four Different Colors

To further investigate the differences in the nutritional components in the mature quinoa seeds of different colors, we utilized UPLC-MS/MS to detect related metabolites and the compositions of differential metabolites in the seeds of four quinoa varieties. A total ionic current (TIC) analysis of the QC samples was employed to verify the consistency of the metabolite extraction and detection. The TIC curves overlapped with metabolite detection results (Figure S1A,B). When the same sample was identified at different times, the retention time and peak intensity remained consistent, indicating signal stability. The instrument’s stability provides an essential guarantee for the repeatability and reliability of the data. The Pearson correlation coefficient between repeated samples ensures a good repeatability (Figure S1C). 

**Figure 2:**
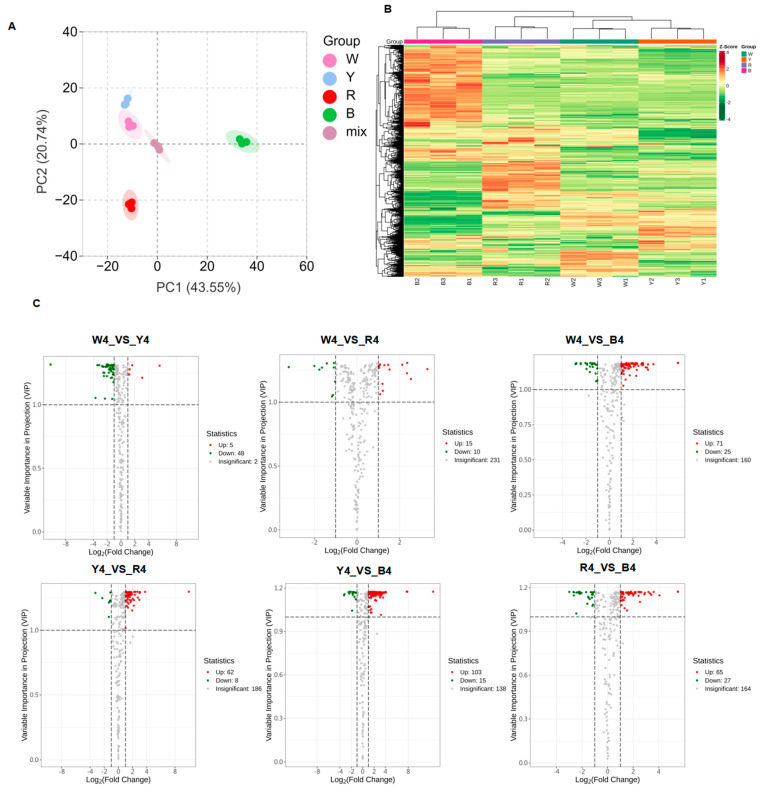
(**A**–**C**) Principal component analysis of 12 metabonomics data; volcanic maps of differential metabolites in contrast with white and black, red and black, white and red, white and yellow, yellow and black, and yellow and red, each dot on the graph corresponds to a metabolite, with distinct colors coding for different regulatory patterns: green dots represent down-regulated differential metabolites, red dots indicate up-regulated differential metabolites, and gray dots denote metabolites that are detected but do not show significant differences between the two sample groups. The horizontal axis plots the logarithm (base 2) of the fold change in the relative content of a metabolite between the two groups of samples (log2FC).

After quality assessment, 772 metabolites were preliminarily identified, including 417 primary metabolites (such as lipids, amino acids and their derivatives, nucleotides and their derivatives, and organic acids, etc.) and 265 secondary metabolites (such as flavonoids, phenolic acids, alkaloids, terpenes, lignans and coumarins, quinones, and tannins, etc.) (Figure S1D). The principal component analysis results indicated there were significant differences in the nutrient metabolites among the four quinoa species (Figure 2A). The hierarchical clustering heat map analysis clearly divided the 12 samples into 4 groups (Figure 2B). Moreover, the relative contents of metabolites in the red quinoa significantly differed from those in the white, yellow, and black quinoa, indicating significant differences in grain composition among the four quinoa varieties. Therefore, the metabolite profiles of the different quinoa varieties had a strong consistency. A total of 139 lipids were identified, with lysophosphatidylcholine (16:0), lysophosphatidylcholine (18:1), lysophosphatidylethanolamine (LPE) (18:2) (2n isomer), lysophosphatidylethanolamine 18:2, and lysophosphatidylcholine 18:1 (2n isomer) being the highest among the four quinoa varieties. 

A partial least squares discriminant analysis was conducted on the four groups of samples, revealing that Q2 of all the comparison groups in the OPLS-DA model verification diagram (Figure S2A) was greater than 0.9 and *p* < 0.005. This indicates that the model was reliable and stable, thus, it explained well the metabolic changes in the four varieties and could be used for further screening of differential accumulated metabolites by a VIP analysis. An analysis of multiple samples revealed different patterns of metabolite accumulation. To identify the differential metabolites from the four quinoa varieties, we defined the metabolites with Fold Change ≥ 2 and Fold Change ≤ 0.5 and VIP ≥ 1 as differential metabolites (Table S2), among which, there were 118 differential metabolites (yellow_VS_black), 96 (white_VS_black), 92 (black_VS_red), 70 (yellow_VS_red), 53 (white_VS_yellow), and 25 (white_VS_red), respectively (Figure 2C). 

**Figure 3:**
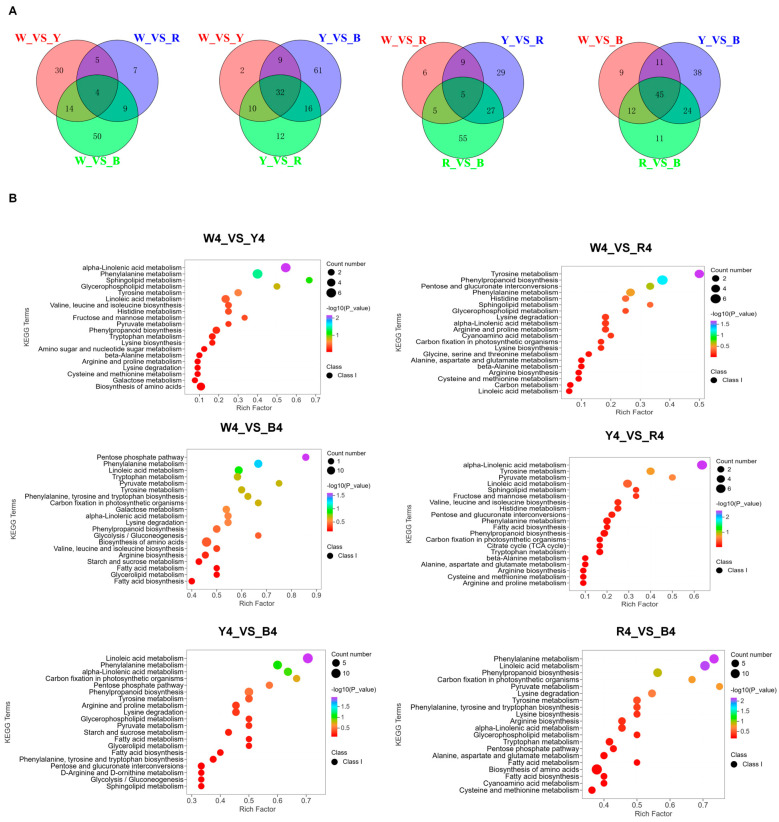
(**A**,**B**) Venn diagrams between different contrasts, each circle represents a comparison group. The numbers within the overlapping areas between circles indicate the count of differential metabolites shared between the respective comparison groups. Conversely, the numbers within the non-overlapping areas of each circle represent the count of differential metabolites that are unique to each individual comparison group. KEGG enrichment bubble diagram between different comparisons, the horizontal axis of the graph depicts the Rich Factor associated with each pathway, while the vertical axis identifies the pathway names. The color gradient of the points is indicative of the *p*-value, with shades of red signifying more significant enrichment. Additionally, the size of the dots is proportional to the number of differentiated metabolites that are enriched within each pathway.

The lipid thermogram is shown in Figure S2B, which can be divided into four categories according to the accumulation mode of lipid substances. The relative content of lipids in the black quinoa was the highest, compared with the other three different color quinoas. In addition, based on a cluster thermograph analysis, proteins can be divided into four main clusters (Figure S2C), where cluster I and cluster II contained 55 kinds of amino acids, and most of the metabolites were the highest in black quinoa. The starch content of the red varieties in cluster 2 was higher than that in the white and yellow varieties (Figure S2D). A Venn diagram analysis showed (Figure 3A) that there were 19 different metabolites in the white quinoa and 29 different metabolites in the yellow quinoa (W_VS_Y), there were 10 different metabolites in the white quinoa and 29 different metabolites in the black quinoa (W_VS_B), there was 1 different metabolite in the yellow quinoa and 5 different metabolites in the red quinoa (Y_VS_R), there were 11 different metabolites in the yellow quinoa and 20 different metabolites in the black quinoa (Y_VS_B), and there were 12 different metabolites in the red quinoa and 17 different metabolites in the black quinoa (R_VS_B). In addition, we found that changes in lipid, starch, and protein in different quinoa varieties were the most significant. Comparing white and black amino acids, we found that the change factors of 69 amino acids were between 1.05 and 13.47 folds (up-regulated 21 kinds and down-regulated 9 kinds). In the red quinoa, the top three metabolites were 5, 8-dihydroxy-9, 12-octadecadienoic acid (45.41 folds), 9-hydroxy-12-oxo-15 (Z)-octadecadienoic acid (44.93 folds), and N-acetyl-L-tyrosine (16.64 folds). The majority of metabolites in the white and red quinoa were 3, 4-dihydroxy-L-phenylalanine and L-alanyl-L-alanine. The difference in the metabolites of yellow and black lipids ranged from 0.03 to 7.82 folds. The top three lipids, namely 9-hydroxy-12-oxo-15 (Z)-octadecenoic acid (7.82 folds), 5, 8-dihydroxy-9, 12-octadecadienoic acid (7.73 folds), and 9-hydroxy-10, 12, 15-octadecatrienoic acid (4.08 folds), were highest in the black varieties (Figure S2E). Therefore, the results indicate significant differences in the amino acid, lipid, and starch components in quinoa seeds of different colors. Thus, we can monitor the metabolomic changes among different colors to select and cultivate quinoa seeds rich in specific nutrients.

The KEGG enrichment analysis of the different metabolites in the grain varieties of different colors showed that W_VS_Y, W_VS_R, W_VS_B, Y_VS_R, Y_VS_B, and R_VS_B involved 44, 47, 80, 44, 69, and 75 pathways, respectively. The KEGG enrichment analysis of the different metabolites in the different color grain varieties showed that three of the six control groups (W_VS_B, Y_VS_B, and R_VS_B) were highly enriched in metabolite pathways (80, 69, and 75), respectively. The top 20 pathways in the six comparison groups were related to sphingolipid metabolism, α-linolenic acid metabolism, glycerophospholipid metabolism, phenylalanine metabolism, tyrosine metabolism, histidine metabolism, pentose phosphate pathway, and linoleic acid metabolism (Figure 3B). The metabolisms of sphingolipids, glycerophospholipids, and α-linolenic acid are related to lipid synthesis, while phenylalanine, tyrosine, and histidine are related to amino acid synthesis. The KEGG database was used to analyze the differential metabolites of protein, starch, and lipid to further characterize the interactions between components. The results of the GO enrichment analysis are consistent with “α-linolenic acid metabolism”, “phenylpropanoid biosynthesis”, “amino acid biosynthesis”, and “linoleic acid metabolism” (Figure S2F).

### 3.3. Comparative Transcriptome Analysis of Quinoa Genotypes

A comparative transcriptome analysis was conducted on the four quinoa genotypes using RNA sequencing. This was performed on three biological replicates of seed tissues at four developmental stages: the prophase, metaphase, anaphase, and near-mature stages. The aim was to detect differences in gene expression and verify gene function. After quality inspection, over 92% of the sample reads were successfully mapped to the quinoa reference genome. The transcriptome data, as shown in Table S3, met the requirements for subsequent analysis. The correlation heat map and cluster heat map (Figure S3A,B) demonstrated a good biological repeatability and significant differences among the samples. 

**Figure 4:**
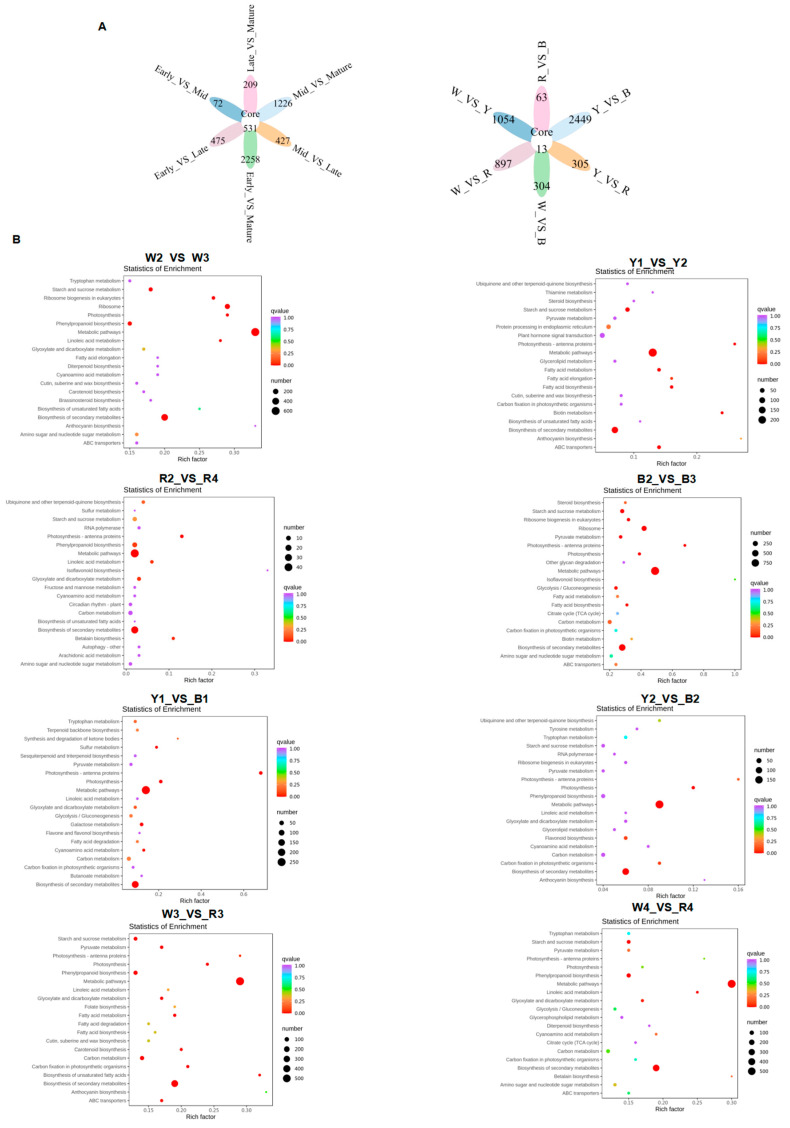
(**A**,**B**) Enrichment and analysis of differentially expressed genes. Venn diagrams of different varieties at the same growth stage and different growth stages of the same variety; KEGG enrichment map. The enrichment scatter plot depicts KEGG pathways on the vertical axis and the Rich Factor on the horizontal axis. A higher Rich Factor indicates a greater degree of enrichment. The size of the dot corresponds to the number of differentially expressed genes enriched in the pathway; larger dots signify more genes. The color of the dot increasingly trending towards red indicates more significant enrichment.

The screening conditions were set at log2Fold Change >= 2 and FDR < 0.05 to compare the quinoa varieties at different stages (Table S4). The results revealed varying numbers of differentially expressed genes (DEGs) at different stages and between different varieties (Figure 4A). A pairwise comparative analysis of different stages in the white quinoa revealed a significant number of DEGs. Similar analyses were conducted for the yellow and red quinoa, revealing varying numbers of DEGs at different stages. The gene expression level of the four quinoa cultivars changed significantly during maturation. The down-regulated DEGs were more abundant than the up-regulated DEGs in the early and middle stages for the white, yellow, and red quinoa. However, for the black quinoa, the down-regulated DEGs were more abundant at all stages. Further comparisons were made between each two varieties of quinoa with different colors at the middle and later stages, revealing varying numbers of DEGs. The maturation stages of the quinoa varieties with different colors were also compared, resulting in a significant number of DEGs (Figure S3C). To study the biological function of the DEGs, a KEGG enrichment analysis was performed (Figure 4B and Figure S3D).

**Figure 5:**
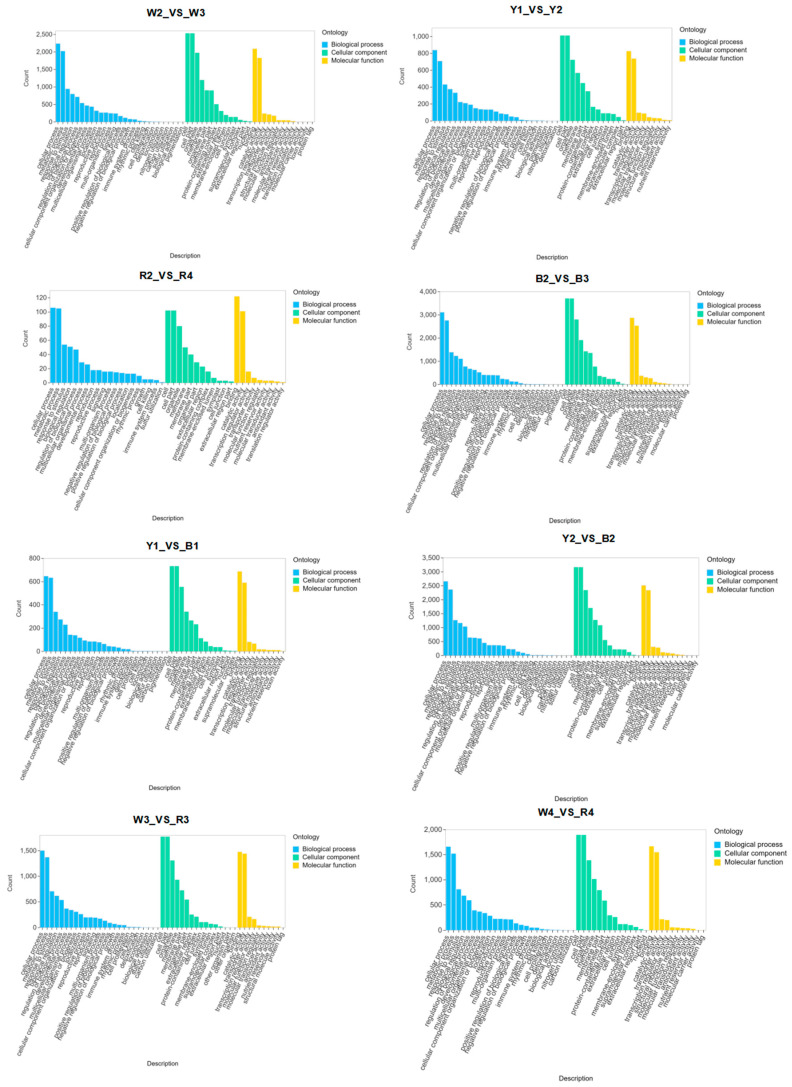
GO enrichment map of different quinoa varieties, the horizontal axis denotes the secondary Gene Ontology (GO) categories, while the vertical axis represents the count of differential genes associated with each GO entry.

The results showed significant enrichment in “α-amylase activity”, “β-amylase activity”, “fatty acid biosynthesis”, “unsaturated fatty acid biosynthesis”, and “phenylpropanoid biosynthesis”. Additionally, GO enrichment (Figure 5 and Figure S3E) showed significant enrichment in several metabolic pathways. These pathways were directly related to the results of the nutritional composition analysis. 

### 3.4. Correlation Analysis of Transcriptome and Metabolome Data

**Figure 6:**
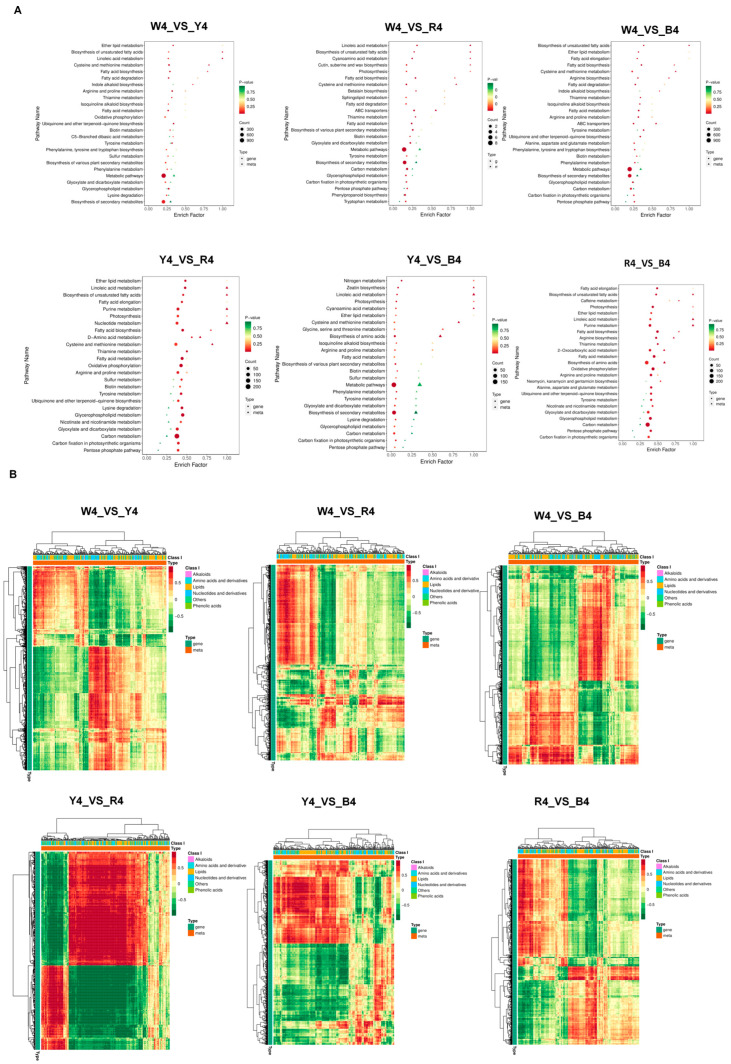
(**A**,**B**) Bubble diagram of KEGG enrichment, the horizontal axis of the graph illustrates the enrichment factors of pathways across different omics datasets, expressed as the ratio of differentially abundant entities to the background population (Diff/Background). The vertical axis lists the names of the metabolic pathways. The color gradient, transitioning from red through yellow to green, signifies the decreasing significance of enrichment, with more intense colors indicating higher significance, as quantified by the negative logarithm of the *p*-value (−log10(*p*-value)). The shape of each bubble distinguishes between different omics types, while the size of the bubble corresponds to the number of differentially abundant metabolites or genes; a larger bubble indicates a greater number of these entities (**A**); Correlation clustering heat map, each row corresponds to a gene, and each column to a metabolite. The color scheme is used to depict the nature of the correlation: red indicates a positive correlation between the gene and the metabolite, while green signifies a negative correlation (**B**).

To gain a deeper understanding of the regulatory mechanisms of the nutritional components in the quinoa seeds, a correlation analysis was conducted on the differential metabolites of the mature seeds and the differentially expressed genes nearing maturity. A nine-quadrant association analysis was employed to identify genes exhibiting the same trend in both the metabonomic and proteomic data. As depicted in (Figure S4A), 14, 72, 234, 578, 157, and 152 genes were identified in six control groups. These genes were located in the third quadrant. The KEGG enrichment bubble map (Figure 6A) revealed significant enrichments in the pathways of “linoleic acid metabolism”, “unsaturated fatty acid biosynthesis”, “fatty acid biosynthesis”, and “amino acid biosynthesis”. A correlation analysis was performed between differential genes and differential metabolites. The results, presented in a cluster thermomap, indicated that amino acids, their derivatives, and lipid metabolites constituted the majority (Figure 6B). 

### 3.5. Differential Expression of Carbon Metabolism and Regulatory Genes in Four Quinoa Cultivars

The KEGG pathway and GO analyses revealed that numerous differentially expressed genes encoded key enzymes and transcription factors involved in nutrient synthesis pathways. These findings corroborate the observed differences in nutrient accumulation during the physiological maturation of the various quinoa varieties in this study. Compared to the black genotype, the red quinoa exhibited the up-regulation of 44 transcripts encoding glycolytic function, including enzymes such as glucose-6-phosphate 1-exoformylase, aldose 1-episomerase, fructokinase 6-phosphate, pyruvate dehydrogenase, fructose bisphosphate aldolase, alcohol dehydrogenase, hexokinase, and pyruvate decarboxylase. Additionally, three out of four transcripts encoding the phosphoenolpyruvate transporter [23] were down-regulated (gene-LOC110725781, gene-LOC110735795, and gene-LOC110725782), while one was up-regulated (gene-LOC110735796). This transporter facilitates the movement of three-carbon groups across the chloroplast membrane (phosphoenolpyruvate/PHOSPHATE translocator 2), primarily utilized in the seed coat during mid-development. Notably, 60 transcripts encoding the Ribulose bisphosphate carboxylase small chain were up-regulated across all varieties compared to other genotypes. This subunit plays a pivotal role in carbon fixation in the Calvin cycle [26]. Two transcripts encoding UDP-glucose pyrophosphorylase (novel.3959 and novel.4763) were up-regulated and worked in conjunction with sucrose phosphate synthase to convert glucose-1-phosphate into UDP-glucose, a process primarily involved in sucrose synthesis. In the cytoplasm of the seed endosperm, UGPase pairs with cytoplasmic ADP-glucose phosphorylase, leading to the direct conversion of UDP-glucose into ADP-glucose for starch synthesis [27,28]. Two transcripts encoding glucose phosphate mutase (gene-LOC110729741 and gene-LOC110732480) were up-regulated. Glucose phosphate mutase catalyzes the mutual transformation between glucose 1-phosphate and glucose 6-phosphate, existing in both plastid and cytoplasmic subtypes, playing a crucial role in starch synthesis [29]. Compared to W3, one transcript encoding phosphoglycerate kinase in W1 (gene-LOC110713524) was up-regulated. Interestingly, another transcript encoding phosphoglycerate kinase in Y1 (gene-LOC110721314) was also up-regulated compared to Y4. This transporter catalyzes the reversible conversion of 1, 3-diphosphoglycerate into para-3-phosphoglycerate and participates in the carbon cycle to fix carbon in plants. In Arabidopsis thaliana, the knockout mutation of phosphoglycerate kinase is characterized by a decrease in photosynthetic capacity and starch content, suggesting that phosphoglycerate kinase plays a significant role in starch synthesis [30,31]. Compared to the yellow genotype, two transcripts encoding transketolase in the red quinoa (gene-LOC110728546 and gene-LOC110721444) were up-regulated. These control photosynthetic carbon fixation and are key enzymes in the tricarboxylic acid cycle [32,33]. Interestingly, a transcript encoding pyruvate kinase (gene-LOC110732515) was also up-regulated in the red quinoa. This enzyme catalyzes the irreversible transfer of high-energy phosphate groups from phosphoenolpyruvate to ADP, synthesizing pyruvate and ATP, and is a key enzyme in glycolysis [34]. Compared to the black quinoa, the yellow quinoa exhibited up-regulation in 4 transcripts encoding granule-bound starch synthase (GBSS), 15 transcripts encoding starch synthase, 12 transcripts encoding α-amylase, and 8 transcripts encoding starch-degrading β-amylase [35]. In contrast to the yellow genotype, the black quinoa showed up-regulation in the transcript encoding the sugar transporter (gene-LOC110687202). Additionally, compared to the late stage of the white genotype, a transcript of the white genotype in the impending mature stage (gene-LOC110705141) was also up-regulated. In Arabidopsis thaliana, three SWEET genes of clade III (SWEET 11, 12, and 15) exhibited temporal and spatial expression during seed development, potentially facilitating the transport of sucrose from the seed coat to the developing embryo. This suggests that SWEET plays a crucial role in seed development by transporting sucrose from the seed coat to the developing embryo [36]. Plastid phosphoglucose mutase impacts starch synthesis and endosperm development in rice [37] (Table S5).

### 3.6. Differential Expression of Lipid Anabolism and Regulatory Genes in Four Quinoa Cultivars

Compared to the yellow genotype, seven transcripts encoding phospholipase (expressed at the chalazal early endosperm) and three transcripts encoding acyltransferase (involved in oil body synthesis) in the black quinoa were up-regulated [38]. Additionally, 15 transcripts encoding lipase in the black quinoa were up-regulated compared to the yellow genotype. These play crucial roles in the initial stage of seed germination. Furthermore, plastid lipase contributes to seed oil biosynthesis [39]. In the red quinoa, two transcripts encoding long-chain acyl-coenzyme A synthetase (gene-LOC110732999, gene-LOC110734248) were up-regulated compared to the yellow genotype. These enzymes are critical in lipid metabolism and participate in oil biosynthesis [40]. A transcript encoding fatty acid desaturase (gene-LOC110738720) was up-regulated in the red genotype compared to the yellow genotype, playing a significant role in the biosynthesis of linoleic and linolenic acids [41]. A transcript encoding the black quinoa (gene-LOC110701189) was up-regulated compared to the yellow genotype. This transcript catalyzes the conversion of glycerol-3-phosphate and long-chain acyl-coenzyme A into lysophosphatidic acid, playing a key role in the regulation of triglyceride and phospholipid synthesis. It also plays a crucial role in the biosynthesis of membrane lipids and storage oils, enhancing the yield of storage oils in seeds or nutrient tissues and improving the fatty acid profile [42,43]. A transcript encoding nonspecific phospholipase in the black quinoa (gene-LOC110720422) was up-regulated compared to the yellow genotype. This enzyme hydrolyzes phospholipids and galactolipids into triacylglycerol, and nonspecific phospholipase C6 positively regulates seed oil content and oil production in Arabidopsis thaliana [44] (Table S6).

### 3.7. Differential Expression of Amino Acid Biosynthesis and Regulatory Genes in Four Quinoa Cultivars

Compared to the white genotype, the black quinoa encoded 18 transcripts of cysteine protease, with 10 up-regulated and 8 down-regulated. These play crucial roles in hydrolyzing storage proteins and processing precursors of these proteins and inactive forms of other proteases during seed germination. They participate in the maturation of various proteins, the degradation of storage proteins in germinating seeds, and are responsible for the elimination of endogenous proteins [45]. In the red quinoa, compared to the yellow genotype, a transcript encoding S-adenosylmethionine decarboxylase (gene-LOC110686006) was up-regulated. This enzyme is key for polyamine synthesis, catalyzing the formation of S-adenosyl-1-methylthio-3-propylamine (decarboxylS-adenosylmethionine) and serving as an aminopropyl donor [46]. In the black quinoa, two transcripts (gene-LOC110732957 and gene-LOC110734214) encoding S-adenosylmethionine synthase were down-regulated compared to the white genotype, playing significant roles in the synthesis of S-adenosylmethionine synthase. Arabidopsis thaliana has four isozymes of S-adenosylmethionine synthase: MAT1, At MAT2, AtMAT4, and AtMAT3. MAT1, At MAT2, and AtMAT4 are expressed in all plant organs, while AtMAT3 is primarily expressed in pollen [47]. In the black quinoa, compared to the white genotype, there were 13 transcripts encoding serine carboxypeptidase, with 3 up-regulated and 10 down-regulated. Serine carboxypeptidase promotes functional protein maturation. Rice SCP46 is primarily expressed in developing seeds, especially in the embryo, endosperm, and aleurone layer, playing a crucial role in protein transportation, targeting, and processing [48]. Interestingly, a transcript encoding glutamate synthase (gene-LOC110708548) was also down-regulated. This enzyme catalyzes the reductive amination of 2-oxoglutarate, playing a significant role in protein synthesis. The amino nitrogen of glutamate is incorporated into the carbon skeleton through the sequential reaction of GS and glutamate synthase, serving as the source of aspartic acid and alanine amino formed by the transamination of oxaloacetic acid and pyruvate, respectively [49,50]. A transcript encoding glutamine synthase (gene-LOC110722428) was down-regulated. This enzyme catalyzes the conversion of glutamic acid and ammonium NH4^+^ into glutamine, and is the first amino acid synthesized in plant nitrogen assimilation [51,52]. A transcript encoding arginine decarboxylase (gene-LOC110718863) was down-regulated. This enzyme converts arginine into agmatine and catalyzes the first step of arginine biosynthesis of polyamines [53]. One transcript encoding tryptophan synthase was up-regulated (gene-LOC110723779) and the other was down-regulated (gene-LOC110687330). The β subunit of tryptophan synthase catalyzes the last step of tryptophan biosynthesis, the condensation of indole and serine to produce tryptophan. Arabidopsis thaliana has three tryptophan synthase type genes: AtTSB 1 (At5g54810), AtTSB 2 (At4g27070), and AtTSB3 (At5g28237), with AtTSB1 being the main subtype expressed in vegetative tissues [54]. Three transcripts encoding alanine-glyoxylate aminotransferase, one down-regulated (gene-LOC110698132) and two up-regulated (gene-LOC110698132 and gene-LOC110712582), catalyze the reversible conversion of alanine and 2-oxoglutarate into pyruvate and glutamate [55] (Table S7).

**Figure 7:**
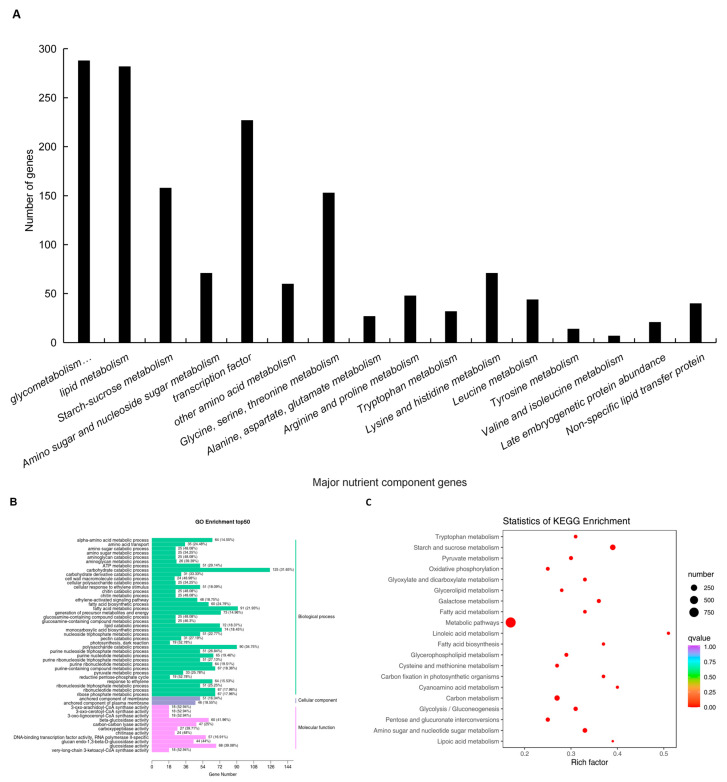
(**A**–**C**) Gene related to nutrient composition; GO enrichment bar graph; bubble diagram of KEGG enrichment.

The differential expression of genes regulating embryo size was also analyzed. Compared to the black quinoa, the white quinoa encoded 20 protein-rich transcripts during late embryogenesis, which are essential for normal seed development and can mitigate water loss during embryo maturation [56,57]. The non-specific lipid transfer protein (nsLTP) is highly expressed in the endosperm of germinating seeds and plays a role in endosperm lipid circulation. There were 30 up-regulated transcripts across the quinoa varieties [58] (Table S8). Additionally, some encoded transcription factors were present in the transcriptome list. Their closest homologs, which have well-established functions in Arabidopsis thaliana, were not highly expressed in the seed tissues (Figure 7A, Table S9). The results of the Gene Ontology (GO) enrichment analysis (Figure 7B) revealed that differentially expressed genes were significantly enriched in the annotated pathways related to “fatty acid biosynthesis”, “amino acid metabolism”, “amino sugar metabolism”, and “lipid catabolism process”. The Kyoto Encyclopedia of Genes and Genomes (KEGG) enrichment analysis (Figure 7C) showed that nutrient-related genes were significantly enriched in the pathways related to linoleic acid metabolism, starch and sucrose metabolism, fatty acid metabolism, galactose metabolism, and tryptophan metabolism. These results align with the pathway analysis of differentially expressed genes, further substantiating that these metabolic pathways are intimately linked to nutrient regulation.

### 3.8. Quantitative Reverse Transcription Polymerase Chain Reaction (qRT-PCR)

**Figure 8:**
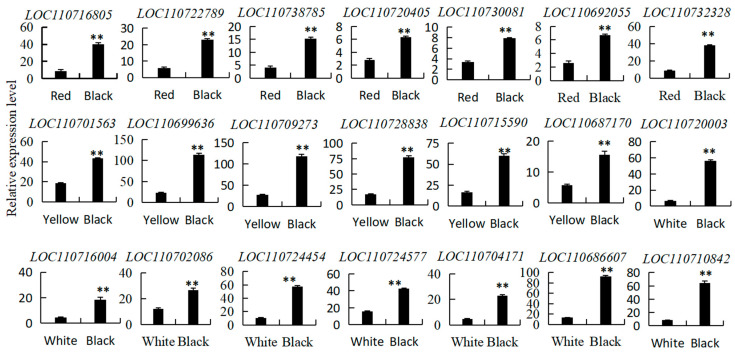
Pattern verification of differentially expressed genes in quinoa seeds with different colors. Independent *t*-tests revealed that the expression levels of different color quinoa seeds exhibited highly significant differences (**, *p* < 0.01).

To assess the quality of the RNA-seq data and validate the differential expression of the genes, the expression levels of 21 genes associated with nutrient synthesis were determined using qRT-PCR. These genes included LOC110716805 encoding beta-galactosidase, LOC110722789 and LOC110732328 encoding granule-bound starch synthase, LOC110738785 encoding alpha-amylase, LOC110720405 encoding ribulose diphosphate carboxylase, LOC110730081 encoding ribulose phosphate kinase, and LOC110692055 encoding beta-amylase. Other genes included LOC110701563, LOC110699636, LOC110709273, LOC110715590, and LOC110728838 encoding GDSL esterase; LOC110710842 and LOC110724454 encoding glutathione transferase; LOC110720003 and LOC110704171 encoding serine/threonine protein kinase; LOC110687170 encoding cysteine receptor-like protein kinase; LOC110716004 and LOC110702086 encoding serine carboxypeptidase; LOC110724577 encoding histidine kinase; and LOC110686607 encoding lysine-specific demethylase. The qRT-PCR results align with the RNA-seq data, indicating the reliability of the transcriptome sequencing results (Figure 8).

## 4. Discussion

Quinoa, frequently referred to as a “pseudo-cereal”, is renowned for its equitable distribution of protein, lipids, and sugars, as well as its superior content of essential amino acids when compared to other grains. Despite this, the underlying regulatory processes governing the nutritional composition of quinoa seeds have not been extensively investigated. Our research contributes to a deeper comprehension of the nutrient profiles in quinoa seeds of varying colors and sheds light on the impact of key genetic traits on these nutrients. This insight provides a solid theoretical basis and technical assistance for the biological breeding of quinoa. 

Our study revealed that, as quinoa color deepens, protein content increases. The lipid contents of the yellow, red, and black quinoa seeds decreased with a deepening color, with black quinoa seeds having the lowest content at 2.7 g/100 g. The metabolomic analysis showed that differential metabolites are primarily related to “sphingo-lipid metabolism”, “phenylalanine metabolism”, and “pentose phosphate pathway”. In essence, seeds of different quinoa varieties are rich in nutrients. Previous studies have suggested that the variation in nutritional components in quinoa is primarily due to genetic diversity rather than environmental factors, with mature quinoa seeds having the highest protein and oil content and the lowest starch content [59,60]. 

This differs from our findings, possibly due to varietal differences. The analysis of seed metabolites from differently colored quinoa varieties revealed significant differences among varieties of the same color in the same production area [61]. We found that the four quinoa genotypes had varying seed protein contents towards maturity [62], and our analysis of nutrient accumulation in developing seeds showed that they had different levels of storage compounds, starch, and oil. In Arabidopsis thaliana, starch briefly accumulates during early embryogenesis and then serves as a raw material for oil synthesis along with carbon. At maturity, the dry weight of the seeds remains constant, implying that the contents of stored oil and protein remain unchanged, while water loss leads to a decrease in the synthesis of storage compounds and the content of sucrose, stachyose, and raffinose, resulting in a high oil content and low starch content in mature seeds [63]. Black quinoa, like white, yellow, and red varieties, had the highest starch content (54.1 g/100 g), followed by protein content (16.1 g/100 g) and lipid content (2.7 g/100 g) at maturity. This may suggest a negative correlation between protein and starch content and lipid content in quinoa, but a larger sample of genotypes is needed to confirm this hypothesis. The amino acid composition of the mature seeds was similar to previously reported data, showing only minor genotypic differences [64].

Understanding the specific seed tissues that store compounds in seed nutrients may explain the differences observed between crops and even between different varieties of the same crop. The gene LOC110721444 (homologous gene AT1G30120), which encodes pyruvate dehydrogenase, plays a crucial role in fatty acid synthesis. The co-expression of pyruvate dehydrogenase and fatty acid biosynthesis suggests the primary flux direction of acetyl coenzyme A [65]. The pyruvate kinase gene LOC110732515 (homologous gene AT1G32440) exists in cytoplasmic and cytosomal subtypes. It catalyzes the ADP-dependent transformation of phosphoenolpyruvate into pyruvate while producing ATP. Pyruvate is a control point of glycolysis, and its activity directly impacts both ATP and ADP. A decrease in PK activity, an inhibitor of seed phosphofructokinase, will lead to the inhibition of glycolysis flux and the accumulation of carbohydrate precursors. Moreover, Arabidopsis plastid pyruvate kinase is closely related to lipid biosynthesis and compound accumulation, and is considered to be a potential regulator of oil synthesis in oilseeds [66]. We hypothesize that pyruvate kinase in quinoa plays a significant role in promoting lipid biosynthesis and increasing seed oil content.

Sugar transporters play crucial roles in the carbon output of Arabidopsis thaliana [67]. The gene LOC110687202 (homologous gene AT1G11260) encodes a high-affinity sugar transporter in Arabidopsis thaliana, which is highly down-regulated and can transport a variety of hexose [68]. The sugar transporter encoded in quinoa is up-regulated, which is contrary to Arabidopsis thaliana, likely due to the high content of other ion transporters in quinoa seeds. In the late stage of Arabidopsis seed development, stearoyl desaturase and fatty acid carrier protein participate in the formation of the embryonic stratum corneum, promoting the production of stored lipids in the mature stage. The fatty acid desaturase encoded by gene LOC110738720 (homologous gene AT5G16240) shows the highest expression level and is up-regulated in all seed tissues. The gene encoded by quinoa is also up-regulated. The contents of oil and oleic acid in mature seeds of Arabidopsis thaliana decrease, while the content of stearic acid increase significantly, promoting the biosynthesis of seed oil and oleic acid in embryos [69,70,71].

Phospholipase is a key component of the phospholipid signaling network that regulates plant growth and development [72]. It participates in phospholipid degradation, resulting in phosphatidic acid and diacylglycerol, and galactose synthesis. The transcription gene LOC110720422 (homologous gene AT3G03540) encoding phospholipase is up-regulated in Arabidopsis seedlings and roots, and most genes in the PLC family of phospholipase are over-expressed in leaves [73]. The transcription encoded by seeds in quinoa is also up-regulated. We hypothesize that phospholipase plays a significant role in galactose synthesis in quinoa.

Most of the protein and oil accumulated in quinoa seeds are deposited in embryonic tissues, while starch is accumulated in the endosperm, which is also true of cereals [5]. Compounds co-accumulate in embryonic tissue, so genotypes with a high protein content also have the highest oil content (black quinoa), indicating that quinoa genotypes have differences in seed distribution to different storage substances. In conclusion, our data reveal significant differences in the expression levels among the white, yellow, red, and black varieties of quinoa seed transcripts, which encode transcription factors and functions in sugar, starch, lipid, and protein biosynthetic pathways. Grain endosperm is the main source of starch storage [74], and starch is also the main storage compound of quinoa seeds, which is primarily stored in the outer endosperm. Therefore, significant changes in the expression of genes controlling the starch synthesis in seeds may impact the seed content. Compared with the black quinoa genotype, transcripts encoding granule-bound starch synthase and starch-degrading enzyme (β-amylase) were highly up-regulated in red quinoa, indicating that starch may be in turnover. In addition, in red quinoa, the transcript encoding ribulose diphosphate was up-regulated, and this protein plays a central role in the Calvin cycle during photosynthesis [75]. The protein content in the seeds of different plant species varies significantly. Among common crops, soybeans have the highest protein content at approximately 45% [76]. Proteins in soybeans and quinoa are primarily stored in the embryos. Oil is also a crucial storage compound, and its synthesis and accumulation regulation in plants are complex [77]. In yellow varieties, the transcription of phospholipase, which is involved in lipid metabolism and regulates various plant stress responses, was up-regulated. Another up-regulated transcription encoded an acyltransferase involved in oil synthesis. 

## 5. Conclusions

We identified four genotypes of quinoa seeds and, through metabolome and transcriptome analyses, pinpointed the differential metabolites and genes that govern the nutritional composition of these seeds. The genes that exhibited differential expression were found to be predominantly involved in key pathways such as “linoleic acid metabolism”, “unsaturated fatty acid biosynthesis”, and “amino acid biosynthesis”. In glycolysis, lipid anabolism, and amino acid bioanabolism, the transcript levels are up-regulated or down-regulated. By tracking metabolomic variations in quinoa grains of different colors, we have opened up avenues for selecting and breeding strains that are enriched in particular nutrients. Furthermore, the analysis of differential metabolites and genes with varying expression levels in quinoa grains has deepened our understanding of the regulatory pathways behind the synthesis of nutritional compounds in quinoa.

## Data Availability

The original contributions presented in the study are included in the article/Supplementary Materials, further inquiries can be directed to the corresponding authors.

## Supplementary Materials

The following supporting information can be downloaded at: https://www.mdpi.com/article/10.3390/foods13091325/s1, Figure S1: (A–D) Total ion current (TIC) of QC samples in positive ion mode (A) and negative ion mode (B), the horizontal axis represents the retention time (Rt) for metabolite detection, while the vertical axis denotes the ion current intensity measured in counts per second (cps). (C) Pearson correlation coefficients of black, red, white and yellow quality control samples, the vertical and diagonal lines on the graph indicate the sample names for various samples, with different colors signifying different Pearson correlation coefficients. A deeper shade of red indicates a stronger positive correlation, while a prevalence of green suggests a weaker correlation. Conversely, a richer blue hue denotes a stronger negative correlation. Additionally, the correlation coefficients between pairs of samples are displayed within the grid. The “correlation expt” denotes the repeated correlation assessments conducted on the experimental test samples, whereas “correlation mix” refers to the repeated correlation evaluations carried out on the quality control (QC) samples. Classification and proportion of metabolites of different quinoa seeds (D). Figure S2: (A) OPLS-DA verification diagrams, the horizontal axis represents the accuracy of the model, while the vertical axis represents the frequency of the model’s classification effect. Figure S2: (B–D) Thermogram of metabolites, from left to right: lipids, proteins, and starch, horizontal axis represents metabolic groups, while the vertical axis denotes different metabolites. Figure S2: (E) Contrast diagrams illustrating comparisons between white and yellow, white and red, white and black, yellow and red, yellow and black, as well as red and black, the horizontal axis represents the log2 fold change (FC) of differentially expressed metabolites, which is the logarithm to the base 2 of the ratio of change in these metabolites. The vertical axis indicates the differentially expressed metabolites. Red denotes upregulated metabolites, while green signifies downregulated metabolites. Figure S2: (F) GO enrichment diagram between different contrast groups, the horizontal axis represents the proportion of metabolites annotated to a given entry relative to the total number of annotated metabolites, while the vertical axis denotes the name of the GO (Gene Ontology) term. Figure S3: (A,B) Figure A presents the correlation heatmap of 12 quinoa samples based on transcriptomic data, where the square of the Pearson correlation coefficient (R2) between biological replicates is expected to be above 0.8. Figure B depicts the gene clustering thermogram, with the abscissa indicating sample names and their hierarchical clustering outcomes, and the ordinate showing differentially expressed genes along with their hierarchical clustering results. Figure S4: (A) The differential gene volcano plot features the log2 fold change of gene expression on the horizontal axis and the significance level of differential genes on the vertical axis. Red dots signify upregulated genes, while green dots indicate downregulated genes. Figure S4 (B) The enrichment scatter plot depicts KEGG pathways on the vertical axis and the Rich Factor on the horizontal axis. A higher Rich Factor indicates a greater degree of enrichment. The size of the dot corresponds to the number of differentially expressed genes enriched in the pathway; larger dots signify more genes. The color of the dot increasingly trending towards red indicates more significant enrichment. Figure S4 (C) The differential gene category bar chart presents secondary GO (Gene Ontology) terms on the horizontal axis and the number of differential genes associated with each GO term on the vertical axis. Figure S5: Nine quadrant diagram of correlation analysis, the correlation analysis nine-quadrant plot showcases the log2 fold change (FC) of genes on the horizontal axis and the log2 FC of metabolites on the vertical axis. Table S1: Primers used in the study. Table S2: Types and Relative Contents of Quinoa Metabolites. Table S3: Statistics on the Number of Differential Metabolites. Table S4: Quinoa Transcriptome Sequencing Quality Statistics. Table S5: Statistics of Differentially Expressed Genes. Table S6: Carbon Metabolism and Differential Expression of Regulatory Genes. Table S7: Differential expression of lipid anabolism and regulatory genes. Table S8: Biosynthesis of amino acids and differential expression of regulatory genes. Table S9: Differential Expression of Embryonic Size Regulating Genes.
