# Supplementary material for: Metabolome and Transcriptome Association Analysis Reveals Mechanism of Synthesis of Nutrient Composition in Quinoa (Chenopodium quinoa Willd.) Seeds
Source: Foods. 2024 Apr 26;13(9):1325. doi: 10.3390/foods13091325 (PMC11082971; doi:10.3390/foods13091325)
Supplement: Supplementary file 1 [file foods-13-01325-s001.zip › manuscript.pptx]

## Slide 1
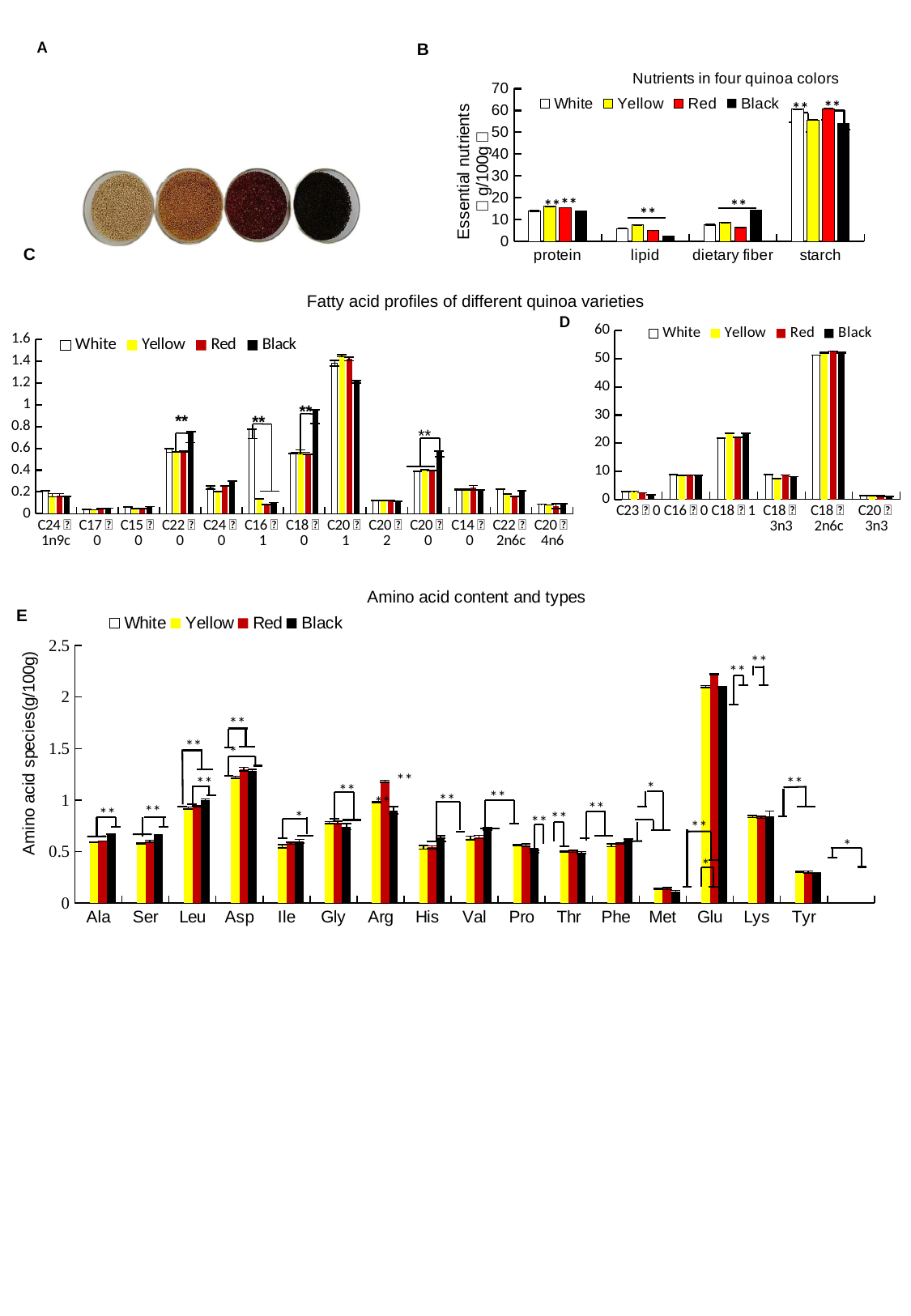

A
B
### Chart: Nutrients in four quinoa colors
| Category | White | Yellow | Red | Black |
|---|---|---|---|---|
| protein | 13.83 | 16.1 | 15.5 | 14.3 |
| lipid | 5.9 | 7.5 | 5.03 | 2.73 |
| dietary fiber | 7.63 | 8.57 | 6.42 | 14.57 |
| starch | 60.57 | 55.7 | 60.9 | 54.07 |
**
**
**
**
**
**
C
Fatty acid profiles of different quinoa varieties
D
### Chart
| Category | White | Yellow | Red | Black |
|---|---|---|---|---|
| C23：0 | 2.83 | 2.92 | 2.42 | 1.63 |
| C16：0 | 8.88 | 8.61 | 8.71 | 8.63 |
| C18：1 | 21.8 | 23.4 | 22.0 | 23.6 |
| C18：3n3 | 8.83 | 7.36 | 8.73 | 8.14 |
| C18：2n6c | 51.2 | 52.1 | 52.7 | 52.1 |
| C20：3n3 | 1.44 | 1.44 | 1.2 | 1.1 |
### Chart
| Category | White | Yellow | Red | Black |
|---|---|---|---|---|
| C24：1n9c | 0.208 | 0.17 | 0.17 | 0.158 |
| C17：0 | 0.0394 | 0.0358 | 0.046 | 0.0527 |
| C15：0 | 0.0627 | 0.0468 | 0.0489 | 0.0668 |
| C22：0 | 0.596 | 0.568 | 0.572 | 0.756 |
| C24：0 | 0.24 | 0.202 | 0.258 | 0.301 |
| C16：1 | 0.774 | 0.138 | 0.0832 | 0.103 |
| C18：0 | 0.554 | 0.568 | 0.544 | 0.952 |
| C20：1 | 1.38 | 1.45 | 1.42 | 1.21 |
| C20：2 | 0.125 | 0.122 | 0.124 | 0.114 |
| C20：0 | 0.391 | 0.404 | 0.399 | 0.574 |
| C14：0 | 0.221 | 0.221 | 0.24 | 0.216 |
| C22：2n6c | 0.226 | 0.182 | 0.158 | 0.208 |
| C20：4n6 | 0.0871 | 0.0816 | 0.07 | 0.0918 |**
**
**
**
### Chart: Amino acid content and types
| Category | White | Yellow | Red | Black |
|---|---|---|---|---|
| Ala | 0.57 | 0.59 | 0.6 | 0.67 |
| Ser | 0.58 | 0.58 | 0.6 | 0.65 |
| Leu | 0.89 | 0.92 | 0.94 | 1.0 |
| Asp | 1.2 | 1.22 | 1.3 | 1.28 |
| Ile | 0.54 | 0.55 | 0.58 | 0.6 |
| Gly | 0.72 | 0.78 | 0.78 | 0.74 |
| Arg | 0.86 | 0.98 | 1.18 | 0.9 |
| His | 0.5 | 0.54 | 0.54 | 0.64 |
| Val | 0.63 | 0.63 | 0.64 | 0.72 |
| Pro | 0.41 | 0.56 | 0.56 | 0.5 |
| Thr | 0.43 | 0.5 | 0.5 | 0.49 |
| Phe | 0.54 | 0.56 | 0.58 | 0.61 |
| Met | 0.15 | 0.14 | 0.14 | 0.11 |
| Glu | 1.87 | 2.1 | 2.22 | 2.09 |
| Lys | 0.78 | 0.84 | 0.83 | 0.84 |
| Tyr | 0.31 | 0.3 | 0.3 | 0.28 |E
E
**
**
**
**
*
**
**
**
**
*
**
**
**
**
**
**
**
*
**
**
**
*
*
**

## Slide 2
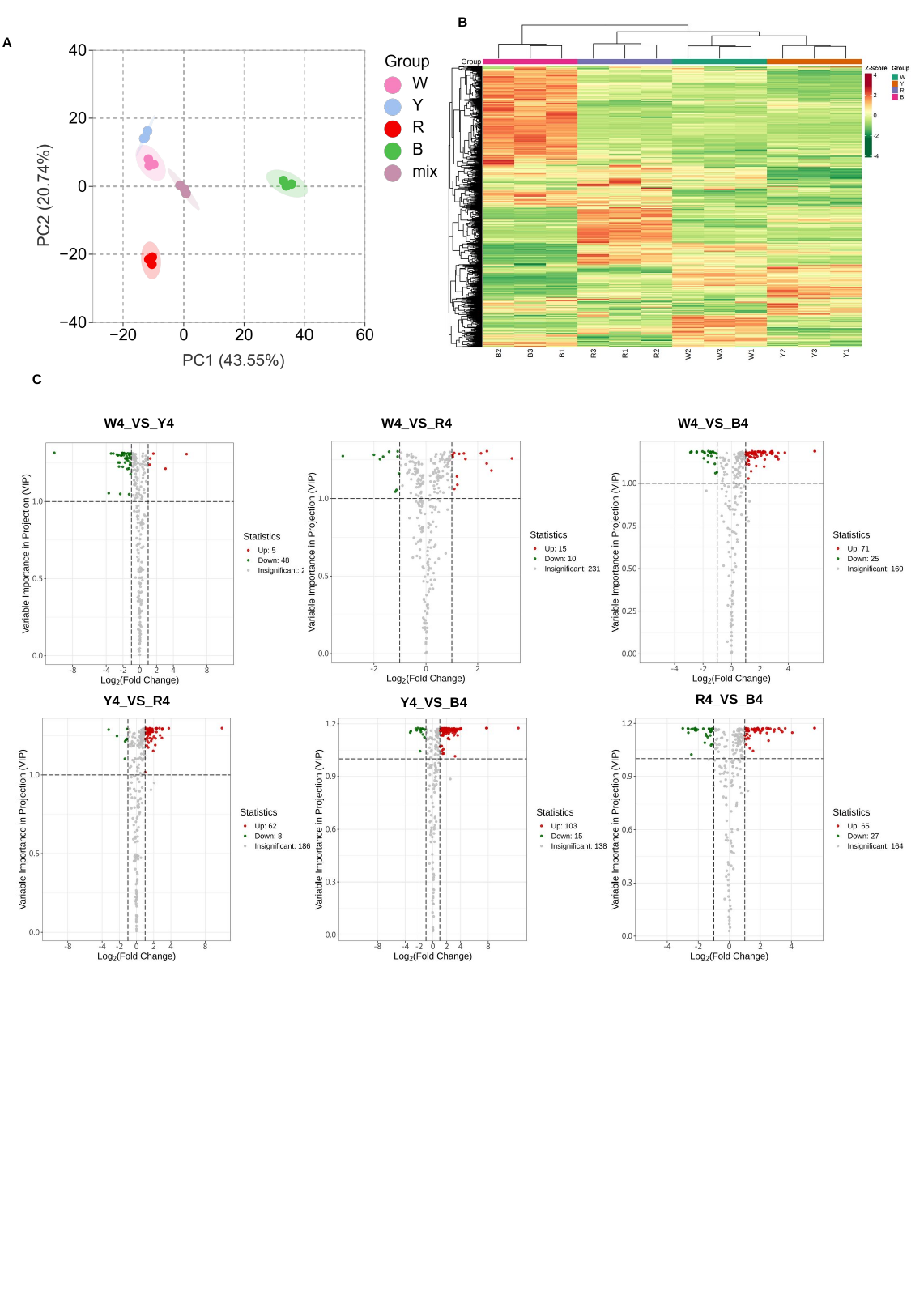

B
A
C
W4_VS_R4
W4_VS_B4
W4_VS_Y4
Y4_VS_R4
Y4_VS_B4
R4_VS_B4

## Slide 3
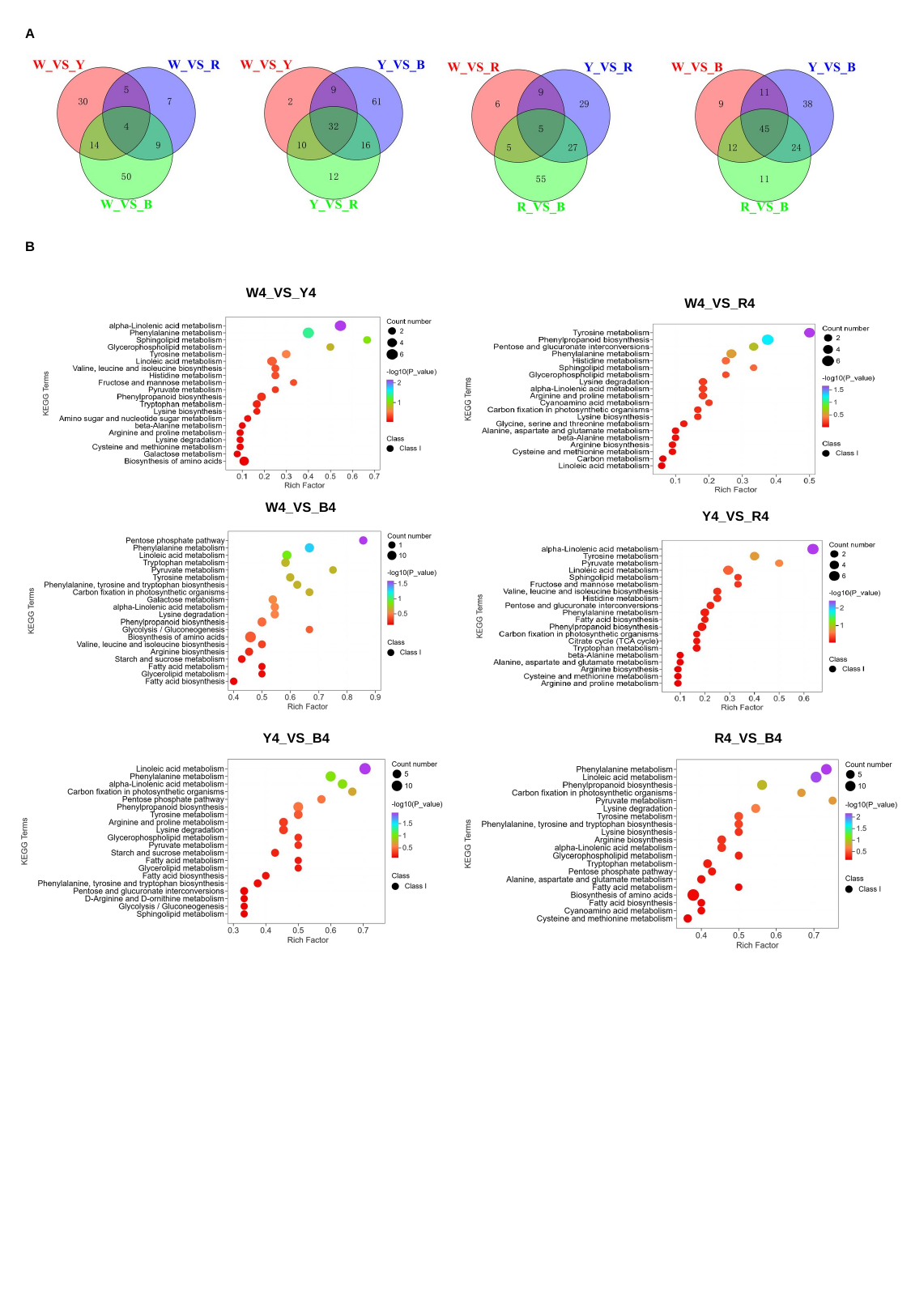

A
B
W4_VS_Y4
W4_VS_R4
W4_VS_B4
Y4_VS_R4
Y4_VS_B4
R4_VS_B4

## Slide 4
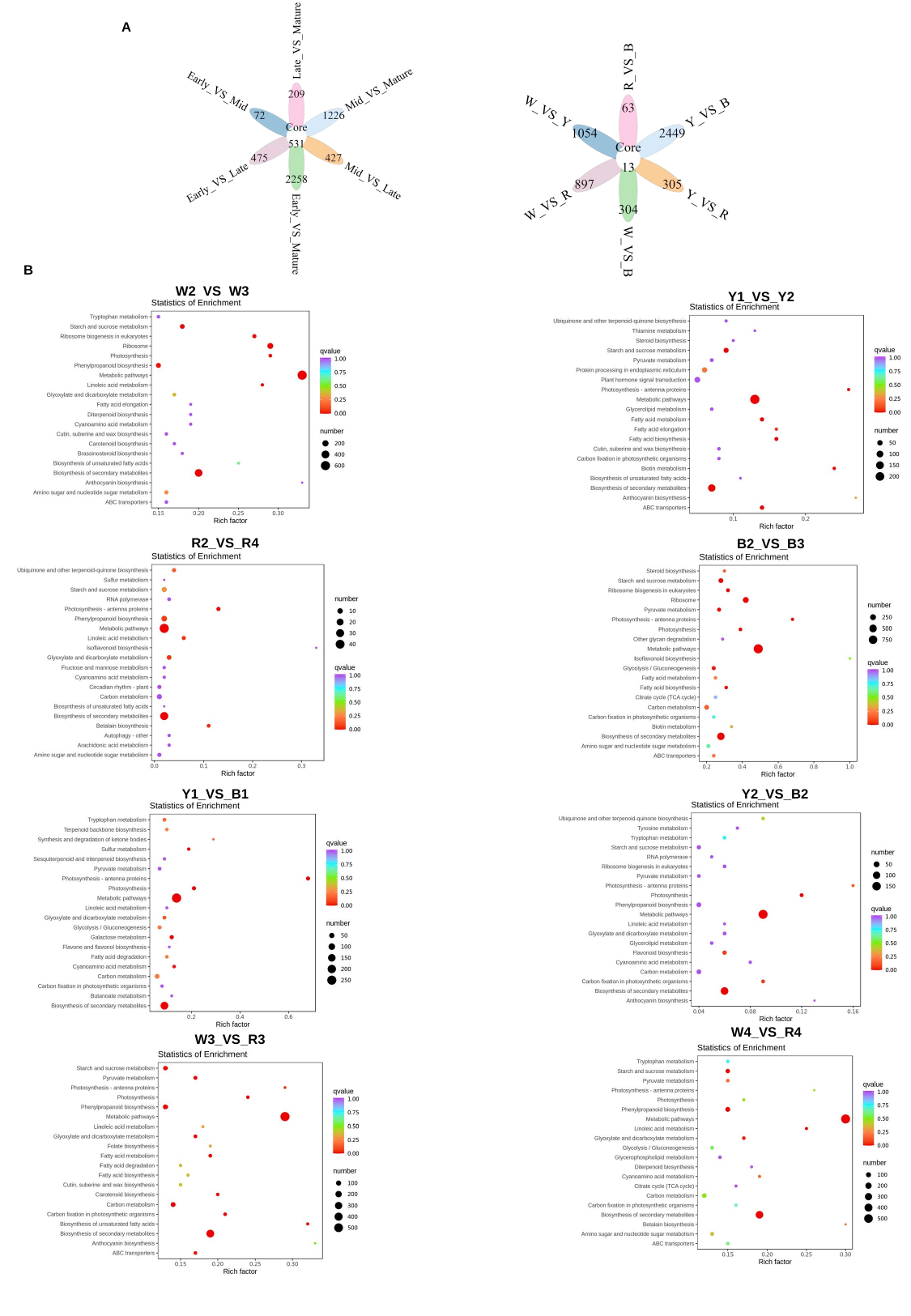

A
B
W2_VS_W3
Y1_VS_Y2
R2_VS_R4
B2_VS_B3
Y1_VS_B1
Y2_VS_B2
W4_VS_R4
W3_VS_R3

## Slide 5
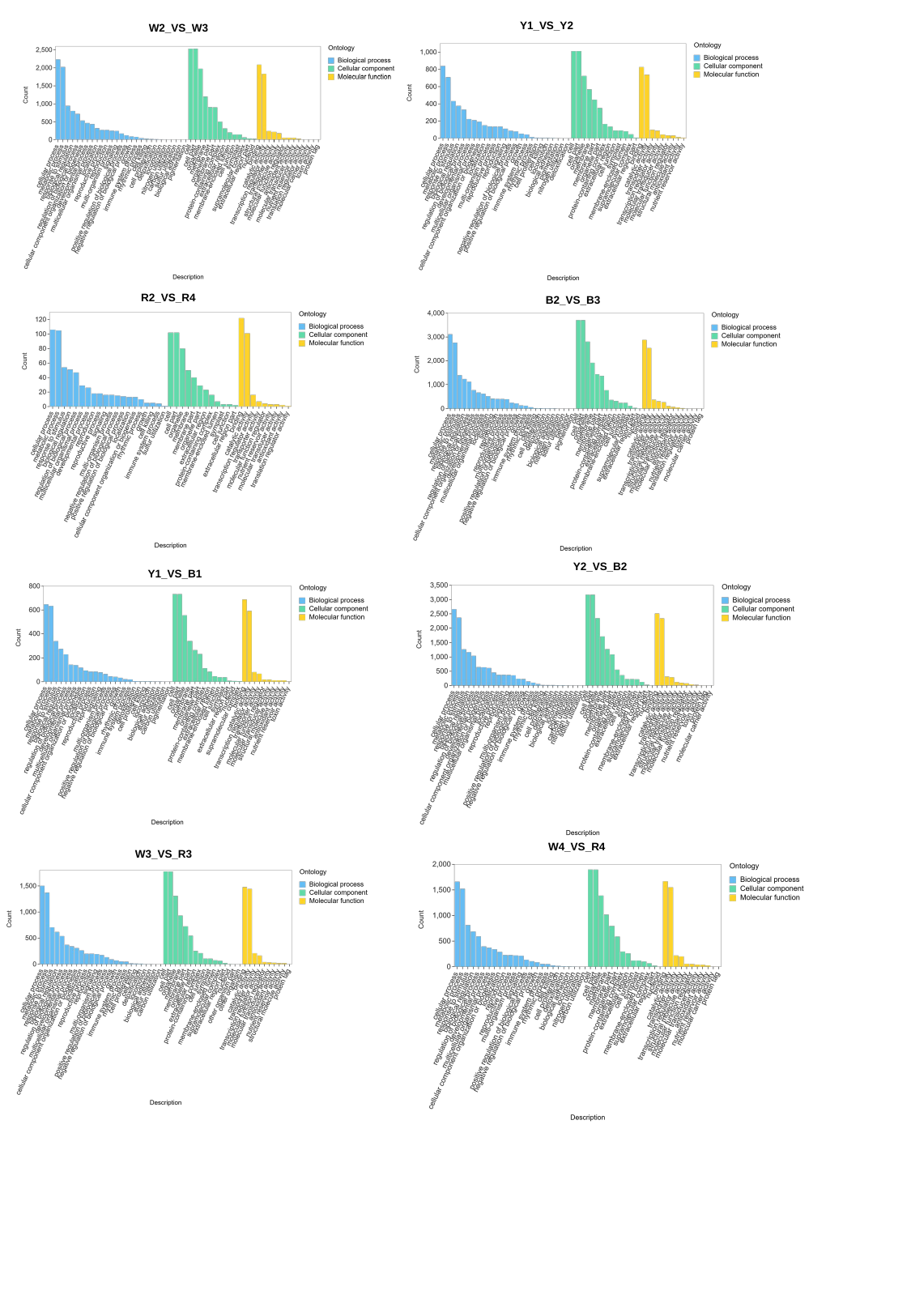

Y1_VS_Y2
W2_VS_W3
R2_VS_R4
B2_VS_B3
Y2_VS_B2
Y1_VS_B1
W4_VS_R4
W3_VS_R3

## Slide 6
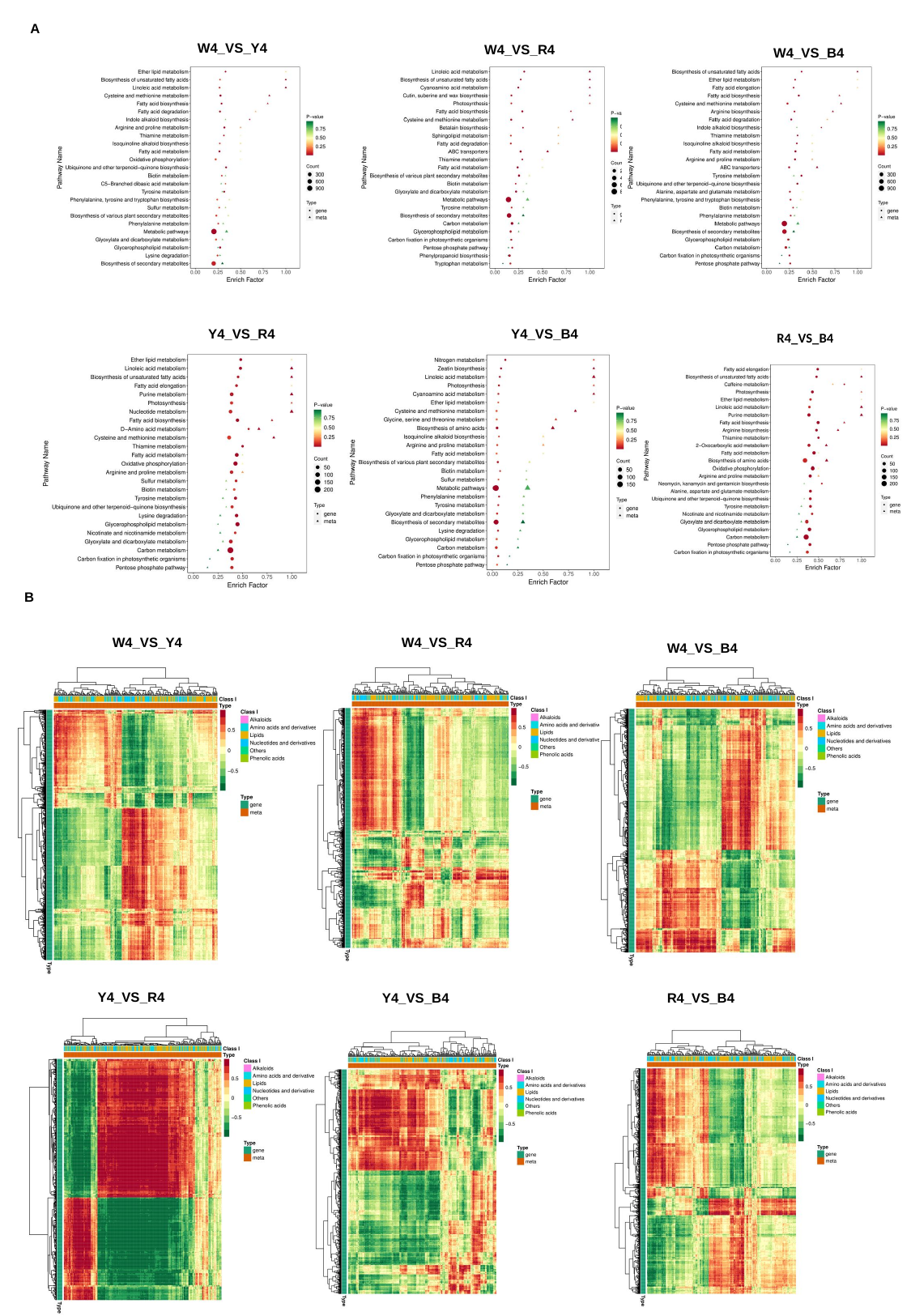

A
W4_VS_Y4
W4_VS_R4
W4_VS_B4
Y4_VS_B4
R4_VS_B4
Y4_VS_R4
B
W4_VS_Y4
W4_VS_R4
W4_VS_B4
Y4_VS_R4
R4_VS_B4
Y4_VS_B4

## Slide 7
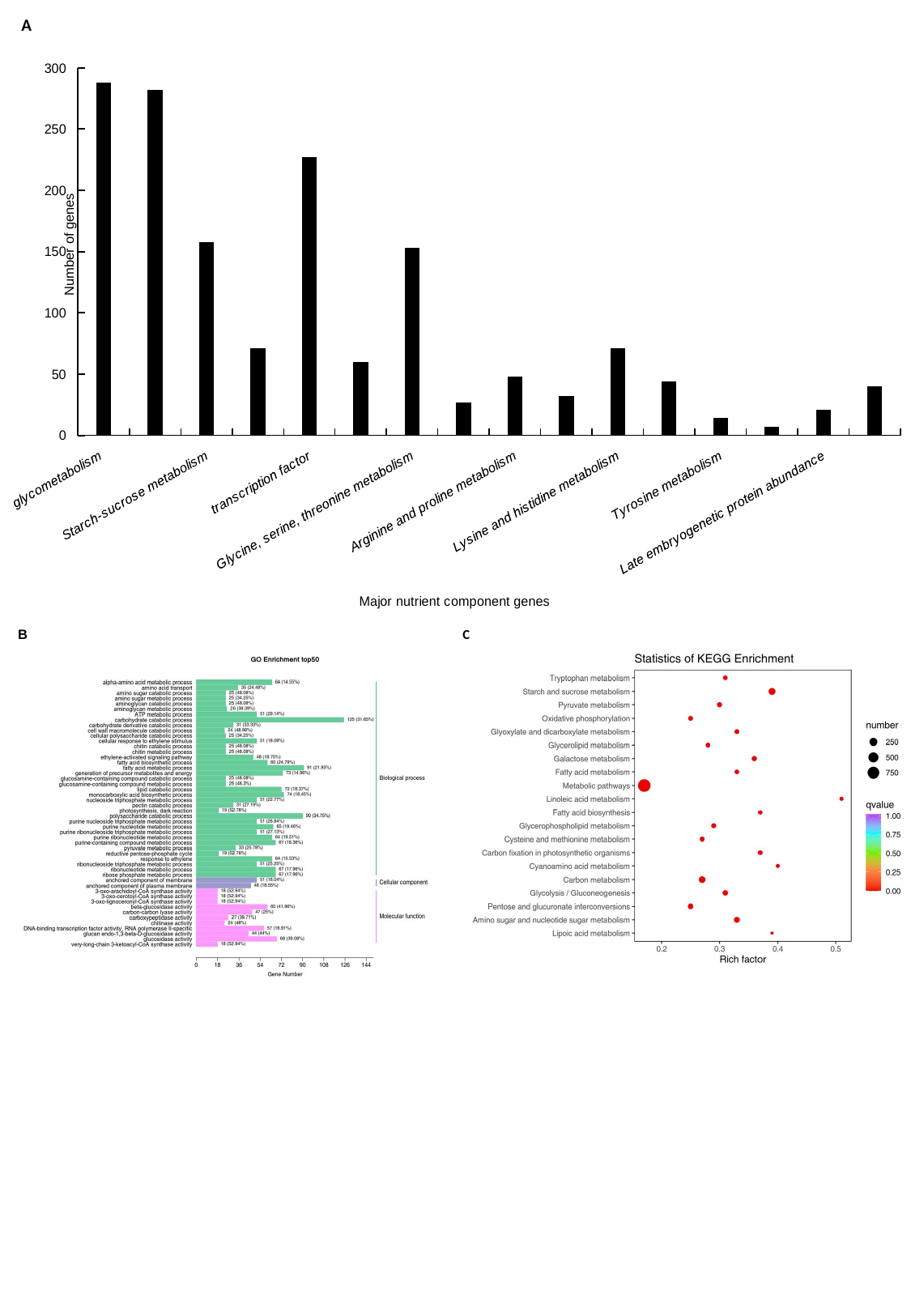

A
### Chart
| Category | Number of genes |
|---|---|
| glycometabolism
 | 288.0 |
| lipid metabolism | 282.0 |
| Starch-sucrose metabolism | 158.0 |
| Amino sugar and nucleoside sugar metabolism | 71.0 |
| transcription factor | 227.0 |
| other amino acid metabolism | 60.0 |
| Glycine, serine, threonine metabolism | 153.0 |
| Alanine, aspartate, glutamate metabolism | 27.0 |
| Arginine and proline metabolism | 48.0 |
| Tryptophan metabolism | 32.0 |
| Lysine and histidine metabolism | 71.0 |
| Leucine metabolism | 44.0 |
| Tyrosine metabolism | 14.0 |
| Valine and isoleucine metabolism | 7.0 |
| Late embryogenetic protein abundance | 21.0 |
| Non-specific lipid transfer protein | 40.0 |B
B
C
C

## Slide 8
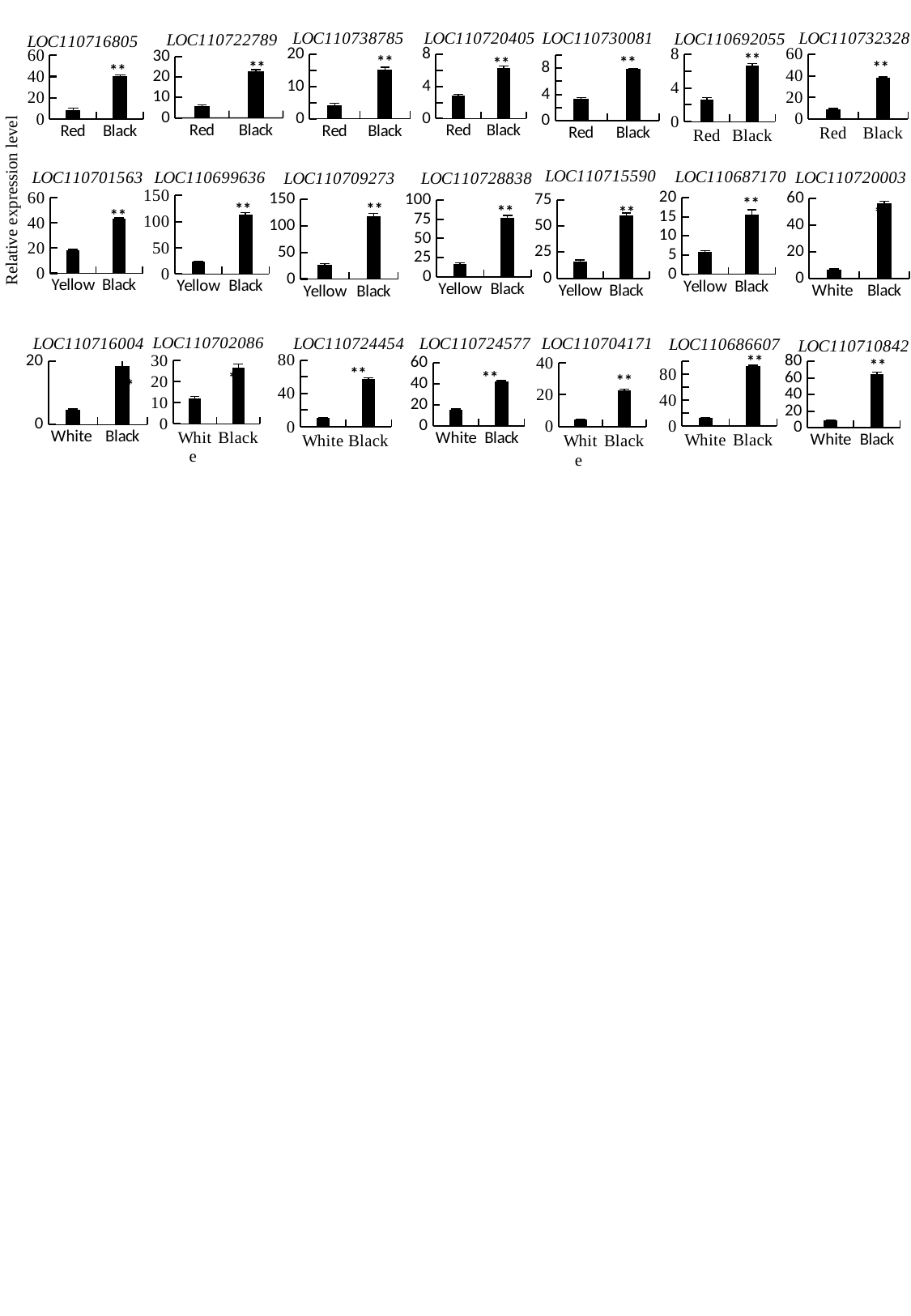

Relative expression level
### Chart: LOC110732328
| Category | |
|---|---|
| Red | 8.97177324766473 |
| Black | 38.1460626114779 | **
### Chart: LOC110730081
| Category | |
|---|---|
| Red | 3.33669022820624 |
| Black | 7.85726875887434 | **
### Chart: LOC110722789
| Category | |
|---|---|
| Red | 5.64176584805611 |
| Black | 22.8658952528683 | **
### Chart: LOC110692055
| Category | |
|---|---|
| Red | 2.60969580977988 |
| Black | 6.68144000750468 | **
### Chart: LOC110720405
| Category | |
|---|---|
| Red | 2.81131610021367 |
| Black | 6.31011961946557 | **
### Chart: LOC110738785
| Category | |
|---|---|
| Red | 4.0557629259409 |
| Black | 15.1815420439778 | **
### Chart: LOC110716805
| Category | |
|---|---|
| Red | 8.84687879238229 |
| Black | 40.0067132009169 | **
### Chart: LOC110728838
| Category | |
|---|---|
| Yellow | 16.7792728414584 |
| Black | 77.2295225338389 |**
### Chart: LOC110715590
| Category | |
|---|---|
| Yellow | 16.3067617098436 |
| Black | 60.2768232206248 | **
### Chart: LOC110687170
| Category | |
|---|---|
| Yellow | 5.73707274171169 |
| Black | 15.5401096801966 |**
### Chart: LOC110720003
| Category | |
|---|---|
| White | 6.28718760034831 |
| Black | 56.4292248232332 | **
### Chart: LOC110709273
| Category | |
|---|---|
| Yellow | 27.1524662879078 |
| Black | 117.950235676904 | **
### Chart: LOC110699636
| Category | |
|---|---|
| Yellow | 22.9390885648575 |
| Black | 114.088823056687 | **
### Chart: LOC110701563
| Category | |
|---|---|
| Yellow | 18.6032484451545 |
| Black | 43.1807434783355 | **
### Chart: LOC110702086
| Category | |
|---|---|
| White | 12.1148348221723 |
| Black | 26.5336072447686 |**
### Chart: LOC110686607
| Category | |
|---|---|
| White | 12.7909124182271 |
| Black | 92.6176512985797 | **
### Chart: LOC110716004
| Category | |
|---|---|
| White | 4.69538762431135 |
| Black | 18.3103449718637 | **
### Chart: LOC110710842
| Category | |
|---|---|
| White | 8.54869218265605 |
| Black | 64.524605318156 | **
### Chart: LOC110724577
| Category | |
|---|---|
| White | 15.5475547891068 |
| Black | 42.5221690656418 | **
### Chart: LOC110704171
| Category | |
|---|---|
| White | 4.61519547580108 |
| Black | 22.8184152678268 | **
### Chart: LOC110724454
| Category | |
|---|---|
| White | 10.6142353755097 |
| Black | 57.6789515558476 | **
