# Supplementary material for: Metabolome and Transcriptome Association Analysis Reveals Mechanism of Synthesis of Nutrient Composition in Quinoa (Chenopodium quinoa Willd.) Seeds
Source: Foods. 2024 Apr 26;13(9):1325. doi: 10.3390/foods13091325 (PMC11082971; doi:10.3390/foods13091325)
Supplement: Supplementary file 1 [file foods-13-01325-s001.zip › Supporting information.pdf]

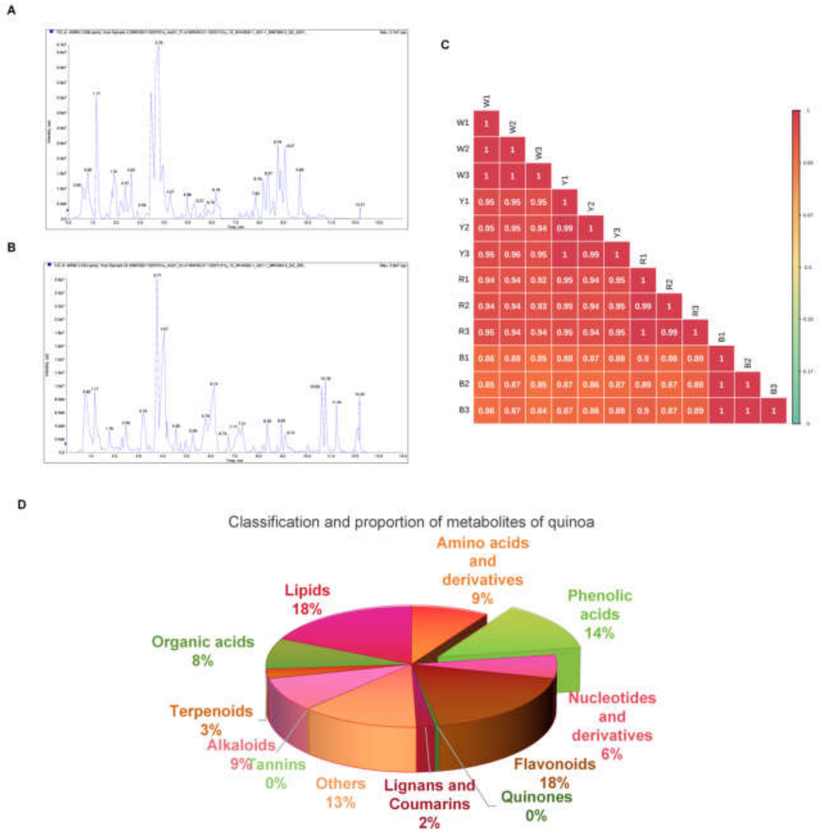

Figure S1: (A - D) (A-D) Total ion current (TIC) of QC samples in positive ion mode (A) and negative ion mode (B), the horizontal axis represents the retention time (Rt) for metabolite detection, while the vertical axis denotes the ion current intensity measured in counts per second (cps). (C)Pearson correlation coefficients of black, red, white and yellow quality control samples, the vertical and diagonal lines on the graph indicate the sample names for various samples, with different colors signifying different Pearson correlation coefficients. A deeper shade of red indicates a stronger positive correlation, while a prevalence of green suggests a weaker correlation. Conversely, a richer blue hue denotes a stronger negative correlation. Additionally, the correlation coefficients between pairs of samples are displayed within the grid. The "correlation_expt" denotes the repeated correlation assessments conducted on the experimental test samples, whereas "correlation_mix" refers to the repeated correlation evaluations carried out on the quality

control (QC) samples. Classification and proportion of metabolites of different quinoa seeds(D).

设置了格式: 字体: 10.5 磅, 字体颜色: 红色

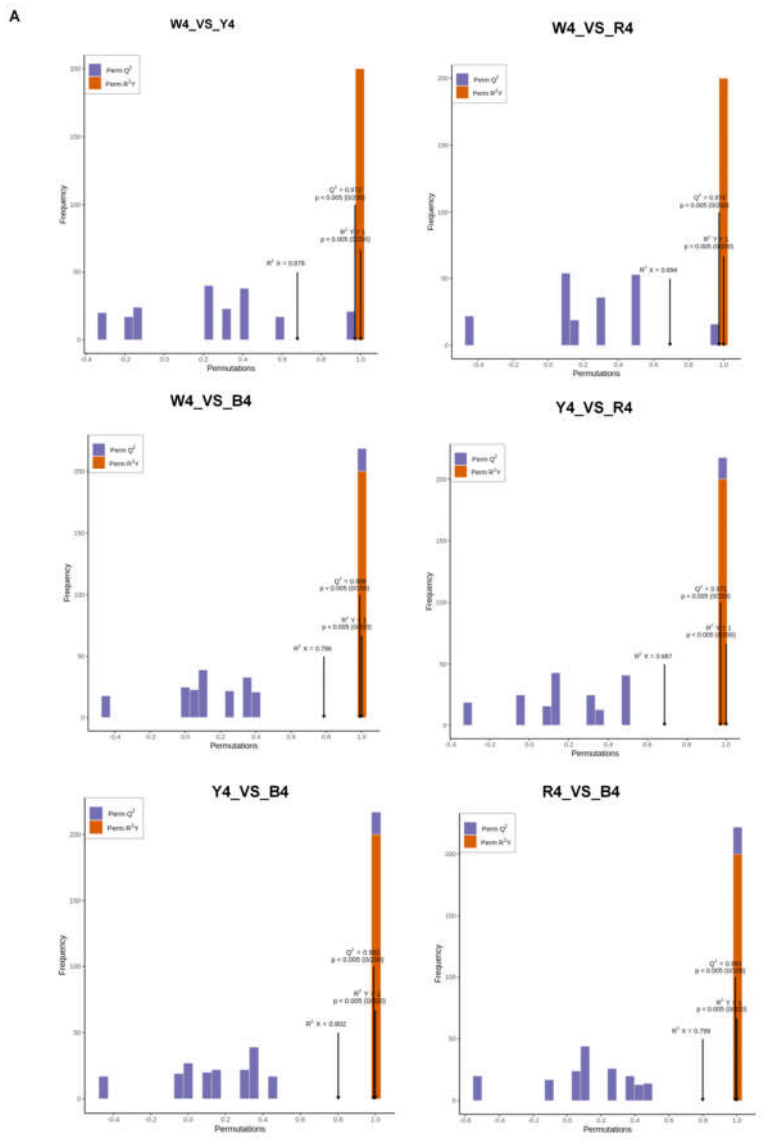

Figure S2: (A) OPLS-DA verification diagrams, the horizontal axis represents the accuracy of the model, while the vertical axis represents the frequency of the model's classification effect.

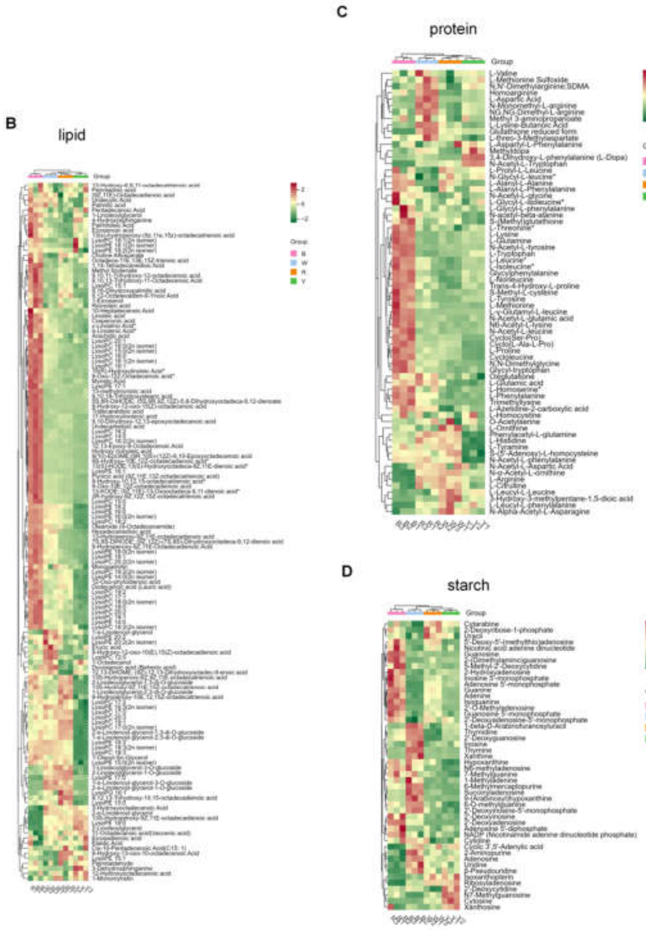

Figure S2: (B - D) Thermogram of metabolites, from left to right: lipids, proteins, and starch, horizontal axis represents metabolic groups, while the vertical axis denotes different metabolites.

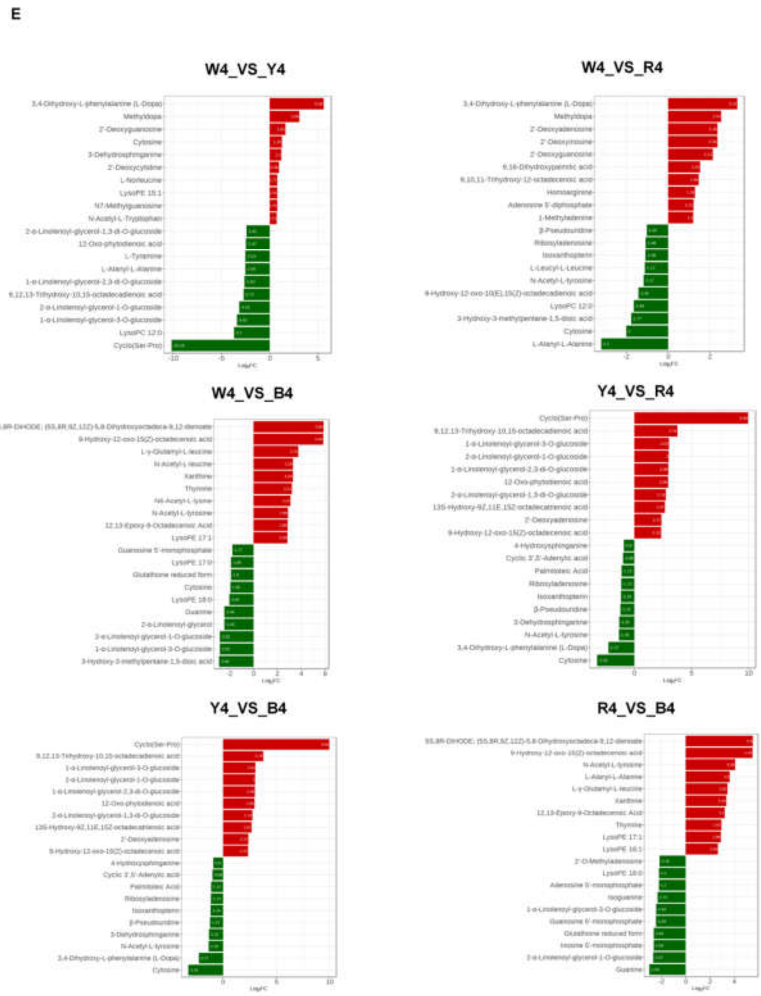

Figure S2: (E) Contrast diagrams illustrating comparisons between white and yellow, white and red, white and black, yellow and red, yellow and black, as well as red and black, the horizontal axis represents the log2 fold change (FC) of differentially expressed metabolites, which is the logarithm to the base 2 of the ratio of change in these metabolites. The vertical axis indicates the differentially expressed metabolites. Red denotes upregulated metabolites, while green signifies downregulated metabolites.

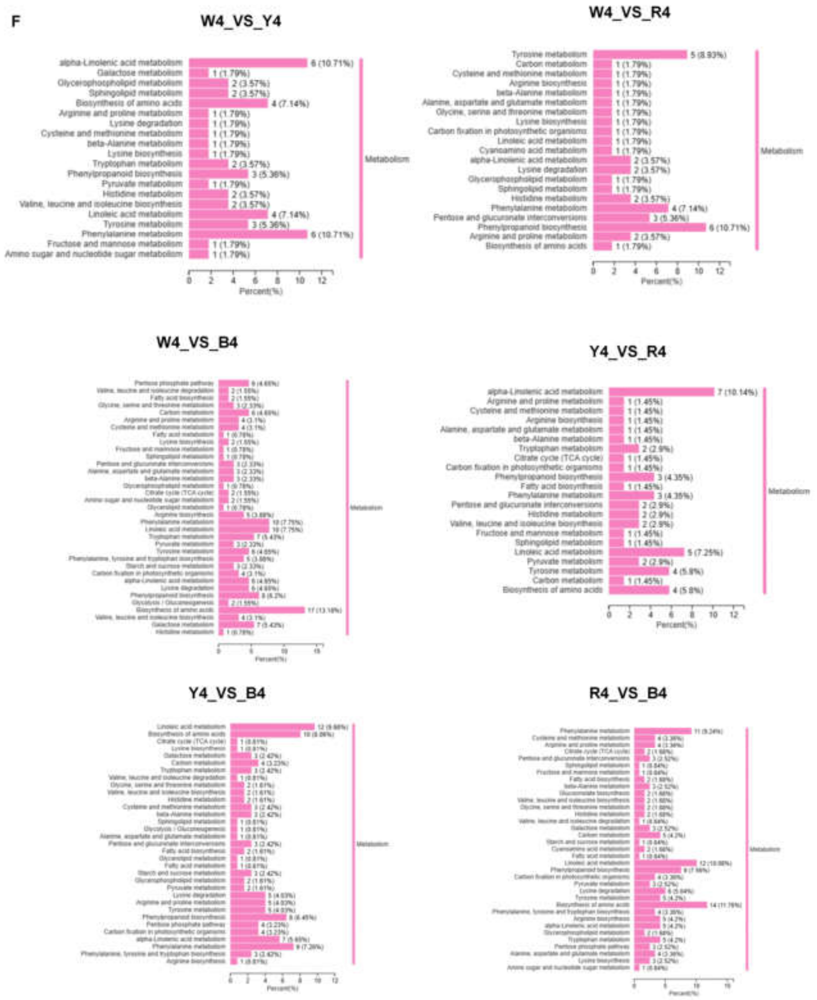

Figure S2: (F) GO enrichment diagram between different contrast groups, the horizontal axis represents the proportion of metabolites annotated to a given entry relative to the total number of annotated metabolites, while the vertical axis denotes the name of the GO (Gene Ontology) term.

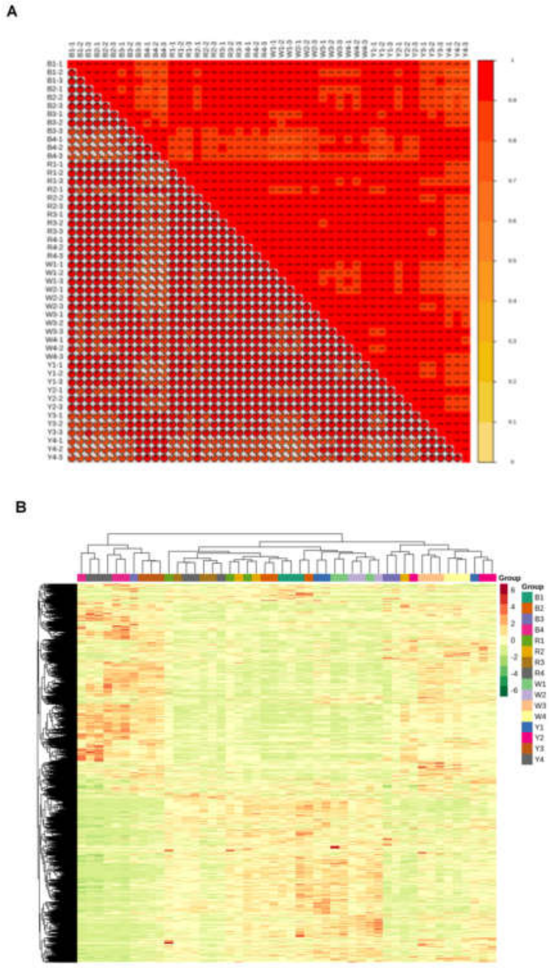

Fig. S3 (A - B) Figure A presents the correlation heatmap of 12 quinoa samples based on transcriptomic data, where the square of the Pearson correlation coefficient (R^2) between biological replicates is expected to be above 0.8. Figure B depicts the gene clustering thermogram, with the abscissa indicating sample names and their hierarchical clustering outcomes, and the ordinate showing differentially expressed genes along with their hierarchical clustering results.

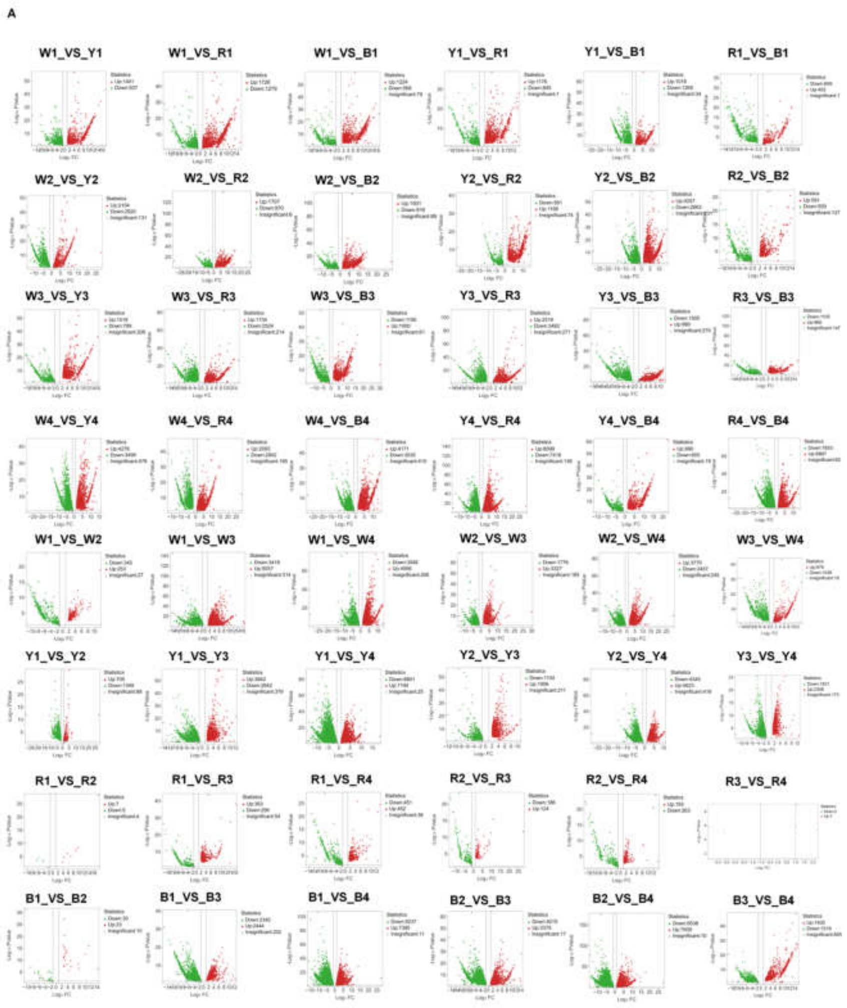

Fig. S4 (A) The differential gene volcano plot features the log2 fold change of gene expression on the horizontal axis and the significance level of differential genes on the vertical axis. Red dots signify upregulated genes, while green dots indicate downregulated genes.

B-1

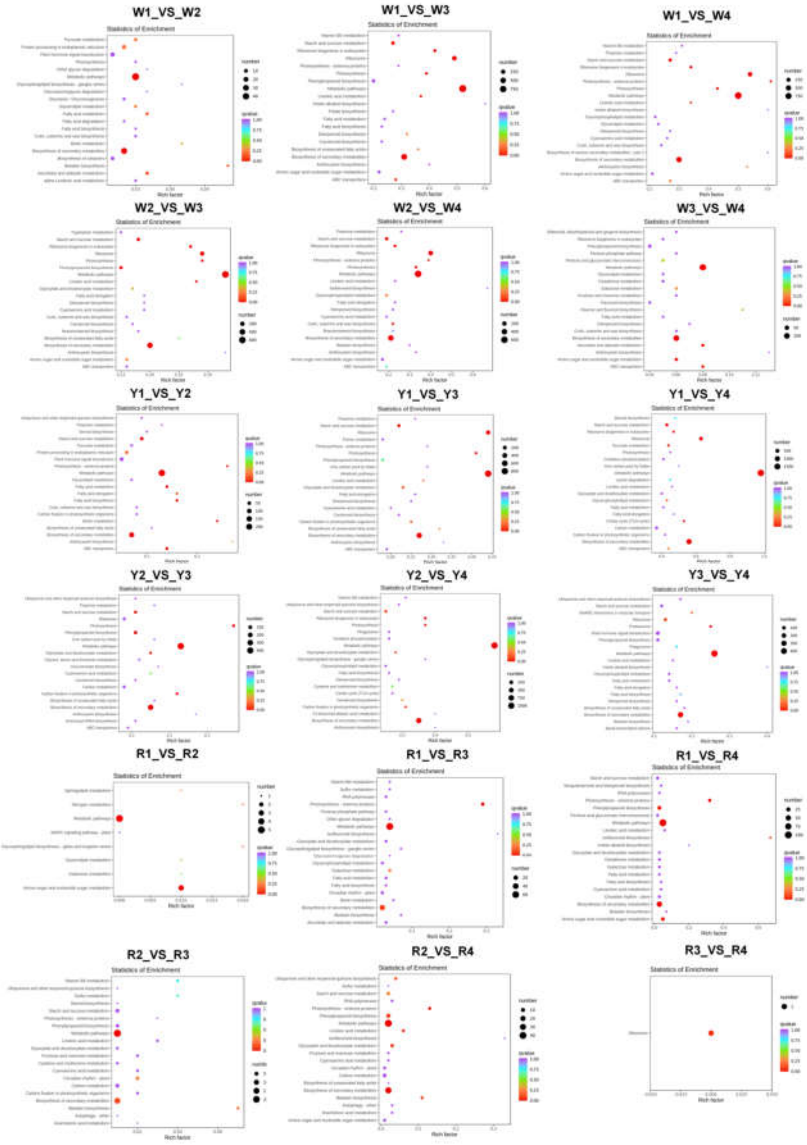
W1_VS_W2
W1_VS_W3
W1_VS_W4
W2_VS_W3
W2_VS_W4
W3_VS_W4
Y1_VS_Y2
Y1_VS_Y3
Y1_VS_Y4
Y2_VS_Y3
Y2_VS_Y4
Y3_VS_Y4
R1_VS_R2
R1_VS_R3
R1_VS_R4
R2_VS_R3
R2_VS_R4
R3_VS_R4

B-2

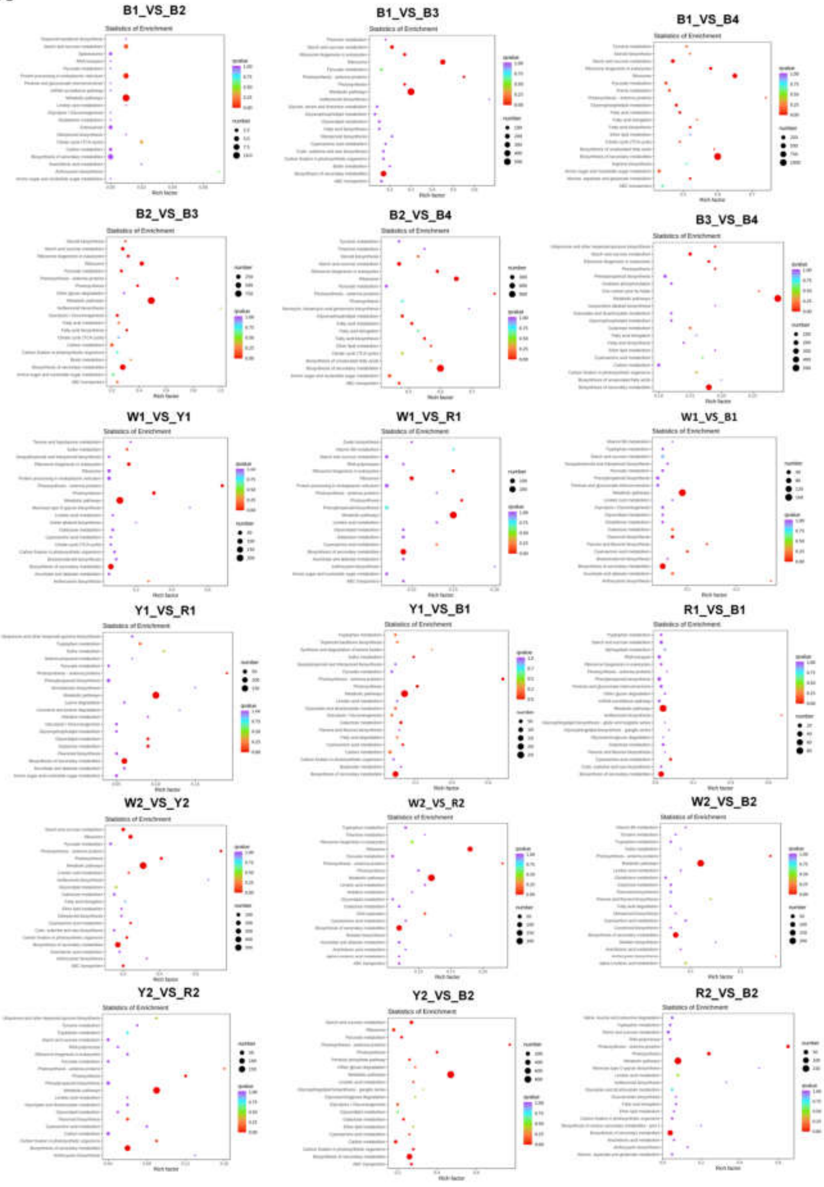
B1_VS_B2
B1_VS_B3
B1_VS_B4
B2_VS_B3
B2_VS_B4
B3_VS_B4
W1_VS_Y1
W1_VS_R1
W1_VS_B1
Y1_VS_R1
Y1_VS_B1
R1_VS_B1
W2_VS_Y2
W2_VS_R2
W2_VS_B2
Y2_VS_R2
Y2_VS_B2
R2_VS_B2

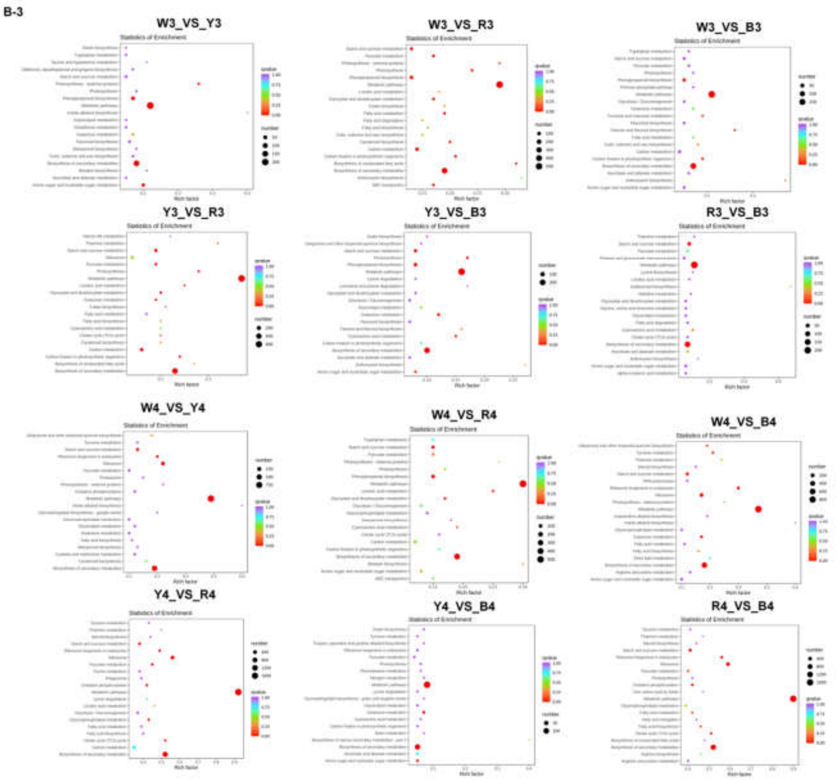

Fig. S4 (B) The enrichment scatter plot depicts KEGG pathways on the vertical axis and the Rich Factor on the horizontal axis. A higher Rich Factor indicates a greater degree of enrichment. The size of the dot corresponds to the number of differentially expressed genes enriched in the pathway; larger dots signify more genes. The color of the dot increasingly trending towards red indicates more significant enrichment.

C-1

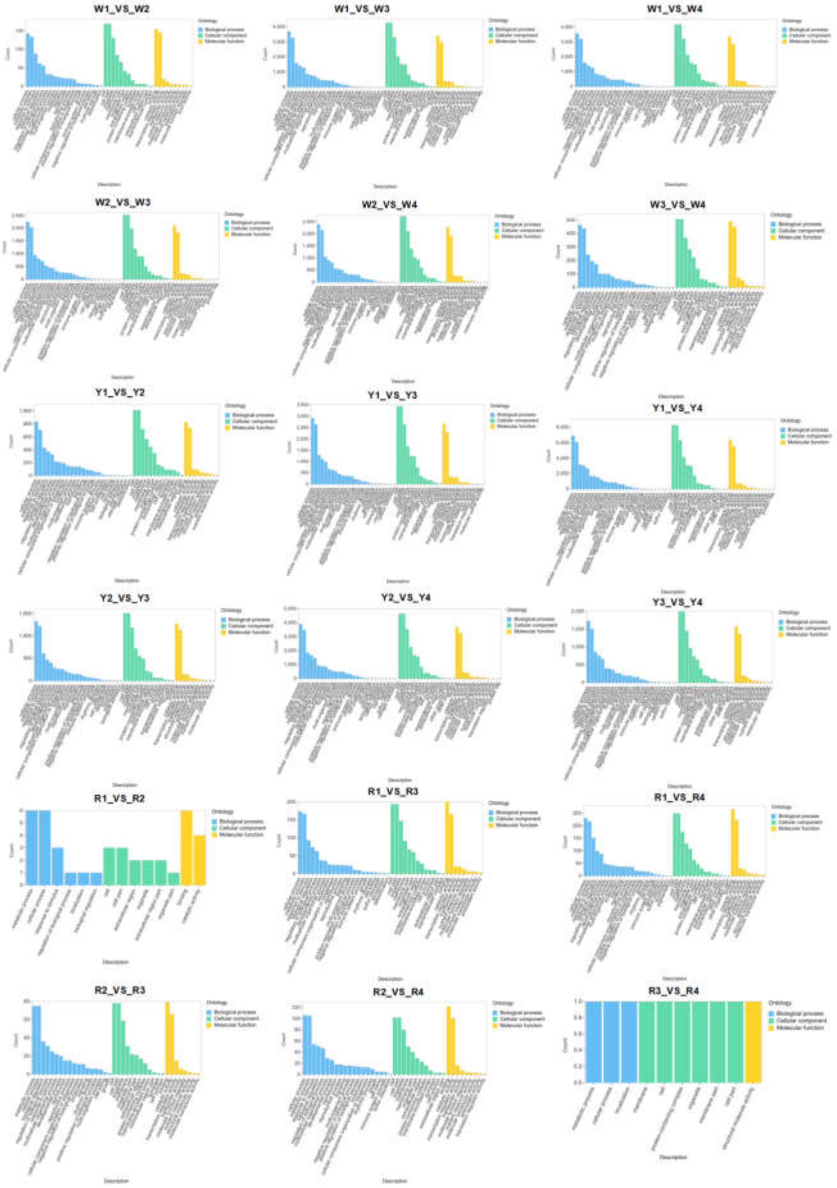
W1_VS_W2
W1_VS_W3
W1_VS_W4
W2_VS_W3
W2_VS_W4
W3_VS_W4
Y1_VS_Y2
Y1_VS_Y3
Y1_VS_Y4
Y2_VS_Y3
Y2_VS_Y4
Y3_VS_Y4
R1_VS_R2
R1_VS_R3
R1_VS_R4
R2_VS_R3
R2_VS_R4
R3_VS_R4
Ontology
Biological process
Cellular component
Molecular function
Count
Description

C-2

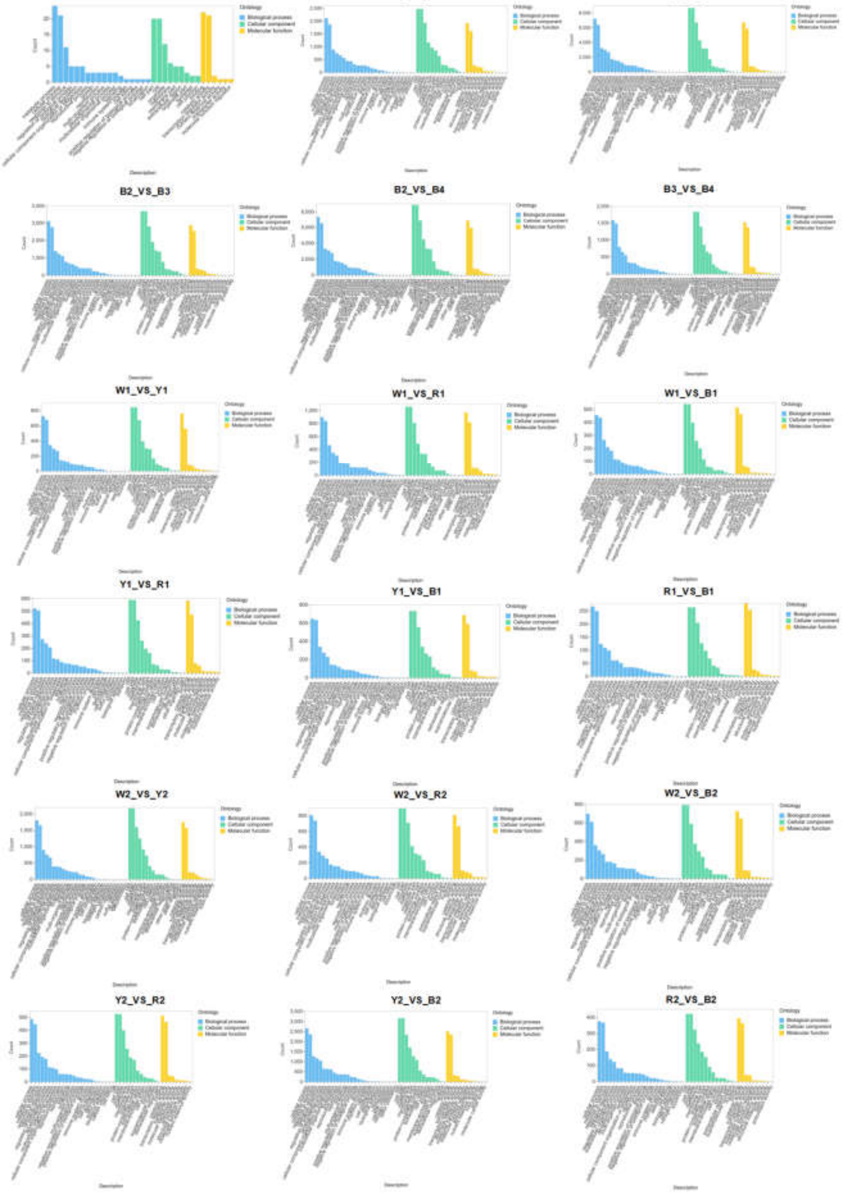
B1_VS_B2
B1_VS_B3
B1_VS_B4
B2_VS_B3
B2_VS_B4
B3_VS_B4
W1_VS_Y1
W1_VS_R1
W1_VS_B1
Y1_VS_R1
Y1_VS_B1
R1_VS_B1
W2_VS_Y2
W2_VS_R2
W2_VS_B2
Y2_VS_R2
Y2_VS_B2
R2_VS_B2

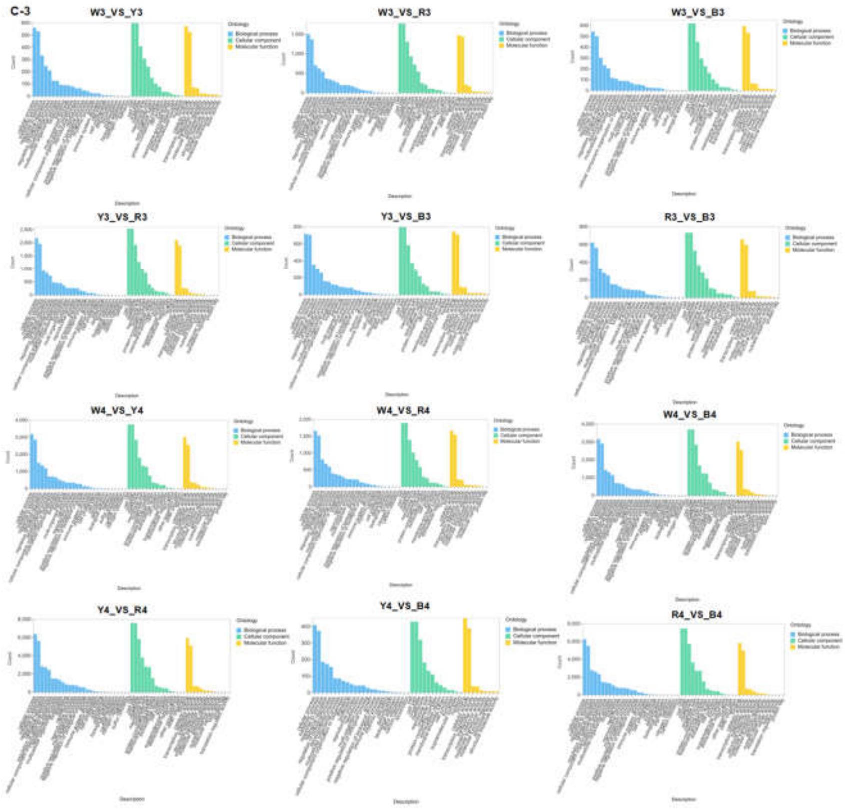

Fig. S4 (C) The differential gene category bar chart presents secondary GO (Gene Ontology) terms on the horizontal axis and the number of differential genes associated with each GO term on the vertical axis.

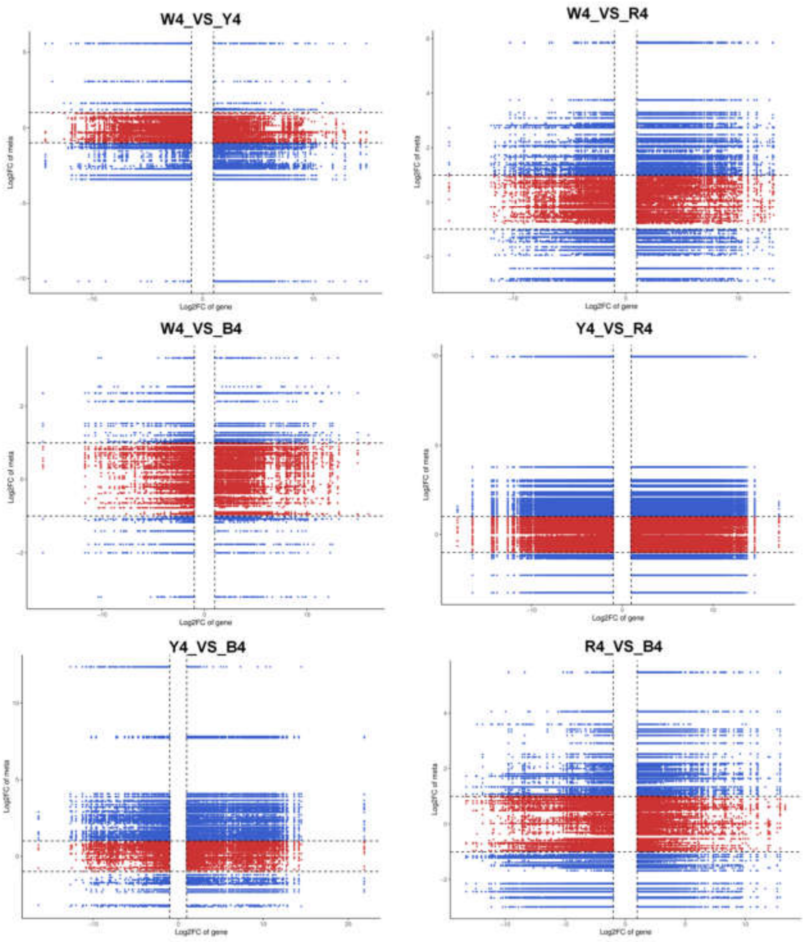

Fig. S5 Nine quadrant diagram of correlation analysis, the correlation analysis nine-quadrant plot showcases the log2 fold change (FC) of genes on the horizontal axis and the log2 FC of metabolites on the vertical axis.

Table S1. Primers used in the study.

	Genes	Forward primer (5' to 3')	Reverse primer (5' to 3')	Size
starch (Red VS Black)	gene-LOC110716805	TGGCTCACTGGAGAAACCAC	AAGTGTGCACCAACATTCGC	117
	gene-LOC110722789	CACTGATCGGCTTCATCGGT	TTCCCAGGACAACGACTTGG	108
	gene-LOC110738785	ACGGAACCAAAGCCGAATTG	TCATCAGGCGTTCCACCTTC	151
	gene-LOC110720405	AGCATGGTGGCTCCTTTCAA	GCATTGGACTCTTCCACCGT	108
	gene-LOC110730081	CTGACAAGTGTGAGCAGACCA	GCTGCTCCTCCAAACACACT	140
	gene-LOC110692055	GAACCTATCCTTGGAGGGCG	GCTGGGCCAAGACCTACTTC	128
Lipid (Yellow VS Black)	gene-LOC110732328	CAATTGGATGAAGGCCGGGA	AGGTGTTGTGCAACTCGACA	113
	gene-LOC110701563	ATGCCTCTGGATCTTGTGGA	TGGGATAGCTCGGTTGAGGT	145
	gene-LOC110699636	GCCAATTTCGCCTCTGCTTC	CGCGTTGGTTTTTCCTGCAA	138
	gene-LOC110709273	AGGCTACTGGAAGGTTCTGC	ATAACCGGAACCAGCAGACG	155
	gene-LOC110715590	GCATCTGCTGGATCAGGCTA	ACTGTCACCTGCCACCTTTA	123
	gene-LOC110728838	AACCGCAGGTCCCTTGTTAC	TCGATACCGTAAGGCGGGTA	109
	gene-LOC110710842	GGGCACAACATCGAGAAGAC	CGTCACCCCCTGCGTTTAT	150
Protein (White VS Black)	gene-LOC110720003	TGTACTCTAGTTGCCTTCCGC	TTGGGGGTTTAACAGCAGCA	157
	gene-LOC110687170	CGTCTCCAAAGGCGAGGTTT	AACTAGGCAACAAGACGCGA	177
	gene-LOC110716004	TACTGGAGGCCCTGGTTGTT	GTTGGTGCTTCTGAGTGCCA	102
	gene-LOC110702086	ATGCCTGGGATCATGCTGTT	AGATAGGCGCGTACAAGCTG	162
	gene-LOC110724454	GCCTTGGCAGACTTGTTCCT	TCTGGGGCATCTGGTTGTTT	161
	gene-LOC110724577	TTGTTGAGGCGCATAGTGGT	TCCTCTGACTCAACAGCCCT	133
	gene-LOC110704171	TAGGTGTGTTGGTACGCCAG	TGTCGTTGTTGCATTCGCTG	143
	gene-LOC110686607	ACATACCTAGCGCAGCATCC	TCTTGCCAACAACCTGAGCA	190
参数基因	CqActin1	GTACGCATGGGTGCTTGACAAACT	ATCAGCCTGGGAGGTACCAGTAAT	
	CqActin2	GTCCACAGAAAGTGCTTCTAAG	AACAACTCCTCACCTTCTCATG	

Table S2. Abundances of differentially expressed metabolites in experimental samples.

Group name	Up-regulated genes	Down-regulated genes	Significant DEGs
W_vs_Y	5	48	53
W_vs_R	15	10	25
W_vs_B	71	25	96
Y_vs_R	62	8	70
Y_vs_B	103	15	118
R_vs_B	65	27	92

Table S3 Quality statistics of quinoa transcriptome sequencing.

Sample	Raw Reads	Clean Reads	Clean Base (G)	Error Rate (%)	Q20 (%)	Q30 (%)	GC Content (%)
B1-1	54096608	51608282	7.74	0.02	98.06	94.32	44.17
B1-2	61401144	58834158	8.83	0.03	98.01	94.21	44.33
B1-3	44395350	42475790	6.37	0.03	97.99	94.15	44.5
B2-1	47087106	45076340	6.76	0.02	98.06	94.32	44.09
B2-2	48329496	46187092	6.93	0.03	97.94	94.02	44.78
B2-3	47028488	45045024	6.76	0.03	98.03	94.19	44.58
B3-1	47988552	47018382	7.05	0.03	97.98	94.21	44.87
B3-2	53241450	50651534	7.6	0.03	97.94	94.15	44.88
B3-3	62613596	60874680	9.13	0.03	97.77	93.48	44.14
B4-1	53500840	52016168	7.8	0.02	98.01	94.4	45.09
B4-2	45408804	44090358	6.61	0.02	98.06	94.37	44
B4-3	53813196	51330928	7.7	0.03	97.99	94.22	44.2
R1-1	51021298	48740362	7.31	0.02	98.08	94.42	44.46
R1-2	54196498	51826622	7.77	0.03	98.04	94.28	44.26
R1-3	46311684	44077628	6.61	0.03	97.39	92.8	44.47
R2-1	49232862	47631572	7.14	0.03	97.89	94.02	44.02
R2-2	51674356	49237000	7.39	0.02	98.1	94.46	44.5
R2-3	44925954	42088864	6.31	0.03	97.47	92.97	44.8
R3-1	66786986	64479608	9.67	0.03	97.98	94.23	45.25
R3-2	48115050	46283312	6.94	0.03	98.01	94.3	45.78
R3-3	53646258	51052602	7.66	0.02	98.08	94.4	44.96
R4-1	52806648	50687776	7.6	0.03	97.99	94.23	45.14
R4-2	51931882	49359080	7.4	0.03	97.86	93.83	44.81
R4-3	52818358	49110568	7.37	0.03	97.98	94.23	45.72
W1-1	51314148	48338758	7.25	0.03	97.97	94.13	43.83
W1-2	49640726	46661282	7	0.02	98.08	94.41	44.12
W1-3	46766966	44014206	6.6	0.02	98.07	94.4	44.05
W2-1	47070410	44411676	6.66	0.03	98	94.24	43.85
W2-2	48039052	45238176	6.79	0.02	98.08	94.39	44.41
W2-3	51682818	48153110	7.22	0.03	98.02	94.28	44.94
W3-1	44205582	41961984	6.29	0.03	98.02	94.24	43.71
W3-2	44406054	42004898	6.3	0.03	97.98	94.23	44.9
W3-3	48546372	46176266	6.93	0.02	98.09	94.44	43.86
W4-1	44527432	41906790	6.29	0.03	98.02	94.28	44.01
W4-2	51191394	48749892	7.31	0.03	97.95	94.16	44.22
W4-3	47559620	44552948	6.68	0.03	97.92	94.08	44.47
Y1-1	51006906	47949360	7.19	0.03	97.94	94.04	44.22
Y1-2	43074092	39993932	6	0.03	97.43	92.83	43.91
Y1-3	50887776	48117848	7.22	0.03	98.04	94.3	44.44
Y2-1	47228280	45024308	6.75	0.02	98.05	94.41	44.86
Y2-2	45845776	43530728	6.53	0.03	97.83	93.84	44.37

Y2-3	51942858	48706152	7.31	0.03	98.02	94.26	44.54
Y3-1	51054986	49044174	7.36	0.02	98.09	94.48	43.95
Y3-2	50372706	47775818	7.17	0.03	97.91	94.01	44.15
Y3-3	49717164	47454288	7.12	0.03	98.01	94.24	43.84
Y4-1	47402274	45653896	6.85	0.02	98.02	94.35	45.67
Y4-2	45762090	43499124	6.52	0.03	98.04	94.31	43.93
Y4-3	54076248	51774578	7.77	0.03	97.92	94.22	45.06

Table S4 Abundances of differentially expressed genes in experimental samples.

Group name	Up-regulated genes	Down-regulated genes	Total DEGs
W1_vs_W2	253	345	625
W1_vs_W3	5057	3418	8789
W1_vs_W4	4995	3546	8807
W2_vs_W3	3327	1776	5288
W2_vs_W4	3770	2457	6472
W3_vs_W4	678	1039	1735
Y1_vs_Y2	705	1349	2140
Y1_vs_Y3	3842	2642	6863
Y1_vs_Y4	7194	8801	16020
Y2_vs_Y3	1906	1104	3221
Y2_vs_Y4	4623	4345	9387
Y3_vs_Y4	2308	1831	4312
R1_vs_R2	7	5	16
R1_vs_R3	363	296	713
R1_vs_R4	452	451	939
R2_vs_R3	124	186	310
R2_vs_R4	193	263	456
R3_vs_R4	3	2	5
B1_vs_B2	23	30	63
B1_vs_B3	2444	2342	4988
B1_vs_B4	7385	9237	16633
B2_vs_B3	3379	4015	7411
B2_vs_B4	7609	9538	17157
B3_vs_B4	1930	1519	4014
W1_vs_Y1	1401	937	2338
W1_vs_R1	1726	1279	3005
W1_vs_B1	1224	568	1871
Y1_vs_R1	1176	845	2022
Y1_vs_B1	1018	1269	2321
R1_vs_B1	402	609	1012
W2_vs_Y2	2104	2920	5155
W2_vs_R2	1707	970	2683
W2_vs_B2	1501	916	2506
Y2_vs_R2	1168	561	1803
Y2_vs_B2	4257	2663	7151
R2_vs_B2	591	559	1277
W3_vs_Y3	1018	789	2015
W3_vs_R3	1734	2524	4472
W3_vs_B3	1000	1156	2217
Y3_vs_R3	2519	3462	6252
Y3_vs_B3	880	1505	2655
R3_vs_B3	960	1105	2212

W4_vs_Y4	4276	3499	8253
W4_vs_R4	2093	2902	5180
W4_vs_B4	4171	3535	8116
Y4_vs_R4	8099	7418	15655
Y4_vs_B4	990	655	1664
R4_vs_B4	6867	7853	14782

Table S6.Differential expression of lipid anabolism and regulatory genes in four quinoa cultivars.

	Name	ID	log2FoldChange	regulated	KEGG
Y_VS_B	phospholipase	gene-LOC110690870	2.018512315	up	K01047 secretory phospholipase A2
Y_VS_B		gene-LOC110719406	4.115241091	up	K16860 phospholipase
Y_VS_B		gene-LOC110721654	2.229687324	up	K01115 phospholipase
Y_VS_B		gene-LOC110703973	2.315805139	up	K01114 phospholipase
Y_VS_B		gene-LOC110707948	2.025634362	up	K01115 phospholipase
Y_VS_B		gene-LOC110720422	2.12490006	up	K01114 phospholipase
Y_VS_B		gene-LOC110730326	3.357450167	up	K16818 phospholipase
Y_VS_B	acyltransferase	gene-LOC110701190	2.959334245	up	K13508 glycerol-3-phosphate acyltransferase
Y_VS_B		gene-LOC110705050	2.393136101	up	K13513 lysocardiolipin and lysophospholipid acyltransferase
Y_VS_B		gene-LOC110705915	11.278536521	up	K07513 acetyl-CoA acyltransferase 1
Y_VS_B		gene-LOC110725144	2.496012216	up	--
Y_VS_B		gene-LOC110728089	2.174885798	up	K07513 acetyl-CoA acyltransferase 1
Y_VS_B		gene-LOC110733003	2.739921015	up	K20027 protein S-acyltransferase 18-like
Y_VS_B		gene-LOC110734253	2.902489631	up	K20027 protein S-acyltransferase 18-like
Y_VS_B		gene-LOC110682398	3.83973578	up	--
Y_VS_B		gene-LOC110701189	2.478499908	up	K13508 glycerol-3-phosphate acyltransferase
Y_VS_B	GDSL	gene-LOC110695766	2.008666078	up	--
Y_VS_B		gene-LOC110696324	3.891072706	up	--
Y_VS_B		gene-LOC110697429	2.596250312	up	--
Y_VS_B		gene-LOC110699636	3.676911891	up	--
Y_VS_B		gene-LOC110701532	2.221307865	up	--
Y_VS_B		gene-LOC110708586	2.036857308	up	--
Y_VS_B		gene-LOC110709273	3.586487937	up	--
Y_VS_B		gene-LOC110715590	4.449791538	up	--
Y_VS_B		gene-LOC110728838	3.320450486	up	--
Y_VS_B		gene-LOC110729899	2.338297313	up	--
Y_VS_B		gene-LOC110735188	2.615743847	up	--
Y_VS_B		gene-LOC110736706	3.689950015	up	--
Y_VS_B		gene-LOC110691892	2.969890679	up	--
Y_VS_B		gene-LOC110724356	2.70065839	up	--
Y_VS_B		gene-LOC110701563	3.728743031	up	--
Y_VS_R	long chain acyl-CoA	gene-LOC110732999	2.073182437	up	K01897 long-chain acyl-CoA synthetase

Y_VS_R	synthetase 1-like	gene-LOC110734248	4.779350197	up	K01897 long-chain acyl-CoA synthetase
Y_VS_R	acyl-[acyl-carrier-protein] desaturase	gene-LOC110738720	3.070093996	up	K03921 acyl-[acyl-carrier-protein] desaturase
Y_VS_B		gene-LOC110701189	2.478499908	up	K13508 glycerol-3-phosphate acyltransferase
Y_VS_B	non-specific phospholipase C4-like	gene-LOC110720422	2.12490006	up	K01114 non-specific phospholipase C4-like

Table S7.Amino acid biosynthesis and differential expression of regulatory genes in four quinoa cultivars.

	Name	ID	log2FoldChange	regulated	KEGG
W_VS_B	cysteine protease	gene-LOC110682345	2.822007231	up	K16290 xylem cysteine proteinase
W_VS_B		gene-LOC110697218	2.101185917	up	K16292 KDEL-tailed cysteine endopeptidase
W_VS_B		gene-LOC110707153	2.070623903	up	K16292 KDEL-tailed cysteine endopeptidase
W_VS_B		gene-LOC110717421	2.583938352	up	K18342 OTU domain-containing protein 6
Y_VS_R	S-adenosylmethionine decarboxylase proenzyme-like	gene-LOC110686006	2.01411126	up	K01611 S-adenosylmethionine decarboxylase
W_VS_B	S-adenosylmethionine synthetase	gene-LOC110732957	-1.866631126	down	K00789 S-adenosylmethionine synthetase
W_VS_B		gene-LOC110734214	-1.449182529	down	K00789 S-adenosylmethionine synthetase
W_VS_B	serine carboxypeptidase	gene-LOC110702086	2.07508958	up	K16297 serine carboxypeptidase-like clade II
W_VS_B		gene-LOC110716004	4.836182431	up	K16296 serine carboxypeptidase-like clade I
W_VS_B		gene-LOC110702087	-2.973743546	down	K16297 serine carboxypeptidase-like clade II
W_VS_B		gene-LOC110725795	-3.242111323	down	K16297 serine carboxypeptidase-like clade II
W_VS_B		gene-LOC110735784	-2.97490357	down	K16297 serine carboxypeptidase-like clade II
W_VS_B		gene-LOC110683521	-4.049796083	down	K16297 serine carboxypeptidase-like clade II
W_VS_B		gene-LOC110694414	-1.6194035	down	K09645 vitellogenic carboxypeptidase-like protein
W_VS_B		gene-LOC110700281	-1.307843198	down	K16296 serine carboxypeptidase-like clade I
W_VS_B		gene-LOC110716913	-2.926401022	down	K16296 serine carboxypeptidase-like clade I
W_VS_B		gene-LOC110717872	-1.222901754	down	K16296 serine carboxypeptidase-like clade I
W_VS_B		gene-LOC110722155	5.79306333	up	K16296 serine carboxypeptidase-like clade I
W_VS_B		gene-LOC110738566	-3.435078188	down	K16296 serine carboxypeptidase-like clade I
W_VS_B	Glutamate acetyltransferase	gene-LOC110708548	-1.897733611	down	K00620 glutamate N-acetyltransferase

W_VS_B	Glutamate acetyltransferase	gene-LOC110722428	-1.836363761	down	K01915 glutamine synthetase
W_VS_B	arginine decarboxylase-like	gene-LOC110718863	-2.066920966	down	K01583 arginine decarboxylase
W_VS_B	tryptophan synthase beta chain 1-like	gene-LOC110723779	1.232752463	up	K01696 tryptophan synthase beta chain
W_VS_B		gene-LOC110687330	-1.310354151	down	K01696 tryptophan synthase beta chain
W_VS_B	alanine--glyoxylate aminotransferase	gene-LOC110698132	-1.815966938	down	K00827 alanine-glyoxylate transaminase

Table S8.Differential expression of embryo size regulatory genes in four quinoa varieties.

	Name	ID	log2FoldChange	regulated	KEGG
W_VS_B		gene-LOC110716604	2.889262665	up	uncharacterized protein LOC110716604
W_VS_B		gene-LOC110705031	5.088714453	up	late embryogenesis abundant protein 76-like
W_VS_B		gene-LOC110714155	2.946375253	up	desiccation protectant protein Lea14 homolog
W_VS_B		gene-LOC110683581	-3.28841043	down	NDR1/HIN1-like protein 1
W_VS_B		gene-LOC110688154	-2.594834767	down	NDR1/HIN1-like protein 1
W_VS_B		gene-LOC110690801	-2.62040035	down	uncharacterized protein LOC110690801
W_VS_B		gene-LOC110691091	-3.608463751	down	uncharacterized protein LOC110691091
W_VS_B		gene-LOC110697633	-2.877319788	down	NDR1/HIN1-like protein 6
W_VS_B	late embryogenesis abundant protein	gene-LOC110711156	-2.362261181	down	NDR1/HIN1-like protein 6
W_VS_B		gene-LOC110711957	-4.283661574	down	NDR1/HIN1-like protein 13
W_VS_B		gene-LOC110726609	-4.810095326	down	protein SENESCENCE-ASSOCIATED GENE 21, mitochondrial-like
W_VS_B		gene-LOC110727033	-4.371567585	down	late embryogenesis abundant protein At5g17165-like
W_VS_B		gene-LOC110730324	-3.605111351	down	NDR1/HIN1-like protein 3
W_VS_B		gene-LOC110730339	-3.986779036	down	late embryogenesis abundant protein At5g17165-like
W_VS_B		gene-LOC110731029	-3.458993141	down	NDR1/HIN1-like protein 3
W_VS_B		gene-LOC110731592	-2.261837273	down	late embryogenesis abundant protein Lea14-A-like
W_VS_B		gene-LOC110731974	-3.222967142	down	uncharacterized protein LOC110731974
W_VS_B		gene-LOC110733190	-3.737153456	down	NDR1/HIN1-like protein 6
W_VS_B		gene-LOC110740332	-4.080992078	down	NDR1/HIN1-like protein 13
W_VS_B		gene-LOC110731115	2.862959548	up	non-specific lipid-transfer protein-like
W_VS_B		gene-LOC110690672	-4.400402213	down	non-specific lipid-transfer protein-like
W_VS_B		gene-LOC110690674	-1.945161563	down	non-specific lipid-transfer protein-like
W_VS_B		gene-LOC110690676	-4.647566761	down	non-specific lipid-transfer protein-like
W_VS_B	non-specific lipid-transfer protein-like	gene-LOC110738300	-2.056741724	down	non-specific lipid-transfer protein-like protein At2g13820
W_VS_B		gene-LOC110687238	1.515748057	up	non-specific lipid-transfer protein-like
W_VS_B		gene-LOC110690671	-5.340938928	down	non-specific lipid-transfer protein-like
W_VS_B		gene-LOC110690675	-6.275814923	down	non-specific lipid-transfer protein-like
W_VS_B		gene-LOC110693486	2.398216793	up	non-specific lipid-transfer protein-like
W_VS_B		gene-LOC110697005	3.274617629	up	non-specific lipid-transfer protein-like protein At2g13820

W_VS_B	gene-LOC110716624	-1.860413629	down	non-specific lipid-transfer protein-like protein At5g64080
W_VS_B	gene-LOC110726962	2.458687902	up	non-specific lipid-transfer protein-like
W_VS_B	gene-LOC110729006	-3.22459051	down	non-specific lipid-transfer protein-like protein At2g13820
W_VS_B	gene-LOC110731103	-5.364425016	down	non-specific lipid-transfer protein-like

Table S9. The number of nutrient-related genes.

metabolic pathway	Total genes
glycometabolism	288
lipid metabolism	282
Starch and sucrose metabolism	158
Amino sugar and nucleotide sugar metabolism	71
Transcription factor	227
Glycine, serine and threonine metabolism	153
Alanine, aspartate and glutamate metabolism	27
Arginine and proline metabolism	48
Tryptophan metabolism	32
Lysine and histidine metabolism	71
Leucine metabolism	44
Tyrosine metabolism	14
Valine and isoleucine metabolism	7
Other biosynthesis of amino acids	60
late embryogenesis abundant protein	21
non-specific lipid-transfer protein	40
