# Supplementary material for: Metabolome and Transcriptome Association Analysis Reveals Mechanism of Synthesis of Nutrient Composition in Quinoa (Chenopodium quinoa Willd.) Seeds
Source: Foods. 2024 Apr 26;13(9):1325. doi: 10.3390/foods13091325 (PMC11082971; doi:10.3390/foods13091325)
Supplement: Supplementary file 1 [file foods-13-01325-s001.zip › supporting information.pptx]

## Slide 1
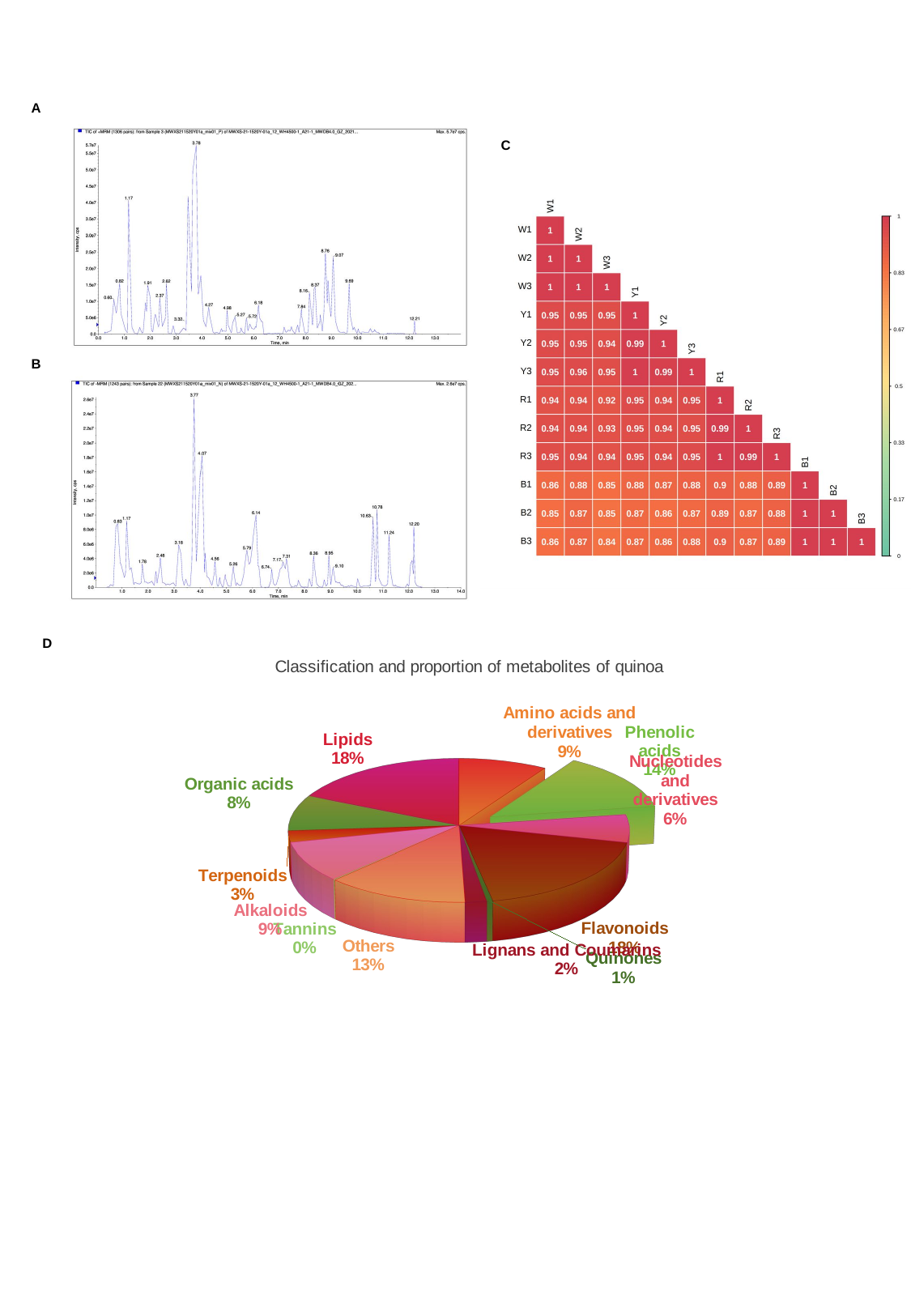

A
C
B
D
[unsupported chart]

## Slide 2
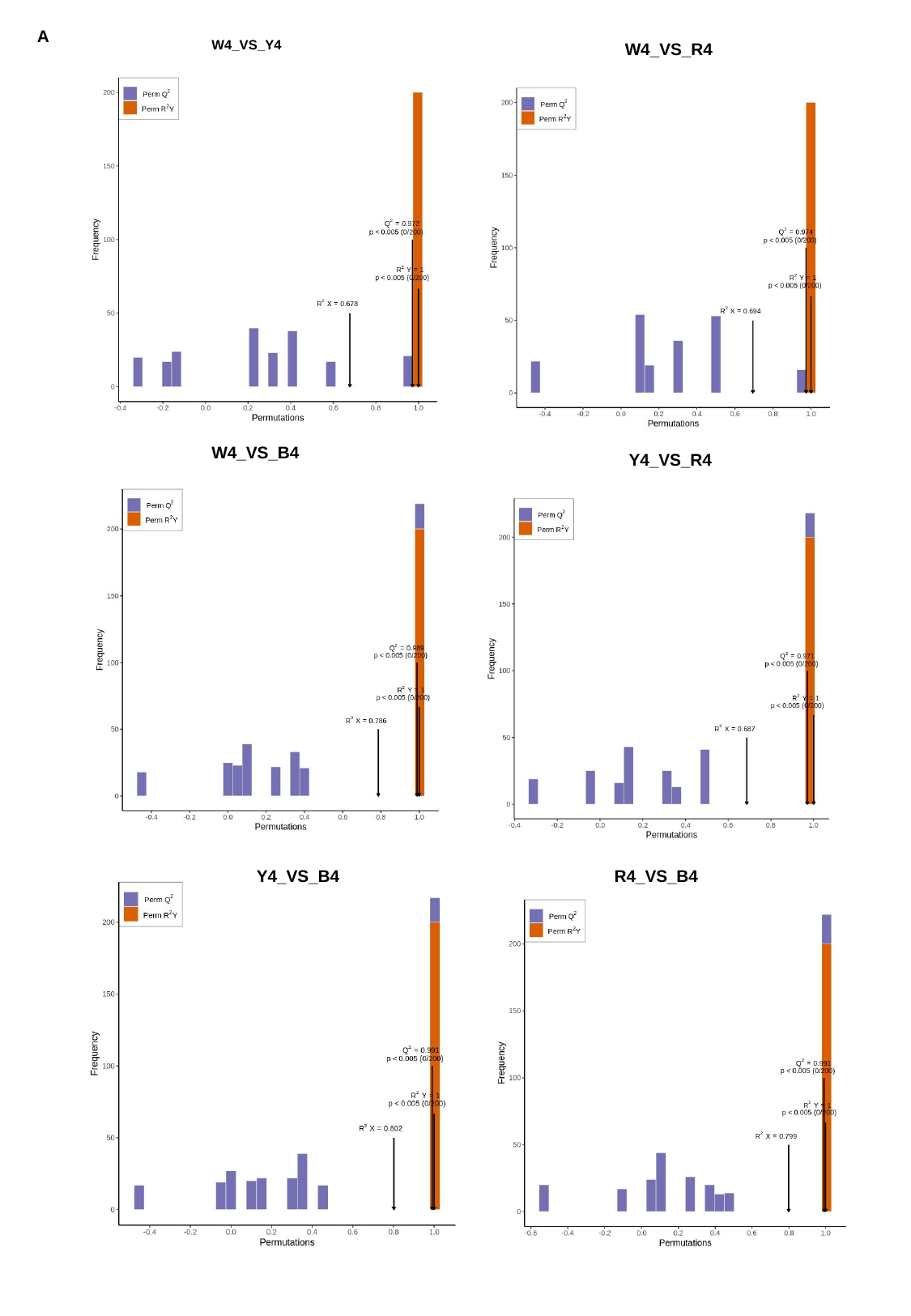

A
W4_VS_Y4
W4_VS_R4
W4_VS_B4
Y4_VS_R4
Y4_VS_B4
R4_VS_B4

## Slide 3
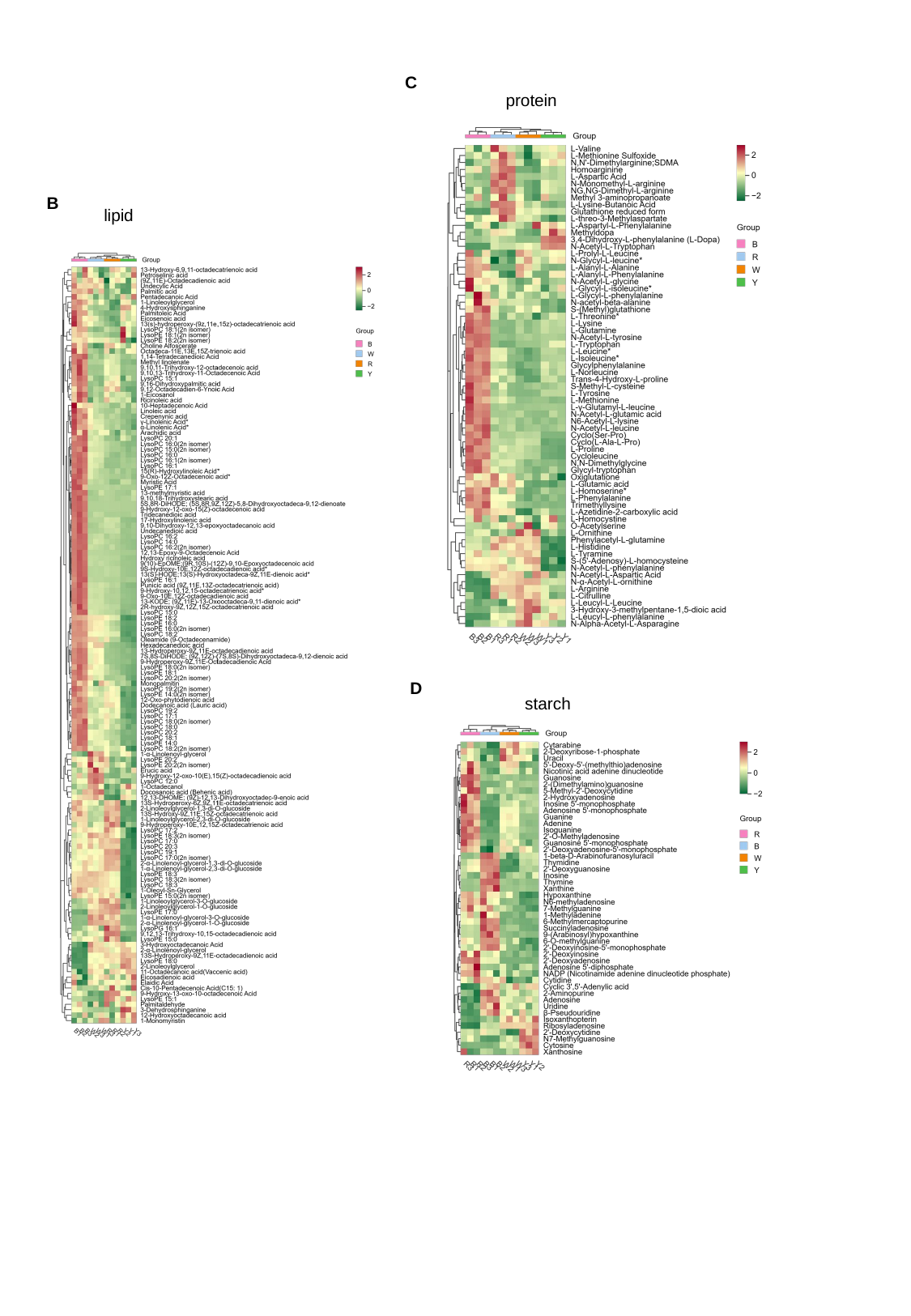

C
B
D
protein
lipid
starch

## Slide 4
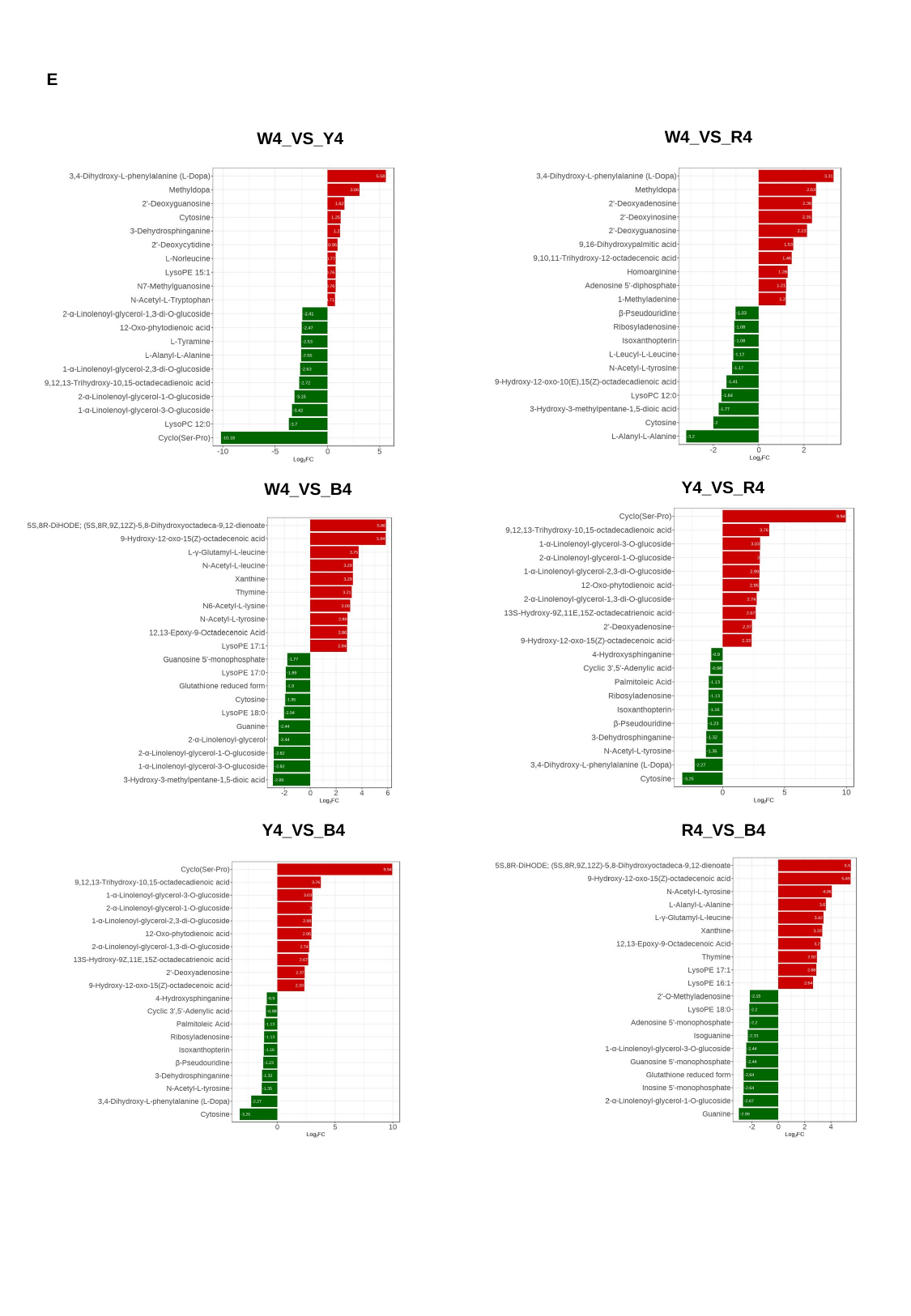

E
W4_VS_R4
W4_VS_Y4
Y4_VS_R4
W4_VS_B4
Y4_VS_B4
R4_VS_B4

## Slide 5
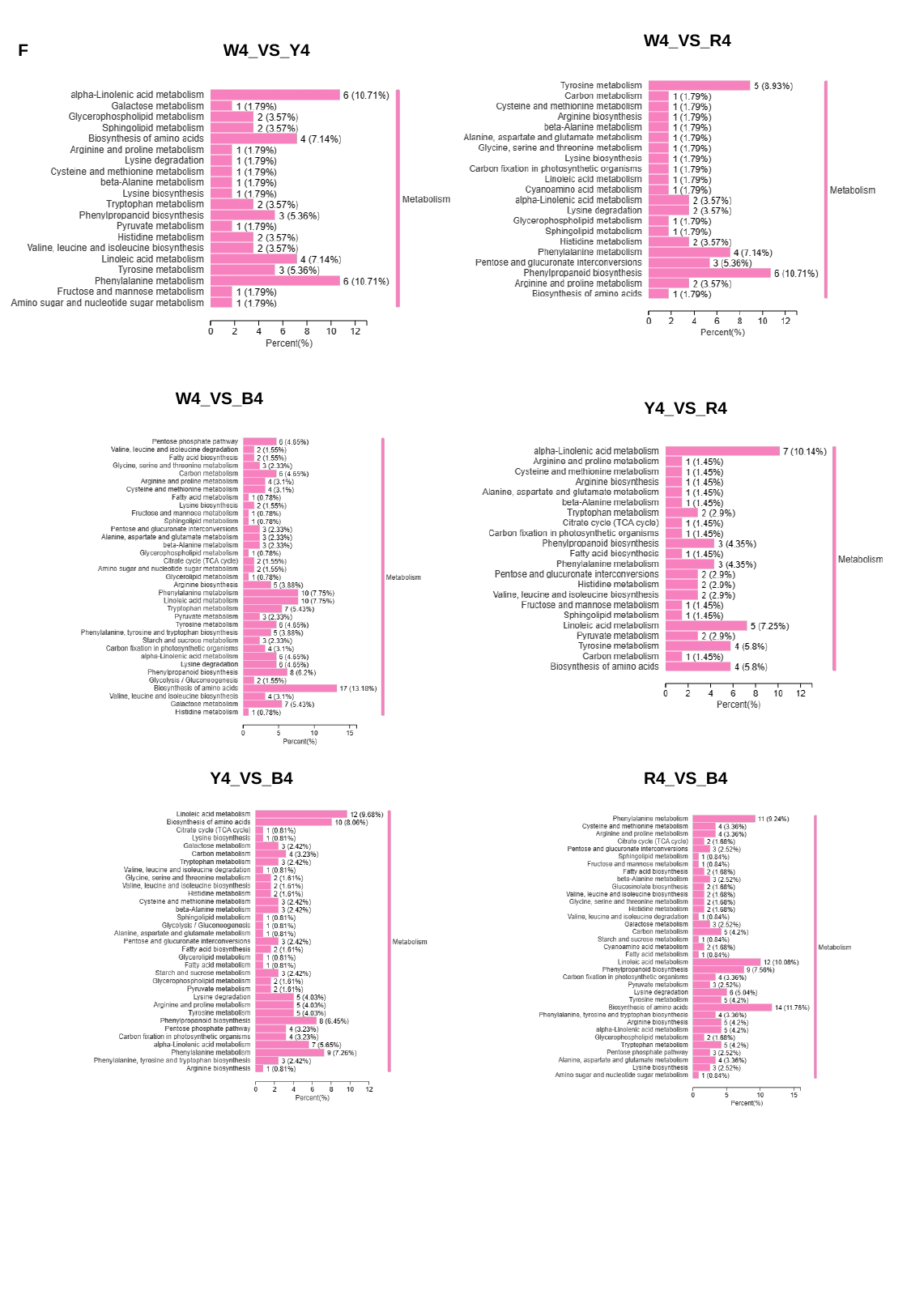

W4_VS_R4
F
W4_VS_Y4
W4_VS_B4
Y4_VS_R4
Y4_VS_B4
R4_VS_B4

## Slide 6
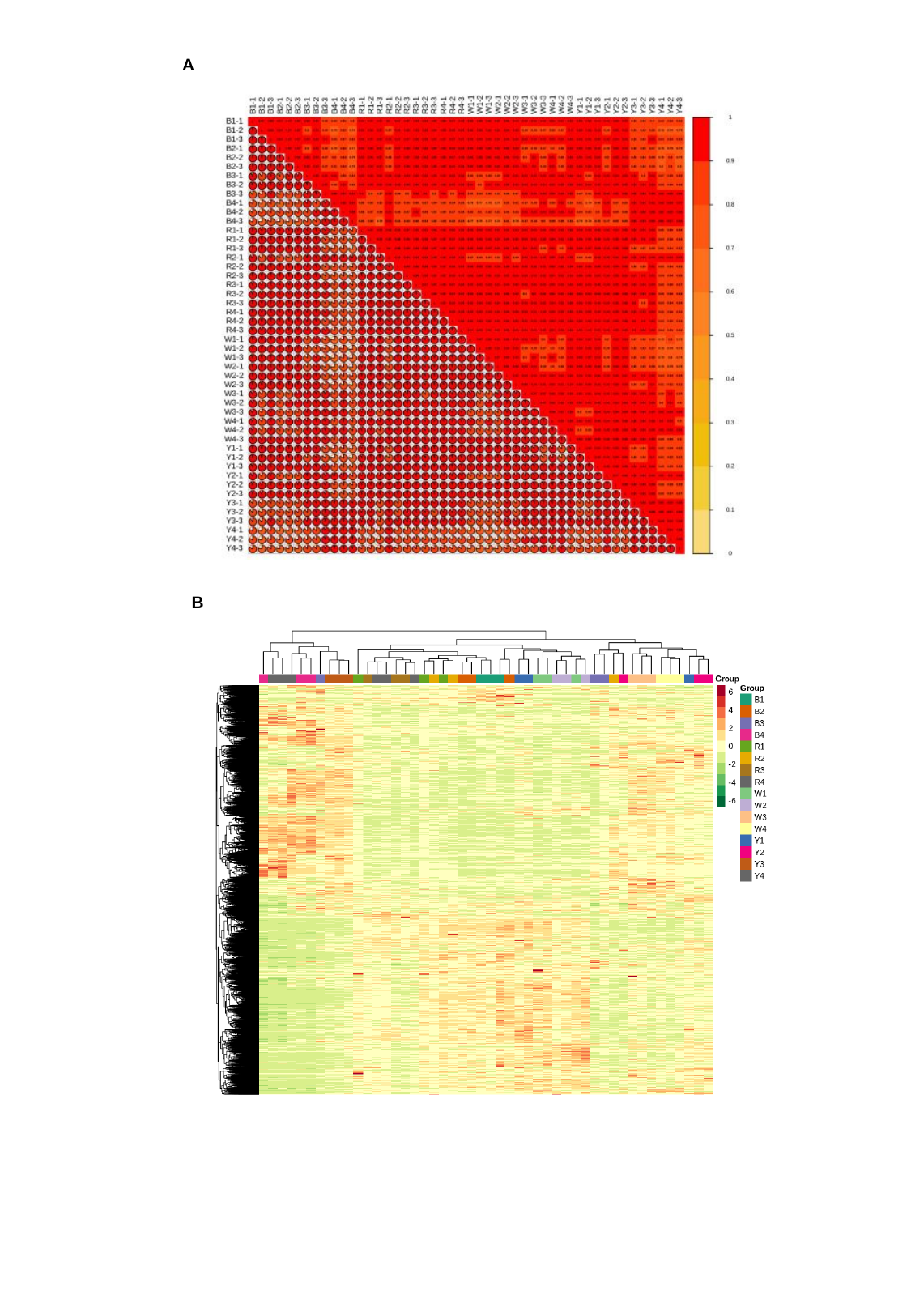

A
B

## Slide 7
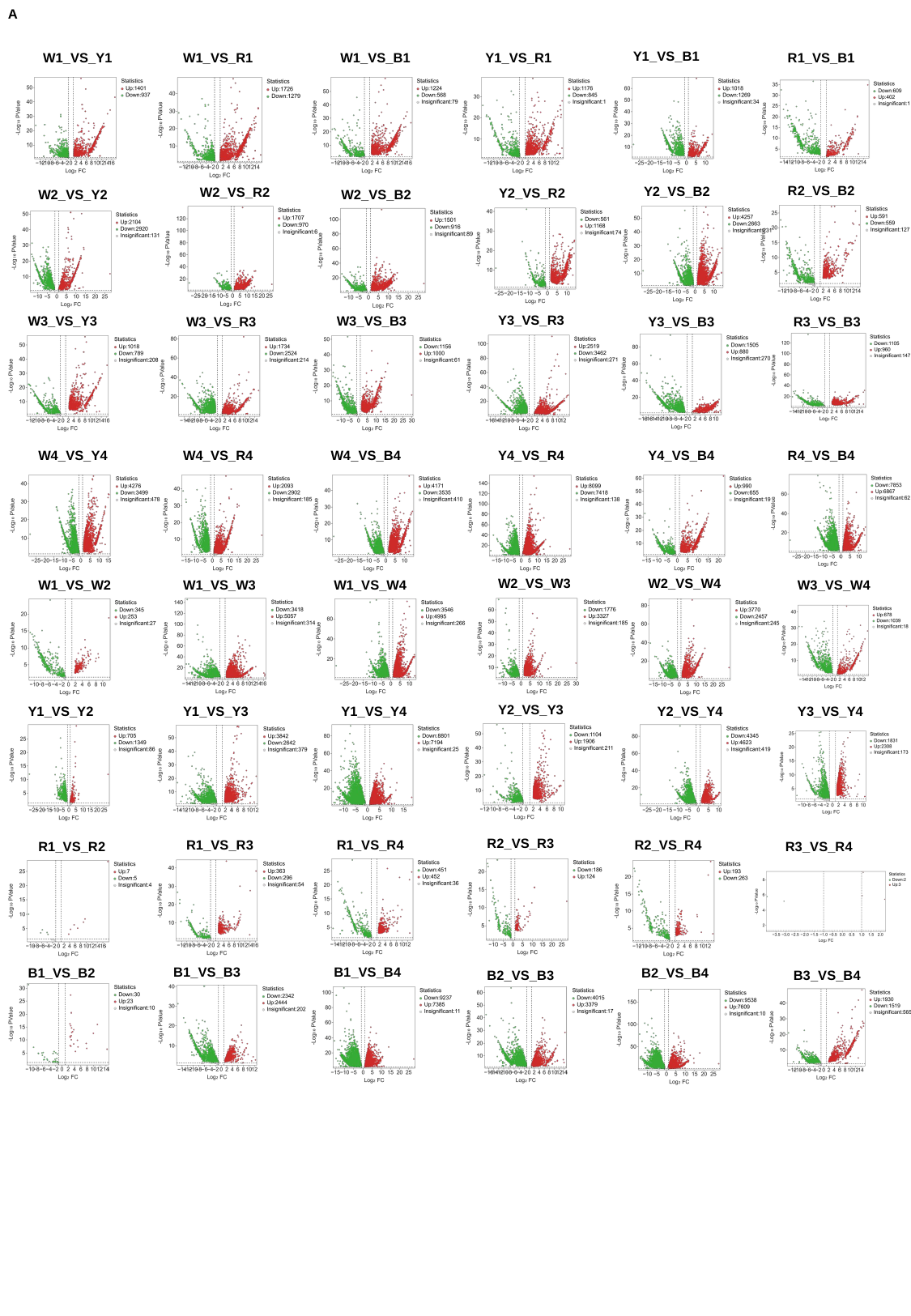

A
Y1_VS_B1
W1_VS_Y1
W1_VS_R1
W1_VS_B1
Y1_VS_R1
R1_VS_B1
R2_VS_B2
Y2_VS_B2
W2_VS_R2
Y2_VS_R2
W2_VS_Y2
W2_VS_B2
Y3_VS_R3
W3_VS_Y3
W3_VS_B3
W3_VS_R3
Y3_VS_B3
R3_VS_B3
W4_VS_Y4
R4_VS_B4
W4_VS_R4
W4_VS_B4
Y4_VS_R4
Y4_VS_B4
W2_VS_W3
W2_VS_W4
W1_VS_W2
W1_VS_W3
W1_VS_W4
W3_VS_W4
Y2_VS_Y3
Y1_VS_Y2
Y1_VS_Y3
Y1_VS_Y4
Y2_VS_Y4
Y3_VS_Y4
R2_VS_R3
R1_VS_R3
R1_VS_R4
R2_VS_R4
R1_VS_R2
R3_VS_R4
B1_VS_B3
B1_VS_B4
B1_VS_B2
B2_VS_B4
B2_VS_B3
B3_VS_B4

## Slide 8
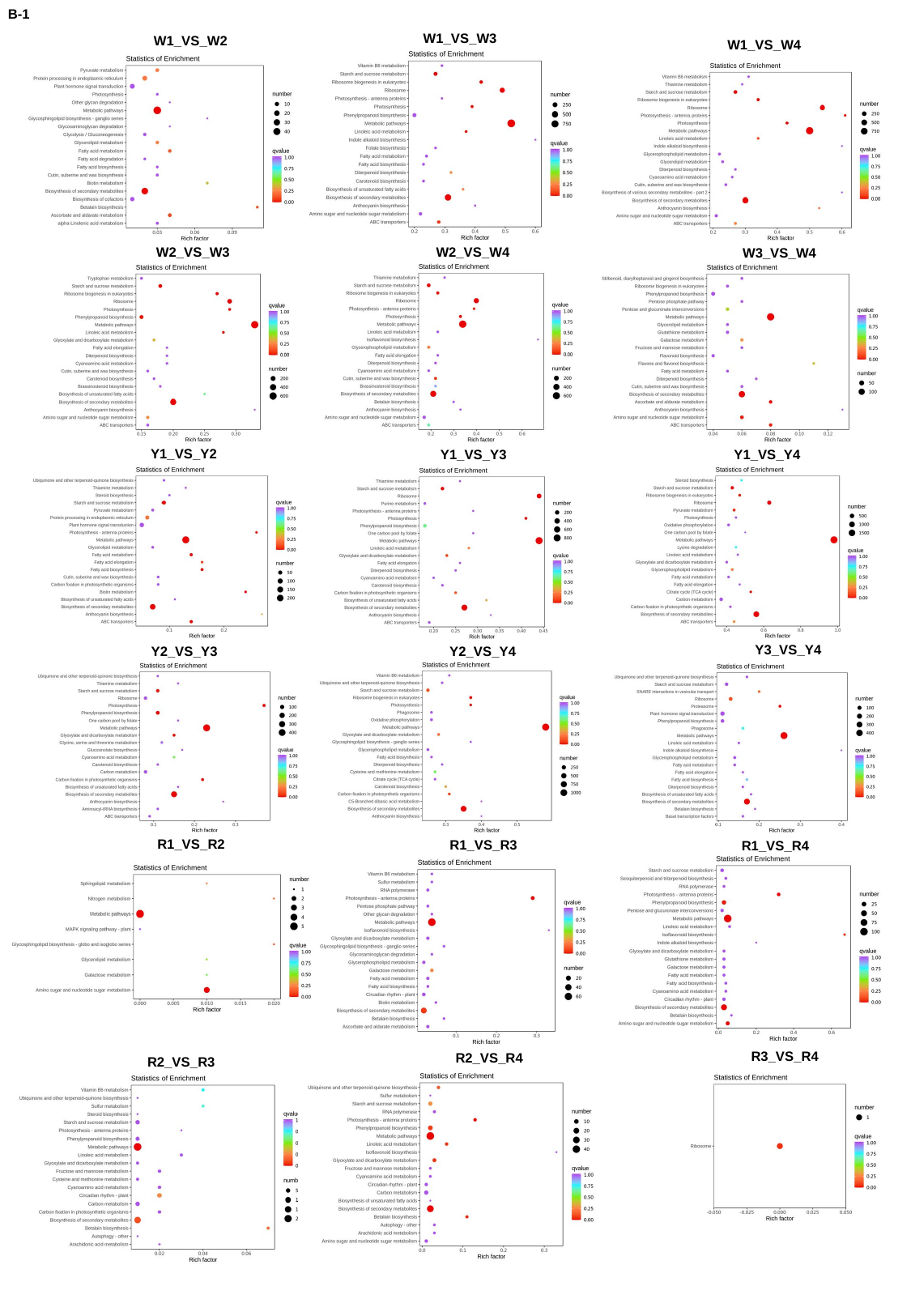

B-1
W1_VS_W3
W1_VS_W2
W1_VS_W4
W2_VS_W3
W2_VS_W4
W3_VS_W4
Y1_VS_Y2
Y1_VS_Y4
Y1_VS_Y3
Y3_VS_Y4
Y2_VS_Y3
Y2_VS_Y4
R1_VS_R2
R1_VS_R3
R1_VS_R4
R3_VS_R4
R2_VS_R4
R2_VS_R3

## Slide 9
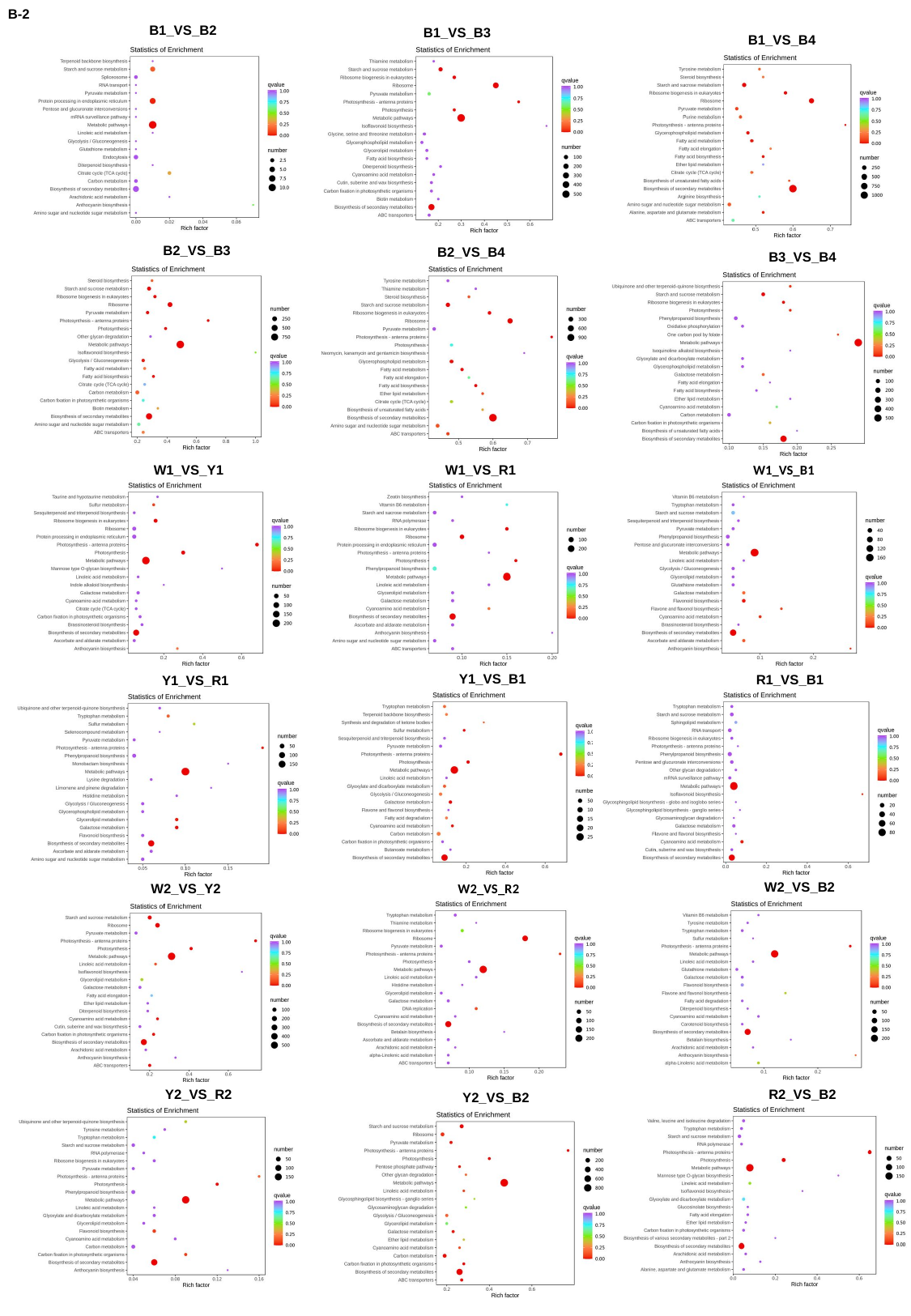

B-2
B1_VS_B2
B1_VS_B3
B1_VS_B4
B2_VS_B3
B2_VS_B4
B3_VS_B4
W1_VS_R1
W1_VS_B1
W1_VS_Y1
Y1_VS_B1
R1_VS_B1
Y1_VS_R1
W2_VS_B2
W2_VS_R2
W2_VS_Y2
Y2_VS_R2
R2_VS_B2
Y2_VS_B2

## Slide 10
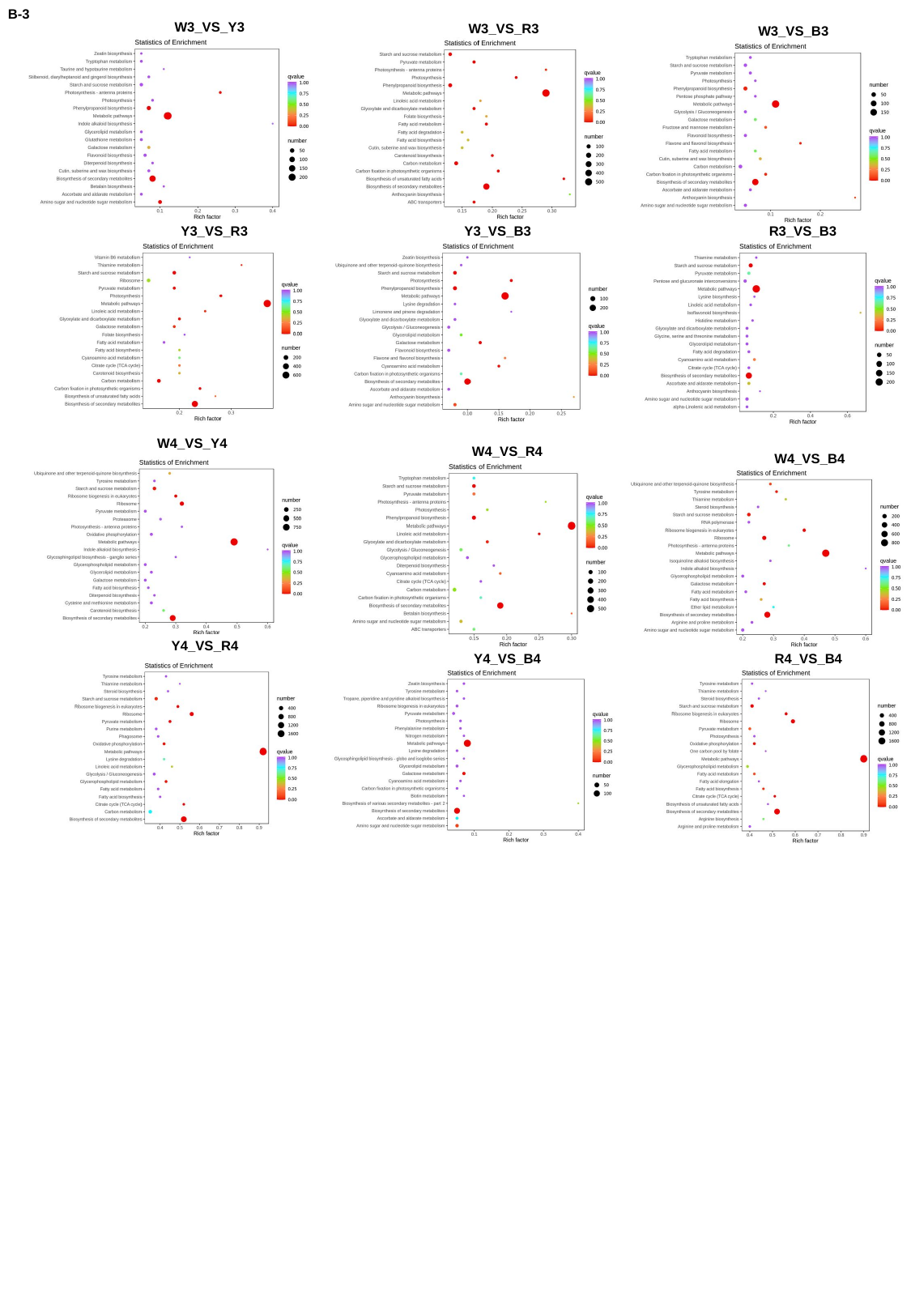

B-3
W3_VS_Y3
W3_VS_R3
W3_VS_B3
Y3_VS_R3
Y3_VS_B3
R3_VS_B3
W4_VS_Y4
W4_VS_R4
W4_VS_B4
Y4_VS_R4
R4_VS_B4
Y4_VS_B4

## Slide 11
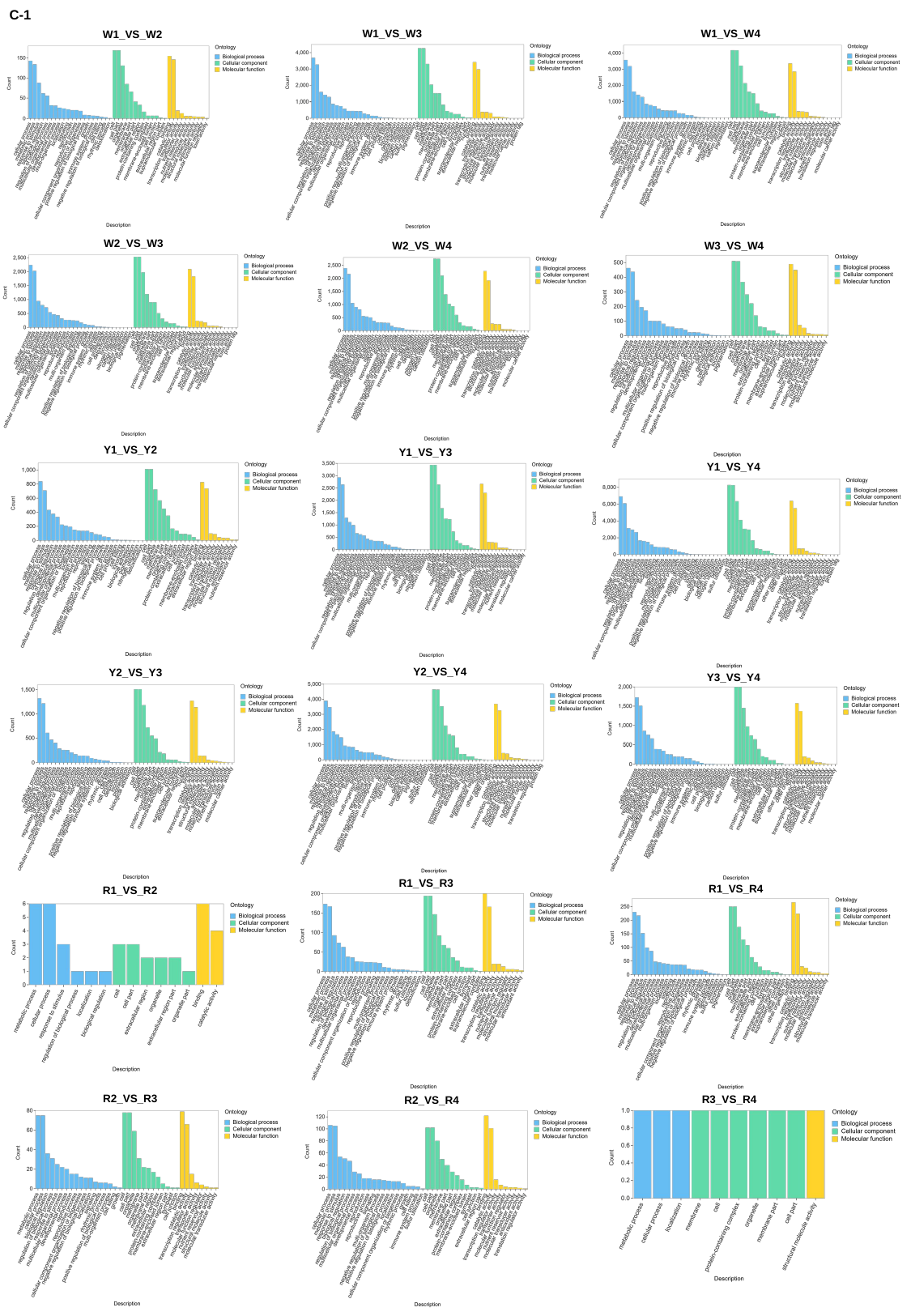

C-1
W1_VS_W3
W1_VS_W4
W1_VS_W2
W2_VS_W3
W2_VS_W4
W3_VS_W4
Y1_VS_Y2
Y1_VS_Y3
Y1_VS_Y4
Y2_VS_Y4
Y2_VS_Y3
Y3_VS_Y4
R1_VS_R3
R1_VS_R4
R1_VS_R2
R2_VS_R3
R3_VS_R4
R2_VS_R4

## Slide 12
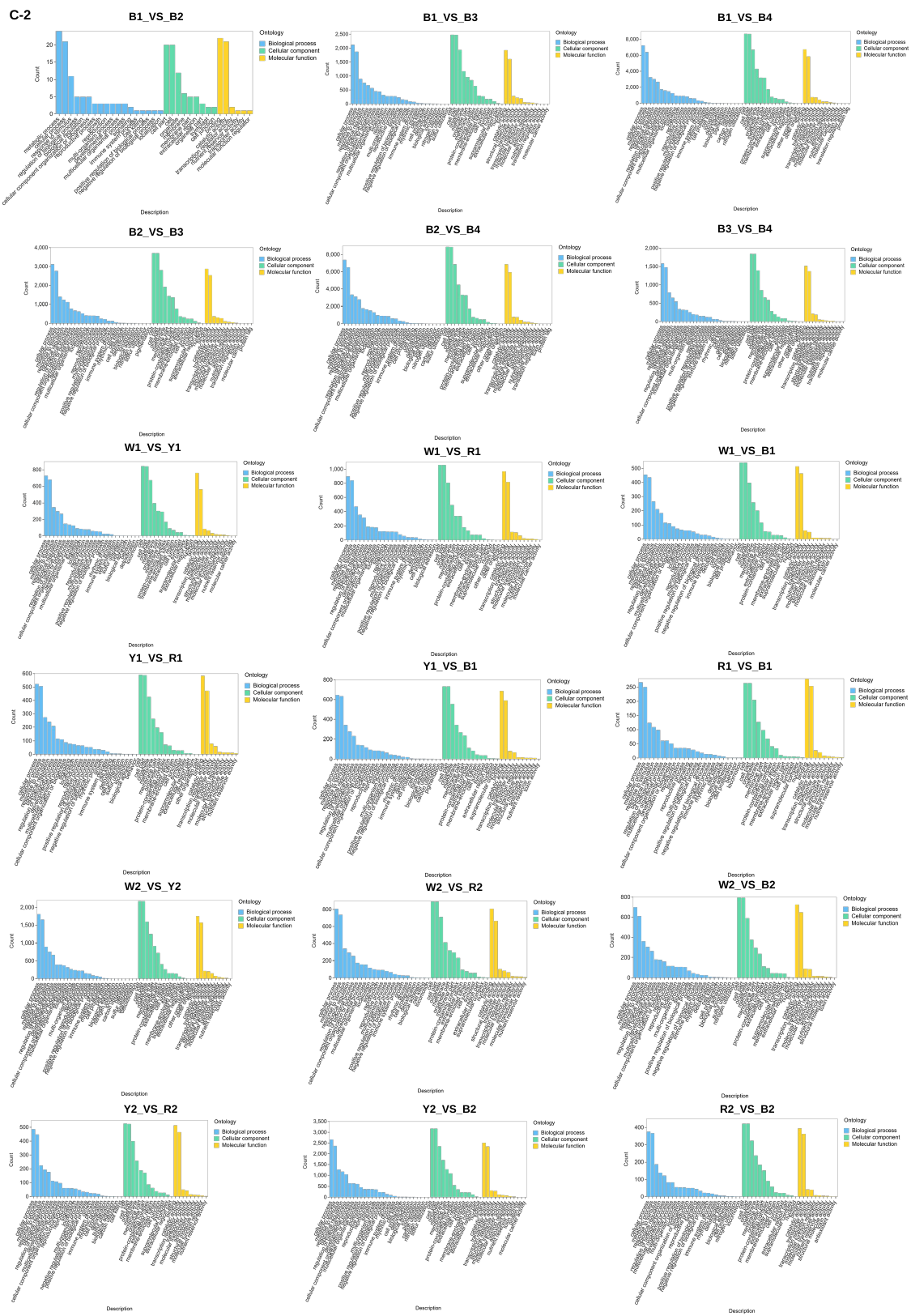

C-2
B1_VS_B2
B1_VS_B3
B1_VS_B4
B3_VS_B4
B2_VS_B4
B2_VS_B3
W1_VS_Y1
W1_VS_B1
W1_VS_R1
Y1_VS_R1
Y1_VS_B1
R1_VS_B1
W2_VS_B2
W2_VS_Y2
W2_VS_R2
R2_VS_B2
Y2_VS_R2
Y2_VS_B2

## Slide 13
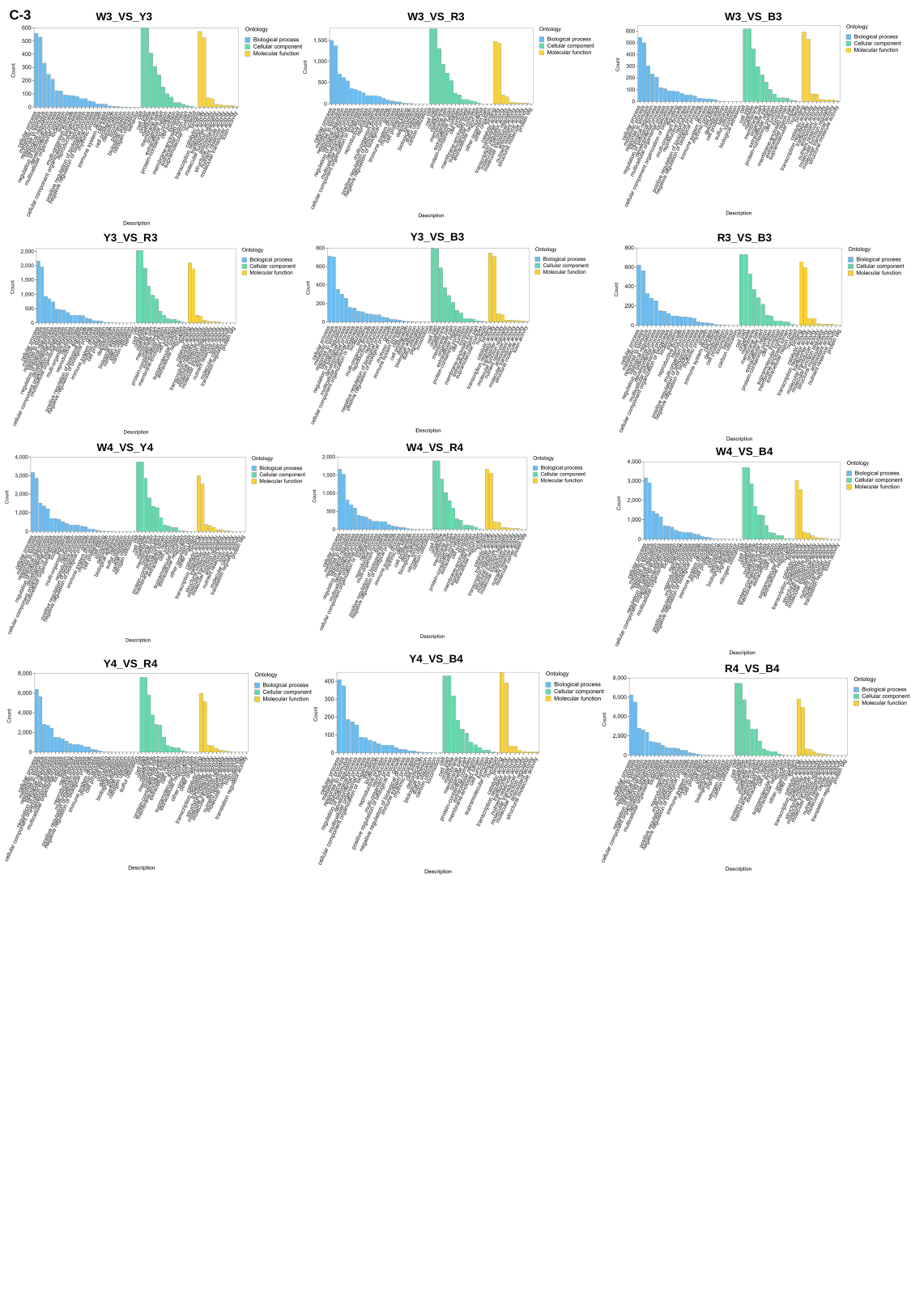

C-3
W3_VS_Y3
W3_VS_R3
W3_VS_B3
Y3_VS_B3
Y3_VS_R3
R3_VS_B3
W4_VS_Y4
W4_VS_R4
W4_VS_B4
Y4_VS_B4
Y4_VS_R4
R4_VS_B4

## Slide 14
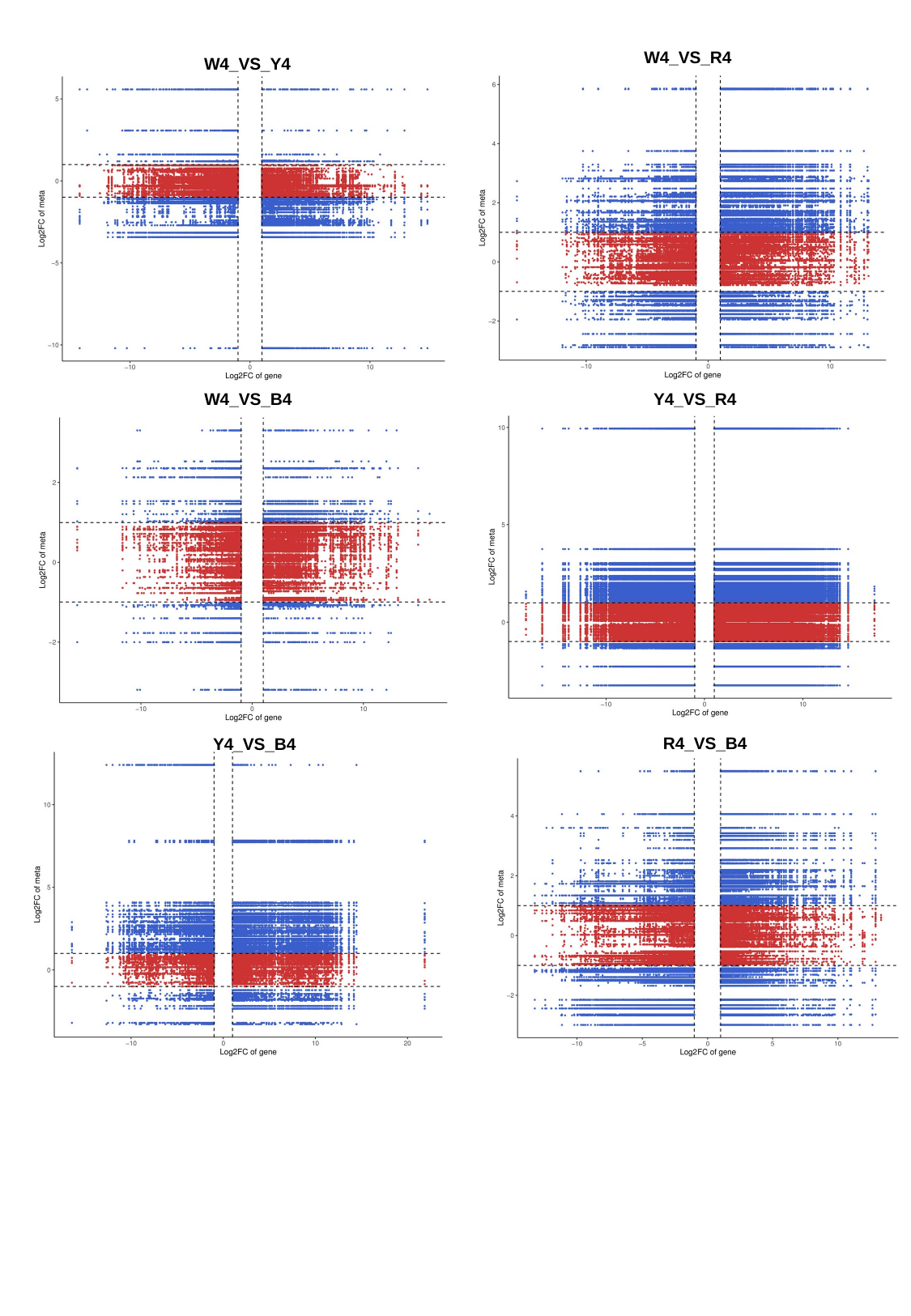

W4_VS_R4
W4_VS_Y4
Y4_VS_R4
W4_VS_B4
R4_VS_B4
Y4_VS_B4
