# Supplementary material for: Metabolome and Transcriptome Association Analysis Reveals Mechanism of Synthesis of Nutrient Composition in Quinoa (Chenopodium quinoa Willd.) Seeds
Source: Foods. 2024 Apr 26;13(9):1325. doi: 10.3390/foods13091325 (PMC11082971; doi:10.3390/foods13091325)
Supplement: Supplementary file 1 [file foods-13-01325-s001.zip › Table S5.pdf]

Table S5. Differential expression of carbon metabolism and regulatory genes in four quinoa cultivars

	Name	ID	log2FoldChange	regulated	KEGG
Y_VS_R		gene-LOC110685808	1.984184284	up	K01792 glucose-6-phosphate 1-epimerase
Y_VS_R	glucose-6-phosphate 1-epimerase	gene-LOC110704761	3.036065833	up	K01792 glucose-6-phosphate 1-epimerase
Y_VS_R		gene-LOC110707537	3.9918045	up	K01792 glucose-6-phosphate 1-epimerase
Y_VS_R		gene-LOC110727040	1.079349106	up	K01785 aldose 1-epimerase
Y_VS_R	aldose 1-epimerase	gene-LOC110727040	1.575752533	up	K01785 aldose 1-epimerase
Y_VS_R		gene-LOC110685808	1.984184284	up	K01792 glucose-6-phosphate 1-epimerase
Y_VS_R	6-phosphofructokinase 1	gene-LOC110736626	2.055880414	up	K00850 6-phosphofructokinase 1
Y_VS_R		gene-LOC110694871	1.100636504	up	K01102 pyruvate dehydrogenase phosphatase
Y_VS_R		gene-LOC110697295	1.295637853	up	K01102 pyruvate dehydrogenase phosphatase
Y_VS_R	pyruvate dehydrogenase	gene-LOC110698093	1.108074132	up	K00161 pyruvate dehydrogenase E1 component alpha subunit
Y_VS_R		gene-LOC110703927	2.82845892	up	K00162 pyruvate dehydrogenase E1 component beta subunit
Y_VS_R		gene-LOC110711593	2.212996713	up	K00162 pyruvate dehydrogenase E1 component

					beta subunit
Y_VS_R		gene-LOC110738218	1.42691216	up	K01102 pyruvate dehydrogenase phosphatase
Y_VS_R		gene-LOC110739657	2.299423621	up	K00162 pyruvate dehydrogenase E1 component beta subunit
Y_VS_R		gene-LOC110691796	1.437561632	up	K00592 fructose-bisphos phate aldolase
Y_VS_R		gene-LOC110705177	3.839910634	up	K01623 fructose-bisphos phate aldolase
Y_VS_R	fructose-bisphosphate aldolase	gene-LOC110712665	1.582636837	up	K19199 histone-lysine N-methyltransfer ase
Y_VS_R		gene-LOC110718872	3.766159744	up	K01623 fructose-bisphos phate aldolase
Y_VS_R		gene-LOC110730842	1.762210155	up	K00592 fructose-bisphos phate aldolase
Y_VS_R		gene-LOC110734726	1.178846637	up	K00592 fructose-bisphos phate aldolase
Y_VS_R		gene-LOC110710556	3.717704575	up	K00008 L-iditol 2-dehydrogenase
Y_VS_R		gene-LOC110712843	2.790907753	up	K00008 L-iditol 2-dehydrogenase
Y_VS_R		gene-LOC110695210	1.10012886	up	K00083 cinnamyl-alcoho l dehydrogenase
Y_VS_R	alcohol dehydrogenase	gene-LOC110705193	1.119711259	up	K00002 alcohol dehydrogenase
Y_VS_R		gene-LOC110708308	1.448936541	up	K18857 alcohol dehydrogenase
Y_VS_R		gene-LOC110710556	2.246039084	up	K00008 L-iditol 2-dehydrogenase
Y_VS_R		gene-LOC110712843	2.683189907	up	K00008 L-iditol 2-dehydrogenase

Y_VS_R	gene-LOC110718807	1.048278939	up	K00083 cinnamyl-alcohol dehydrogenase
Y_VS_R	gene-LOC110733075	2.381281703	up	K22395 cinnamyl-alcohol dehydrogenase
Y_VS_R	gene-LOC110735365	3.831269208	up	K00002 alcohol dehydrogenase
Y_VS_R	gene-LOC110699057	1.839183609	up	K00121 alcohol dehydrogenase
Y_VS_R	gene-LOC110710556	4.303312847	up	K00008 L-iditol 2-dehydrogenase
Y_VS_R	gene-LOC110712843	4.82393127	up	K00008 L-iditol 2-dehydrogenase
Y_VS_R	gene-LOC110735199	1.66877054	up	K00083 cinnamyl-alcohol dehydrogenase
Y_VS_R	gene-LOC110682049	3.488688792	up	K00083 cinnamyl-alcohol dehydrogenase
Y_VS_R	gene-LOC110686162	2.329274466	up	K22395 cinnamyl-alcohol dehydrogenase [EC:1.1.1.195] | (RefSeq) berberine bridge enzyme-like 15 (A)
Y_VS_R	gene-LOC110689684	1.362246104	up	K00121 alcohol dehydrogenase
Y_VS_R	gene-LOC110699057	3.636244473	up	K00121 alcohol dehydrogenase
Y_VS_R	gene-LOC110709669	6.317766577	up	K00121 alcohol dehydrogenase
Y_VS_R	gene-LOC110718574	2.020160613	up	K22395 cinnamyl-alcohol dehydrogenase
Y_VS_R	gene-LOC110721627	1.351241739	up	K00121 alcohol dehydrogenase
Y_VS_R	gene-LOC110727823	2.502017241	up	K00083 cinnamyl-alcoho

					l dehydrogenase
Y_VS_R		gene-LOC110735365	5.256723089	up	K00002 alcohol dehydrogenase (NADP+) [EC:1.1.1.2] | (RefSeq) NADPH-dependent aldo-keto reductase, chloroplastic-like (A)
Y_VS_R	hexokinase	novel.7213	1.222906668	up	K00844 hexokinase
Y_VS_R		novel.5000	1.800439413	up	K00844 hexokinase
Y_VS_R	hexokinase	gene-LOC110725781	-3.976529981	down	K01637 isocitrate lyase
Y_VS_R		gene-LOC110735795	-4.284403241	down	K01637 isocitrate lyase
Y_VS_R		gene-LOC110725782	-5.787801482	down	K01637 isocitrate lyase
Y_VS_R		gene-LOC110735796	6.403188413	up	K01637 isocitrate lyase
Y_VS_R	Ribulose bisphosphate carboxylase small chain	gene-LOC110720402	2.352567137	up	K01602 ribulose-bisphosphate carboxylase small chain
Y_VS_R		gene-LOC110720403	5.712464186	up	K01602 ribulose-bisphosphate carboxylase small chain
Y_VS_R		gene-LOC110720405	2.864711639	up	K01602 ribulose-bisphosphate carboxylase small chain
Y_VS_R		gene-LOC110728206	6.082795418	up	K01602 ribulose-bisphosphate carboxylase small chain
Y_VS_R	UDP-sugar pyrophosphorylase	novel.3959	8.658161339	up	K12447

					UDP-sugar pyrophosphorylase
Y_VS_R		novel.4763	2.415740235	up	K12447 UDP-sugar pyrophosphorylase
Y_VS_R		gene-LOC110729741	2.114773024	up	K01835 phosphoglucomutase
Y_VS_R	phosphoacetylglucosamine mutase	gene-LOC110732480	1.304204785	up	K01836 phosphoacetylglucosamine mutase
W1_VS_W3	phosphoglycerate kinase	gene-LOC110713524	2.624255036	up	K00927 phosphoglycerate kinase
Y1_VS_Y4		gene-LOC110721314	3.294708741	up	K00927 phosphoglycerate kinase
Y4_VS_R4	pyruvate dehydrogenase E1 component beta subunit	gene-LOC110728546	2.312122182	up	K00162 pyruvate dehydrogenase E1 component beta subunit
Y4_VS_R4		gene-LOC110721444	2.090774576	up	K00615 transketolase
Y4_VS_B4	pyruvate dehydrogenase E1 component beta subunit	gene-LOC110732515	2.208506002	up	K00873 pyruvate kinase
Y_VS_B	granule-bound starch synthase	gene-LOC110722789	1.556701588	up	K13679 granule-bound starch synthase
Y_VS_B		gene-LOC110730395	1.361791392	up	K00703 starch synthase
Y_VS_B		gene-LOC110732328	1.129575282	up	K13679 granule-bound starch synthase
Y_VS_B		gene-LOC110738898	2.916723874	up	K13679 granule-bound starch synthase
Y_VS_B	starch synthase	gene-LOC110687372	1.670965318	up	K00703 starch synthase
Y_VS_B		gene-LOC110722789	1.556701588	up	K13679 granule-bound

					starch synthase
Y_VS_B		gene-LOC110730395	1.361791392	up	K00703 starch synthase
Y_VS_B		gene-LOC110731330	1.767628501	up	K00703 starch synthase
Y_VS_B		gene-LOC110732328	1.129575282	up	K13679 granule-bound starch synthase
Y_VS_B		gene-LOC110738898	2.916723874	up	K13679 granule-bound starch synthase
Y_VS_B		gene-LOC110683757	1.042069007	up	K00703 starch synthase
Y_VS_B		gene-LOC110718929	2.367146266	up	K01176 alpha-amylase
Y_VS_B		gene-LOC110718424	-4.576371533	down	K01176 alpha-amylase
	alpha-amylase				
Y_VS_B		gene-LOC110738785	-2.48999058	down	K01176 alpha-amylase
Y_VS_B		gene-LOC110737712	-5.024549741	down	K01176 alpha-amylase
Y_VS_B		gene-LOC110691952	-2.650292542	down	K01177 beta-amylase
Y_VS_B		gene-LOC110692055	-5.726130035	down	K01177 beta-amylase
Y_VS_B		gene-LOC110730742	-3.757466597	down	K01177 beta-amylase
Y_VS_B		gene-LOC110730910	-4.328162689	down	K01177 beta-amylase
Y_VS_B		gene-LOC110732446	1.163339555	up	K01177 beta-amylase
Y_VS_B	beta-amylase	gene-LOC110691737	-1.125403941	down	K01177 beta-amylase
Y_VS_B		gene-LOC110694254	3.663847362	up	K01177 beta-amylase
Y_VS_B		gene-LOC110696059	1.621824586	up	K01177 beta-amylase
Y_VS_B		gene-LOC110710941	1.673165356	up	K01177 beta-amylase
Y_VS_B		gene-LOC110730263	1.9802322	up	K01177 beta-amylase
Y_VS_B		gene-LOC110736249	1.108397916	up	K01177 beta-amylase
Y_VS_B	sugar transporter	gene-LOC110685535	1.434678131	up	K08150 MFS

				transporter,
Y_VS_B	gene-LOC110706483	1.333033756	up	K15382 solute carrier family 50 (sugar transporter)
Y_VS_B	gene-LOC110716439	-2.088430089	down	K08145 MFS transporter
Y_VS_B	gene-LOC110720835	-3.574958524	down	K15281 solute carrier family 35
Y_VS_B	gene-LOC110725786	1.247396602	up	K15382 solute carrier family 50 (sugar transporter)
Y_VS_B	gene-LOC110735791	1.710884542	up	K15382 solute carrier family 50 (sugar transporter)
Y_VS_B	gene-LOC110739666	3.987716265	up	K15382 solute carrier family 50 (sugar transporter)
W3_VS_W4	gene-LOC110705141	4.174693816	up	K08150 MFS transporter
